# Novel 4,5‐Dihydrothiazole‐Phenylpiperazine Derivatives: Synthesis, Docking Studies and Pharmacological Evaluation as Serotonergic Agents

**DOI:** 10.1002/cmdc.202500288

**Published:** 2025-07-04

**Authors:** Giorgia Andreozzi, Natalia Karkoszka, Rosa Sparaco, Angela Corvino, Beatrice Severino, Vincenzo Santagada, Elisa Magli, Ewa Gibuła‐Tarłowska, Jolanta H. Kotlińska, Kinga Gawel, Raffaele Capasso, Anna Lesniak, Nataliia Semenko, Agnieszka A. Kaczor, Anna Bielenica, Grażyna Biała, Giuseppe Caliendo, Ewa Kędzierska, Ferdinando Fiorino

**Affiliations:** ^1^ Dipartimento di Farmacia Università degli Studi di Napoli Federico II Via D. Montesano 80131 Napoli Italy; ^2^ Department of Pharmacology and Pharmacodynamics Medical University of Lublin Chodźki 4a Str 20‐093 Lublin Poland; ^3^ Department of Experimental and Clinical Pharmacology Medical University of Lublin Jaczewskiego Str. 8b 20‐090 Lublin Poland; ^4^ Dipartimento di Sanità Pubblica Università di Napoli Federico II Via Pansini, 5 80131 Naples Italy; ^5^ Dipartimento di Agraria Università degli Studi di Napoli “Federico II” Via Università 100, Portici 80055 Naples Italy; ^6^ Department of Pharmacodynamics Faculty of Pharmacy Medical University of Warsaw Centre for Preclinical Research and Technology Warsaw Poland; ^7^ Department of the Surgery Anesthesiology and Intensive care Bogomolets National Medical University Kyiv Ukraine; ^8^ Department of Synthesis and Chemical Technology of Pharmaceutical Substances with Computer Modeling Laboratory Faculty of Pharmacy Medical University of Lublin 4A Chodzki St. 20‐093 Lublin Poland; ^9^ School of Pharmacy University of Eastern Finland Yliopistonranta 1, P.O. Box 1627 70211 Kuopio Finland; ^10^ Chair and Department of Biochemistry Medical University of Warsaw Banacha 1 Str 02‐097 Warsaw Poland

**Keywords:** (4,5‐Dihydrothiazol‐2‐yl) phenyl) piperazines, binding assays, ligand design, serotonin, structure–activity relationships

## Abstract

The synthesis of a new series of long‐chain arylpiperazine as serotoninergic ligands (**FG 1‐18**) is described. The combination of structural elements including heterocyclic nucleus, propyl chain, and 4,5‐dihydrothiazol‐2‐ylphenylpiperazines leads to the preparation of different derivatives tested for their affinity toward 5‐HT_1A_, 5‐HT_2A_, and 5‐HT_2C_ receptors. The compounds with better affinity and selectivity binding profiles toward 5‐HT_1A_ and 5‐HT_2C_ (**FG‐1**, **FG‐4**, **FG‐5**, **FG‐6**, **FG‐7**, **FG‐8**, and **FG‐18**) are selected for further *in vivo* assays to determine their functional activity. Finally, to rationalize the obtained results, molecular docking studies are performed. The results of pharmacological studies show that compounds **FG‐1**, **FG‐5**, **FG‐8**, and **FG‐6** exert antidepressant‐like effects, and **FG‐1**, **FG‐18**, **FG‐6**, and **FG‐7** reveal also significant anxiolytic properties. Among the developed derivatives, the most promising compounds seem to be **FG‐1**, which exhibit antidepressant, anxiolytic, and anticonvulsant properties, **FG‐7** and **FG‐18** that show features as anxiolytic combine to a pro‐cognitive property and notable affinity and selectivity for 5‐HT_2C_ receptor, respectively.

## Graphical abstract

The synthesis of a new series of 4,5‐dihydrothiazol‐2‐ylphenylpiperazine derivatives as serotoninergic ligands is described and tested for their affinity toward 5‐HT_1A_, 5‐HT_2A_,and 5‐HT_2C_ and selected for further in vivo assays to determine their functional activity. The pharmacological studies highlight that among the developed derivatives, **FG‐7** and **FG‐18** show notable features as anxiolytic combine to pro‐cognitive property.

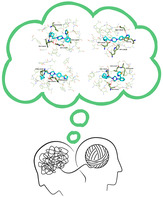

## 1 Introduction

**Figure 1:**
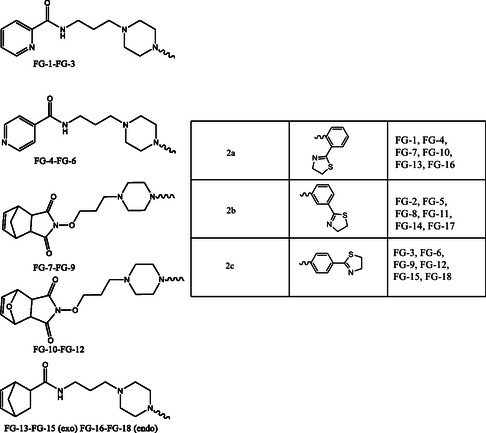
Chemical structures of the synthesized compounds **FG 1‐18.**

Serotonin (5‐hydroxytryptamine, 5‐HT) is a monoamine that acts as a neurotransmitter in the central nervous system (CNS). First, through a two‐step enzymatic process, it is synthesized from the amino acid tryptophan. Nowadays, it is well established that this molecule is involved in the regulation of numerous CNS processes, including brain maturation, temperature regulation, learning, pain modulation, appetite, sexual behavior, emotional responses, aggression, motor control, and hormone secretion. This is the reason why serotonin pathways are common targets of drugs such as antidepressants, antipsychotics, and hallucinogens.^[^ ^1^ ^]^ In addition to its neurological functions, serotonin receptors are also widely expressed in several types of cancer, and one of the mechanisms by which serotonin regulates proliferation seems to be the MAPK/ERK and PI3K/Akt pathway.^[^ ^2^ ^]^ It is already widely known that serotonin receptors are classified into seven families that differentiate by structural features and signaling pathways and, except for the 5‐HT_3_ receptor, ion channel, all serotonin receptors belong to the G‐protein‐coupled receptor (GPCR) family. 5‐HT_1A_ receptor is one of the most prevalent serotonin receptors in the brain. As a GPCR, its primary response to serotonin binding is the activation of hyperpolarizing potassium channels (K^+^). Furthermore, this receptor participates in several molecular pathways, including regulation of phospholipase‐C activity, inhibition of cAMP accumulation, and reduction of calcium currents. In terms of distribution, 5‐HT_1A_ receptor has been accurately mapped in the brain through techniques such as receptor autoradiography and, more recently, positron emission tomography (PET).^[^ ^3^ ^]^ On the other hand, 5‐HT_2A_ receptor is likewise a member of the G‐protein family and is extensively distributed in the CNS. Second, activation of neuronal 5‐HT_2A_ receptors can trigger various effects through G‐protein‐dependent, ligand‐dependent, and ligand‐independent signaling pathways. These involve mechanisms that implicate phospholipase activity, the ERK signaling pathway, and tyrosine kinase signaling in neurons. Normally, activation of 5‐HT_2A_Rs raises intracellular calcium (Ca^2+^) levels via the Gαq‐PLC‐IP3 pathway. Along with interacting with G‐proteins, 5‐HT_2A_Rs are also linked to β‐arrestin2. When serotonin binds to the 5‐HT_2A_R, it can initiate the phosphorylation of Akt via the β‐arrestin2/phosphoinositide 3‐kinase (PI3K)/Src/Akt signaling cascade.^[^ ^4^ ^]^ The 5‐HT_2A_ receptor is expressed in various regions of the CNS where it has excitatory or inhibitory functions, depending on anatomical location. Finally, 5HT_2C_R, which is widely distributed in the CNS, plays a decisive role in the patho‐physiology of psychiatric diseases^[^ ^5^ ^]^ and further is a validated therapeutic target for several disorders including depression, schizophrenia, and drug addiction. 5‐HT receptors are, therefore, well‐known therapeutic targets; in particular, the previously mentioned receptor subtypes widely distributed in the brain are involved in the treatment of neurological disorders, in oncology, and in numerous pathological conditions, so much so that research in this field is constantly evolving, with the development of new compounds aimed at improving the selectivity, efficacy, and safety profile of drugs. Among the classes of drugs and pharmacologically active molecules, arylpiperazine ligands are known to modulate the activity of 5‐HT_1A_, 5‐HT_2A_, and 5‐HT_2C_ receptors.^[^ ^6^ ^]^ The arylpiperazine moiety represents a chemical scaffold of great importance in the design of new drugs, with obvious versatility and therapeutic potential. The long‐chain aryl piperazines (LCAPs) represent a valuable source of 5‐HT_1A_ receptor ligands. The binding modes of several FDA‐approved antidepressants in the 5‐HT_1A_ receptor pocket shared features, such as a salt bridge with Asp116 (3.32), CH–π or π–π interactions with Phe361 (6.51), and π–π stacking interactions with Phe362 (6.52).^[^ ^7^ ^]^ This scaffold is also recurrent in the modulation of the 5‐HT_2A_ receptor.^[^ ^8^ ^]^ Its chemical properties and pharmacokinetic profile have made it an important pharmacophore in drug design, and a core scaffold based on which numerous drugs has been synthesized and as many are in the development phase. It has been observed that when piperazine is positioned as a bridge between two heteroaromatic groups, it increases its affinity for serotonergic receptors through key interactions, such as salt bridges with Asp3.32 in the binding site of the 5‐HT_1A_ receptor, where the protonatable nitrogen atom of the piperazine ring plays a crucial role in ion binding, while CH–π interactions with Phe6.52 and π–π interactions with other aromatic residues of the receptor contribute to the stability of the complex.^[^ ^9^ ^]^ The affinity and selectivity of these ligands can be optimized through targeted structural modifications, such as the presence of different substituents on the aromatic ring of 4‐substituted phenylpiperazine, modification of the alkyl chain length, and the nature of the heterocyclic fragment as terminal part of LCAPs. Research is currently focused on the design of more selective and active compounds; in fact, depending on their structure, they can act as agonists, antagonists or partial agonists, and consequently their selectivity for specific receptor subtypes is crucial to minimize side effects in different diseases. We have already demonstrated that, first, the length of the alkyl chain between aryl‐piperazine moiety and heterocyclic scaffold of LCAPs affects both the affinity and selectivity for different 5‐HT receptors. At the same time, the nature of the substituents on the phenyl ring of N‐4‐substituted piperazine can modulate the polarity and ability to form hydrogen bonds, affecting the interaction with the receptor; in addition the introduction of several nuclei such as norbornene, isonicotinamide, or picolinic nuclei as a terminal moiety can increase the affinity and selectivity for the 5‐HT_1A_, 5‐HT_2A_, and 5‐HT_2C_ receptors.^[^ ^10^, ^11^, ^12^, ^13^ ^]^ Consequently, in our laboratories, we have repeatedly studied and compared the role of the piperazine scaffold as well as other nuclei linked to it. In continuation to our research projects and to further investigate the influence of different nuclei in interacting with serotonergic receptors, we synthesized eighteen compounds (**FG 1‐18**) as LCAPs (**Figure** **1**) resulting from the combination of five different scaffolds, already investigated in our studies, with a new arylpiperazine fragment.

**Figure 2:**
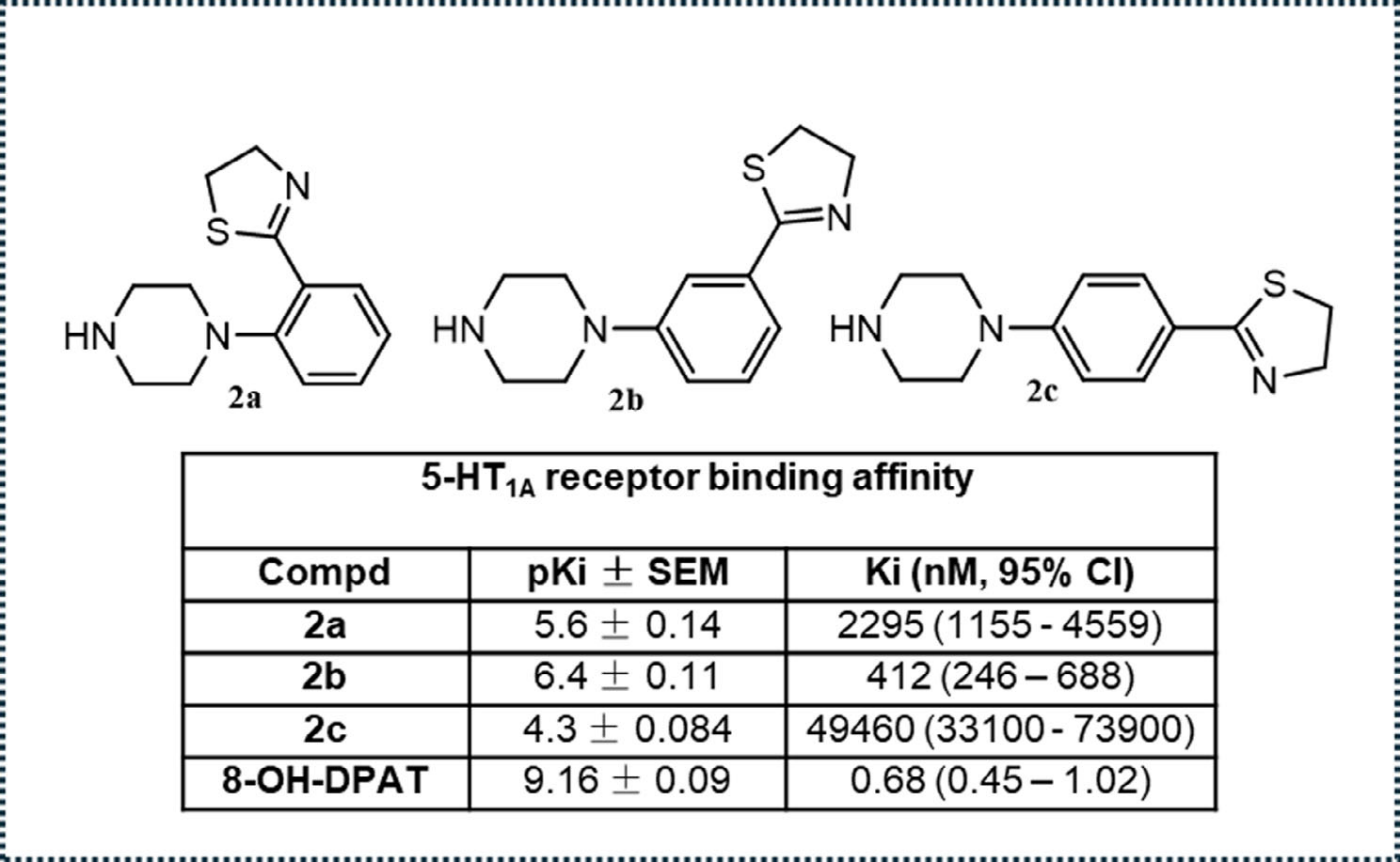
Previously synthesized compounds **2a**‐**2c** and the corresponding 5‐HT_1A_ receptor binding affinity.

This moiety, synthesized for the first time by our research group, represents a new arylpiperazine scaffold supporting a 4,5‐dihydrothiazole substituent on all the possible positions of the aromatic ring (**Figure** **2**). This choice was made to investigate how the introduction of the 4,5‐dihydrothiazole moiety might influence the affinity/activity profile towards serotonergic receptors; moreover, this scaffold was already tested to study its influence to modulate 5‐HT_1A_, 5‐HT_2A_, and 5‐HT_2C_ serotoninergic receptors involved in regulatory pathways of prostate and breast cancer cells viability.^[^ ^14^ ^]^ All the new synthesized compounds, reported in this study, were tested for their functional activity or affinity to 5‐HT_1A_, 5‐HT_2A_, and 5‐HT_2C_ receptors. Moreover, compounds showing the best affinity and selectivity binding profiles toward serotoninergic receptors were evaluated by in vivo assay through behavioral tests, with the aim to discover novel pharmacological tools useful in treating psychiatric and neurological disorders, such as schizophrenia, depression, and anxiety. Therefore, we evaluated the antipsychotic activity of the compounds in an amphetamine‐induced hyperactivity test, antidepressant‐like activity in the forced swim test (FST), and anxiolytic‐like effects in the elevated plus‐maze test (EPM). Additional tests, as the spontaneous locomotor activity, rota‐rod, and chimney tests, have been used to assess potential adverse effects of the compounds. Finally, to assess the anxiolytic and anticonvulsant profile of the new derivatives, studies were also carried out on larval zebrafish (*Danio rerio*) as an alternative vertebrate model.

## 2 Results and Discussion

### 2.1 Chemistry

**Scheme 1:**
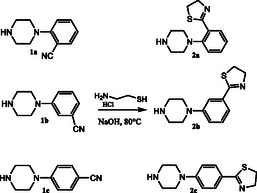
Synthetic route of thiazolinylphenyl‐piperazines **2a‐2c**.

In the previous research, we reported the synthesis of 1‐(2‐thiazolinylphenyl) piperazine (**2a**), 1‐(3‐thiazolinylphenyl) piperazine (**2b**) and 1‐(4‐thiazolinylphenyl) piperazine (**2c**). The general strategy for the synthesis of these compounds is summarized in **Scheme** **1**. Treatment of commercially available 4‐substituted piperazines (**1a‐c**), with 2‐aminoethane‐1‐thiol hydrochloride in presence of NaOH under solvent‐free conditions heating to 80 °C, gave the corresponding thiazolinylphenyl‐piperazines (**2a‐c**).

**Scheme 2:**
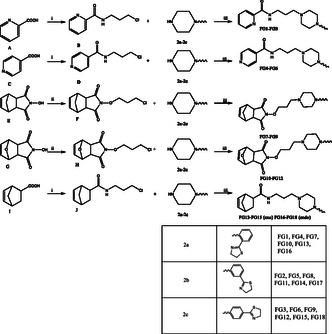
Reagents and conditions: (i): DCC, HOBt, TEA, Cl(CH_2_)_3_NH_2_·HCl, CH_3_CN, 24 h; (ii): Br(CH_2_)_3_Cl, NaOH, EtOH abs; (iii): K_2_CO_3_, NaI, CH_3_CN, reflux, 24 h.

The general strategy for the synthesis of the target compounds is summarized in **Scheme** **2**. Picolinic acid (**A**), isonicotinic acid (**C**), norbornenic acid (**I**) reacted with 3‐chloro propan‐1‐amine hydrochloride in acetonitrile, in the presence of N,N’‐dicyclohexylcarbodiimide (DCC), 1‐hydroxybenzotriazole (HOBt), and triethylamine (TEA) to give the corresponding chloropropyl derivatives (**B**,**D**,**J**); instead, the starting *N*‐hydroxy‐5‐norbornene‐2,3‐dicarboximide (**E**) and 2‐hydroxy‐3a,4,7,7a‐tetrahydro‐4,7‐epoxyisoindole‐1,3‐dione (**G**) were alkylated with 1‐bromo‐3‐chloropropane in the presence of NaOH in absolute ethanol to give the corresponding chloropropyl intermediates (**F** and **H**). Subsequently, condensation of intermediates **B**, **D**, **F**, **H**, **J** with the 4‐substituted‐piperazine (**2a**,**2b**,**2c**), performed in CH_3_CN in the presence of K_2_CO_3_ and NaI, under reflux, provided the final compounds (**FG 1‐18**). Purification of each final product was performed by chromatography on a silica gel column and further by crystallization from the appropriate solvent. All the new compounds were characterized by satisfactory elemental analyses and were characterized by ^1^H NMR ^13^C NMR and MS, obtaining data consistent with the proposed structures.

### 2.2 Prediction of Activity Spectra for Substances

In order to carry out a preliminary validation of the designed molecules as serotonergic ligands, we decided to approach with an *in silico* prediction analysis using the Prediction of Activity Spectra for Substances (PASS) software to estimate the potential biological profile of the designed derivatives. This approach is based on a suggestion of activity and function by comparing the 2D structure of a test compound with biologically active substances. The predicted activity spectrum is presented in PASS by the list of activities with probabilities “to be active” Pa and “to be inactive” Pi calculated for each activity). The list is arranged in descending order of Pa‐Pi; thus, the more probable activities appear at the top of the list. Only activities with Pa > Pi are considered as possible for a particular compound.^[^ ^15^ ^]^ On these bases, the investigated derivatives (**FG 1**‐**18)** showed interesting results later confirmed by in vitro and in vivo pharmacological studies. Consequently, the PASS analysis highlighted a preliminary interesting profile as serotonergic ligands for all the considered derivatives. In particular, this analysis revealed among the pharmacological effects, 5‐HT_1A_ agonism (P_a_: 0.059) for compound **FG‐5** supporting an isonicotinic nucleus. Furthermore, this activity profile was found to be better in the derivative **FG‐7** (Pa: 0.101) characterized by an endo‐N‐hydroxy‐5‐norbornene‐2,3‐dicarboximide scaffold, which also associates an anxiolytic effect (Pa: 0.631) at the top of the PASS generated list of the predicted activities. Anyway, by moving the 4,5‐dihydrothiazole substituent from the ortho position (**FG‐7**) to the meta position (**FG‐8**), a decrease in the predictive value Pa of 5‐HT_1A_ agonism (Pa: 0.086) and an additional effect as an antidepressant (Pa: 0.320) was evidenced. Finally, compound **FG‐18,** a 5‐norbornene‐2‐carboxamide derivative, presented a mixed 5‐HT_1A_ agonism/antagonism profile (P_a_: 0.054 and P_a_: 0.147, respectively) and a lower value (P_a_: 0.170) as anxiolytic profile compared to the previous compounds. Furthermore, in the preliminary predictive analysis, the compound **FG‐8** also appeared to be characterized by an interesting antagonistic profile toward 5‐HT_2C_ receptors (Pa:0.254) showing overall an interesting mixed 5‐HT_1A_/5‐HT_2C_ activity profile. These results support the design choice made for the preparation of serotoninergic ligands.

### 2.3 5‐HT_1A,_ 5‐HT_2A_ and 5‐HT_2C_ Receptor Binding

All the new compounds were tested for their affinity at the 5‐HT_1A_ (**Table** **1**), 5‐HT_2A_, and 5‐HT_2C_ (**Table** **2**) receptors. Some of the new synthesized derivatives showed interesting affinity values, falling within the nanomolar range toward 5‐HT_1A_ and 5‐HT_2C_ receptors and lower affinities for 5‐HT_2A_ receptors. Apart from the outstanding 5‐HT_2C_ receptor affinity and selectivity of compounds **FG‐18** (Ki = 17 nM) and **FG‐8** (Ki = 46 nM), compound **FG‐14** (72 nM) is also of interest. Moreover, compounds **FG‐7** and **FG‐16** showed interesting selectivity profiles toward 5‐HT_1A_ receptors with Ki values of 54 and 25 nM, respectively. Whereas compound **FG‐14** presented an attractive mixed 5‐HT_2A_/5‐HT_2C_ affinity with K_i_ value of 430 and 72 nM, respectively. These results, as already demonstrated in previous studies, further support the choice of the endo‐*N*‐hydroxy‐5‐norbornene‐2,3‐dicarboximide^[^ ^12^ ^]^ and 5‐norbornene‐2‐carboxamide scaffold^[^ ^9^ ^]^ for the preparation of serotoninergic ligands endowed with a high 5‐HT_1A_ affinity, associated with a high selectivity towards 5‐HT_2A_ and 5‐HT_2C_ receptors. Concerning the influence of the substituent on the N‐4 atom of the piperazine ring, the 2‐(4,5‐dihydrothiazol‐2‐yl) phenyl moiety present in compounds (**FG‐7** and **FG‐16**) produced the highest affinity for the 5‐HT_1A_ receptor. These results further confirm how, in LCAPs derivatives, the *orto* substitution on the aromatic ring of the aryl piperazine residue is favorable for the interaction with the 5‐HT_1A_ receptor subtype. Instead, the presence of (4,5‐dihydrothiazol‐2‐yl) substituent in *para* position of the phenyl moiety linked to the N‐4 atom of the piperazine ring combined to a 5‐norbornene‐2‐carboxamide residue conferred the most attractive 5‐HT_2C_ affinity/selectivity profile. These data are very interesting considering the high degree of homology existing between the considered receptors and demonstrates that this compound (**FG‐18**) possesses a very promising binding profile. To better understand the differences in binding affinities of the new arylpiperazine derivatives, molecular modeling experiments were conducted.

**Table 1:** Binding affinity values of compounds **FG 1‐18** for 5‐HT_1A_ receptor.

5‐HT_1A_ receptor binding affinity		
Compd.	5‐HT_1A_ pKi	5‐HT_1A_ Ki (95% CI, nM)
**FG‐1**	5.63 ± 0.11	2350 (1330–4050)
**FG‐2**	5.73 ± 0.11	1860 (1100–3200)
**FG‐3**	5.34 ± 0.09	4500 (2930–6970)
**FG‐4**	5.81 ± 0.08	1540 (1050–2250)a)
**FG‐5**	5.96 ± 0.12	1100 (610–1990)a)
**FG‐6**	4.58 ± 0.21	2640 (9400–74 100)
**FG‐7**	7.3 ± 0.07	54 (35–69)a)
**FG‐8**	6.14 ± 0.14	710 (370–1340)
**FG‐9**	4.14 ± 0.12	70 900 (38 900–12 900)
**FG‐10**	6.21 ± 0.11	610 (350–1050)a)
**FG‐11**	5.66 ± 0.12	2200 (1200–4000)
**FG‐12**	4.20 ± 0.09	52 500 (33 200–83 200)
**FG‐13**	6.66 ± 0.1	220 (130–360)
**FG‐14**	6.47 ± 0.07	340 (240–480)
**FG‐15**	4.75 ± 0.11	17 700 (10 400–30 000)
**FG‐16**	7.6 ± 0.07	25.1 (18–35)a)
**FG‐17**	6.1 ± 0.08	823 (540–1200)
**FG‐18**	4.70 ± 0.15	19 500 (9500–40 100)
8‐OH‐DPAT	9.59 ± 0.12	0.25 (0.097–0.66)

(a) **FG‐4** and **FG‐5** vs. **FG‐6,** **FG‐16** vs. **FG‐13** (*p* < 0.01)**; FG‐7** vs. **FG‐8** and **FG‐9**, **FG‐10** vs. **FG‐12** (*p* < 0.001); **FG‐10** vs. **FG‐11** (*p* < 0.05).

**Table 2:** Binding affinity values of compounds **FG 1‐18** for 5‐HT_2A_ and 5‐HT_2C_ receptors.

5‐HT_2_ receptor binding affinity				
Compd	5‐HT_2A_ pKi	5‐HT_2A_ Ki (95% CI, nM)	5‐HT_2C_ pKi	5‐HT_2C_ Ki (95% CI, nM)
**FG‐1**	5.50 ± 0.08	3130 (1900–5300)	5.16 ± 0.24	6859 (2390–19,680)
**FG‐2**	5.53 ± 0.09	2900 (1700–5100)	6.08 ± 0.25	816 (262–2539)a)
**FG‐3**	5.20 ± 0.08	6300 (5400–10,900)	5.84 ± 0.37	1458 (250–8499)a)
**FG‐4**	5.33 ± 0.09	4700 (3000–7300)	5.14 ± 0.32	7279 (1654–32,040)
**FG‐5**	5.35 ± 0.1	4430 (1637–5082)	4.9 ± 0.31	12,480 (2691–57,880)
**FG‐6**	5.34 ± 0.16	4600 (2100–9980)	5.65 ± 0.15	2213 (1039–4715)a)
**FG‐7**	5.54 ± 0.13	2860 (1640–5100)a)	4.1 ± 0.33	80,750 (15,890–410,400)
**FG‐8**	5.94 ± 0.18	1100 (590–2200)a)	7.33 ± 0.37	46 (7.8–271)a)
**FG‐9**	4.85 ± 0.14	14,100 (7140–30,300)	5.7 ± 0.27	2029 (546–7545)
**FG‐10**	5.42 ± 0.15	3840 (2050–7740)	5.49 ± 0.48	3233 (426–2452)
**FG‐11**	5.83 ± 0.11	1480 (870–2600)a)	4.4 ± 0.23	40,500 (13,540–121,200)
**FG‐12**	5.16 ± 0.15	6930 (3580–15,500)	5.64 ± 0.29	286 (72–1138)a)
**FG‐13**	5.45 ± 0.08	3540 (2390–5260)	6.1 ± 0.15	817 (62–1078)a)
**FG‐14**	6.4 ± 0.1	430 (260–710)a)	7.14 ± 0.40	72 (21–250)a)
**FG‐15**	5.35 ± 0.15	3540 (2200–9160)	5.62 ± 0.27	2363 (635–8792)
**FG‐16**	5.9 ± 0.18	1370 (590–3200)	5.3 ± 0.22	4828 (1667–13,980)
**FG‐17**	5.2 ± 0.18	6060 (2500–14,600)	5.78 ± 0.22	1662 (593–4657)
**FG‐18**	5.56 ± 0.13	2730 (1420–5250)	7.8 ± 0.22	17 (5–57)a)
Ketanserin	8.64 ± 0.07	2.2 (1.0–3.0)	–	–
RS‐102 221	–	–	8.24 ± 0.23	5.7 (1.89–17.4)

a) 5‐HT_2A_: **FG‐7** and **FG‐8** vs. **FG‐9, FG‐13** vs. **FG‐14** (*p* < 0.01); **FG‐11** vs. **FG‐10** and **FG‐12; FG‐14** vs. **FG‐17.** 5‐HT_2C_: **FG‐3** vs. **FG‐1**, **FG‐6** vs. **FG‐5** (*p* < 0.05); **FG‐2** vs. **FG‐1** (*p* < 0.01); **FG‐8** vs. **FG‐7** and **FG‐9**; **FG‐12** vs. **FG‐10** and **FG‐11**, **FG‐14** vs. **FG‐13** and **FG‐15**, **FG‐18** vs. **FG‐16** and **FG‐17** (*p* < 0.001). **FG‐13** vs. **FG‐1** and **FG‐4** (*p* < 0.05); **FG‐8** vs. **FG‐2** and **FG‐5** and **FG‐11** and **FG‐17**, **FG‐18** vs. **FG‐3** and **FG‐6** and **FG‐9** and **FG‐12** and **FG‐16** and **FG‐18** (*p* < 0.001). **FG‐13** vs. **FG‐7**, **FG‐18** vs. **FG‐17**.

### 2.4 Functional Activation of the 5‐HT_1A_ Receptors

Potencies (EC_50_) and efficacies (E_max_) of 5‐HT_1A_ receptor activation by the series of **FG 1‐18** compounds are reported in **Table** **3**. As evidenced by the [^35^S] GTPγS functional assay, most screened compounds were agonists for the 5‐HT_1A_ receptor with potencies in the sub micromolar and micromolar range. The potencies of the compounds differed depending on the nature of the heterocyclic core linked through a propylene bridge to the arylpiperazine residue, but did not depend too much on the different position of the 4,5‐dihydrothiazole substituent on the phenyl ring of the arylpiperazine residue. As revealed by one‐way ANOVA, the choice of the endo‐N‐hydroxy‐5‐norbornene‐2,3‐dicarboximide (**FG‐7** and **FG‐8**) and 5‐norbornene‐2‐carboxamide (**FG‐14**, **FG‐16**, **FG‐17,** and **FG‐18**) scaffolds increased the potency of 5‐HT_1A_ receptor activation. The EC_50_ values for the above‐mentioned derivatives were in the micromolar range, i.e., 3.01, 7.9, 23.3, 21.1 and 8.8 μM for **FG‐7**, **FG‐8**, **FG‐14**, **FG‐17,** and **FG‐18**, respectively. In addition, the potency of the isonicotinic derivative **FG‐6** is also interesting, with an EC_50_ value of 9.4 μM. Therefore, **FG‐7** is one of the most potent compounds, with EC_50_ of 3.01 μM. Furthermore, **FG‐6**, **FG‐7**, and **FG‐8** show higher or equal efficacy than the reference compound, 8‐hydroxy‐dipropylaminotetraline (8‐OH‐DPAT), classifying them as full agonists. Regarding the exo (**FG 13‐15**) and endo (**FG 16‐18**) isomer pairs, they did not differ in terms of efficacy while the endo isomers showed higher potency than the exo with the **FG‐18** derivative showing the best profile in terms of efficacy and potency. In contrast, combining the other heterocyclic nuclei under investigation with their respective arylpiperazines yielded compounds with a poorer 5‐HT_1A_ receptor activation profile in terms of potency, although most conserved their efficacy at activating 5‐HT_1A_ receptor. Finally, the study also identified some inactive derivatives (**FG‐3**, **FG‐9** and **FG‐11**) that do not express any agonist activity for the 5‐HT_1A_ receptor.

**Table 3:** Agonist activity of compounds for the 5‐HT_1A_ receptor.

5‐HT_1A_ receptor G‐protein stimulation			
Compd	pEC_50_	EC_50_ (95% CI, μM)	E_max_ (%) ± SEM
**FG‐1**	4.7 ± 0.3	16.5 (3.9–69.1)a)	138 ± 11.5
**FG‐2**	3.9 ± 0.8	125 (2.4–643)	136 ± 10.2
**FG‐3**	No activity	No activity	No activity
**FG‐4**	4.3 ± 0.2	5.3 (2.1–135)	132 ± 14.9
**FG‐5**	4.7 ± 0.13	18.2 (9.9–33.3)	185 ± 17.6a)
**FG‐6**	5.0 ± 0.11	9.4 (4.8–18.6)	184 ± 11.0a)
**FG‐7**	5.5 ± 0.07	3.01 (2.2–4.2)	177 ± 3.3
**FG‐8**	5.1 ± 0.08	7.9 (5.3–11.6)	184 ± 6.6
**FG‐9**	No activity	No activity	No activity
**FG‐10**	4.3 ± 0.5	52 (3.6–76)	120 ± 14.9
**FG‐11**	No activity	No activity	No activity
**FG‐12**	4.3 ± 0.64	46.2 (2.1–76)	135 ± 18.2
**FG‐13**	4.1 ± 0.58	79.6 (0.5–132)	132 ± 8.4
**FG‐14**	4.6 ± 0.4	23.3 (2.7–202)	133 ± 5.7
**FG‐15**	4.05 ± 0.8	88.1 (20–380)	137 ± 4.7
**FG‐16**	4.4 ± 0.35	41 (7.8–213)	127 ± 3.2
**FG‐17**	4.7 ± 0.3	21.1 (5.9–75.4)	134 ± 2.5
**FG‐18**	5.05 ± 0.23	8.8 (3.0–26)	144 ± 8.2
8‐OH‐DPAT	7.4 ± 0.04	0.038 (0.031–0.045)	177 ± 1.2
WAY 100 635	–	–	–

a) **EC**_**50**_ **FG‐1** vs. **FG‐2** and **FG‐3**.

### 2.5 In Vitro Evaluation of 5‐HT‐Evoked Contractions

Successively, the compounds **FG‐2**, **FG‐7**, **FG‐8**, **FG‐11**, **FG‐14**, **FG‐16**, **FG‐17**, and **FG‐18** with better affinity/selectivity binding profiles toward 5‐HT_2A_ receptors were tested by in vitro assay to determine their activity on 5‐HT‐evoked contractions. In the rat ileum, 5‐HT_2A_ receptors are located on smooth muscles, and their activation by 5‐HT is known to introduce contraction. Therefore, serotonin‐induced contractions of the rat ileum are depressed by 5‐HT_2A_ receptor antagonists, and it is important to note that the 5‐HT_2__A_ receptor is involved in the contraction of the longitudinal smooth muscle in rat.^[^ ^16^ ^]^ The tested compounds were compared with ketanserin, a selective 5‐HT_2A_ receptor antagonist. Ketanserin has an IC_50_ of 6.44 × 10^−10^ M (4.50 × 10^−11^–4.46 × 10^−9^ M). Among the tested compounds, only **FG‐8** exhibited a good IC_50_. The other compounds inhibited serotonin‐evoked contractions, but they have higher IC_50_ values compared to **FG‐8**. Collectively, the results show the potency (expressed by IC_50_ value) and the efficacy (expressed by E_MAX_ value) of the compounds under investigation in inhibiting 5‐HT‐induced contractions in the rat ileum. Considering the E_MAX_ value the order of efficacy was **FG‐16** (39.09%) > **FG‐18** (32.80%) > **FG‐11** (28.70 %) > **FG‐2** (25.41%) > **FG‐17** (25.03%) > **FG‐7** (24.46%) > **FG‐14** (22.48 %) > **FG‐8** (21.35%). On the other hand, the IC_50_ values order was: **FG‐8** (1.55 × 10^−7^ M) > **FG‐17** (1.25 × 10^−6^ M) > **FG‐7** (1.79 × 10^−6^ M) > **FG‐14** (2.59 × 10^−6^ M) > **FG‐18** (2.97 × 10^−6^ M) > **FG‐11** (4.39 × 10^−6^ M) > **FG‐2** (6.12 × 10^−6^ M) > **FG‐16** (6.35 × 10^−6^ M).

### 2.6 In Vivo Behavioral Test

**Figure 3:**
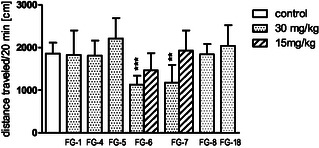
The influence of the tested compounds (30 and 15 mg/kg) on the spontaneous locomotor activity of mice. Investigated compounds were injected i.p. 60 min before the test. Locomotor activity was measured for 20 min. Data are expressed as mean ± SEM values of the 1 independent experiment; ***p < 0.001, **p < 0.01 versus control (Dunnett's post hoc test). One‐way ANOVA showed significant changes in locomotor activity of mice after administration of the tested compounds at doses 30 and 15 mg kg^−1^ (F (9,92) = 7836; *p* < 0.0001). Dunnett's post hoc test confirmed a significant decrease in locomotor activity of mice after the administration of compound **FG‐6** and **FG‐7** at the dose of 30 mg kg^−1^, respectively (*p *< 0.001) and (*p *< 0.01) during 20 min of observation.

Compounds **FG‐1**, **FG‐4**, **FG‐5**, **FG‐6**, **FG‐7**, **FG‐8**, and **FG‐18** were selected for further functional in vivo studies. The first part of the experiments included motor coordination tests and locomotor activity tests that are generally accepted as basic tests in central activity investigations of new agents.^[^ ^17^ ^]^ Firstly, all compounds were tested at the dose of 30 mg kg^−1^ in tests assessing animal coordination (the rota‐rod and chimney test) and none of the compounds induced significant coordination disorders. The same dose, i.e., 30 mg/kg was used in motility test (**Figure** **3**), and we noted a significant decrease in spontaneous locomotor activity after administration of **FG‐6** (*p* < 0.001) and **FG‐7** (*p *< 0.01) during the 20‐minutes observation period. These compounds (**FG‐6** and **FG‐7**) administered at half the original dose (15 mg kg^−1^) did not change the motility of mice.

**Figure 4:**
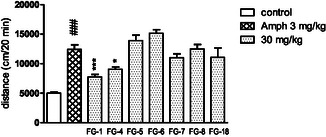
The influence of the tested compounds (30 mg kg^−1^) on amphetamine‐induced hyperactivity in mice. Investigated compounds were injected i.p. 60 min before the test and amphetamine (amph, 3 mg kg^−1^) was administered s.c. 30 min after the compounds. The results are expressed as mean ± SEM of the 1 independent experiment. ### *p *< 0.001 versus control, *** *p *< 0.001, * *p *< 0.05, versus amph 3 mg kg^−1^ (Dunnett's post hoc test). One‐way ANOVA showed significant changes in locomotor activity of mice after administration of the tested compounds at dose 30 mg kg^−1^ with the amphetamine (amph, 3 mg kg^−1^) (F(8,67) = 16,75; *p* < 0.0001). Dunnett's post hoc test confirmed a significant decrease in amphetamine‐induced hyperactivity of mice after the administration of compounds **FG‐1** (*p* < 0.001) and **FG‐4** (*p* < 0.04) at the dose of 30 mg/kg during 20 min of observation.

**Figure 5:**
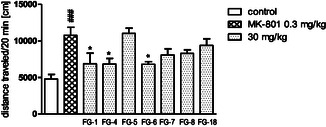
The influence of the tested compounds (30 mg kg^−1^) on MK‐801‐induced hyperactivity in mice. Investigated compounds were injected i.p. 60 min before the test, and MK‐801 (0.3 mg kg^−1^) was administered s.c. 30 min after the compounds. The results are expressed as mean ± SEM of the 1 independent experiment. ### *p* < 0.001 versus control, * *p* < 0.05, versus MK‐801 0.3 mg kg^−1^ (Dunnett's post hoc test). One‐way ANOVA showed significant changes in locomotor activity of mice after administration of the tested compounds at dose 30 mg kg^−1^ with the MK‐801 (0.3 mg kg^−1^) (F (8,54) = 5351; *p *< 0.0001). Dunnett's post hoc test confirmed a significant decrease in MK‐801‐induced hyperactivity of mice after the administration of compounds **FG‐1**, **FG‐4**, and **FG‐6** (*p *< 0.05) at the dose of 30 mg/kg during 20 min of observation.

However, it was noted that hyperactivity induced by amphetamine (**Figure** **4**), and MK‐801 (**Figure** **5**) was significantly reduced by **FG‐1** and **FG‐4** and that hyperactivity induced by MK‐801 was additionally reduced by compound **FG‐6**.

**Figure 6:**
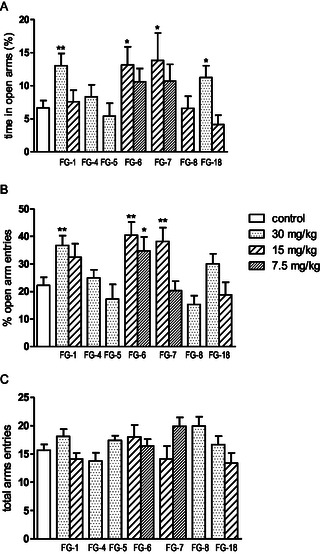
A) The influence of the investigated compounds on elevated plus‐maze performance (EPM) in mice—percentage of time spent in open arms, B) percentage of the open arm entries, C) total arm entries [**C**]. Investigated compound was injected i.p. 60 min before the test. The results are expressed as mean ± SEM of the 1 independent experiment. **p < 0.01, *p < 0.05 vs control (student's *t* test) (Dunnett's post hoc test). Statistical analysis exerted significant influence of acute administration of tested compounds on the behavior of mice in the EPM. One‐way ANOVA showed significant changes in percentage of time spent in open arms of EPM A) (F (11,104) = 2387; *p *< 0.05 and in the percentage of open arm entries B) (F (11,104) = 4504; *p *< 0.0001). There were no significant changes in total arm entries C) (F (10,81) =1.564; *p *> 0.05). Dunnett's post hoc test confirmed a significant increase in time spent in open arms after the administration of compounds **FG‐1** and **FG‐18** at the dose of 30 mg kg^−1^, *p *< 0.01 and *p *< 0.05, respectively, and **FG‐6**, **FG‐7** at the dose of 15 mg kg^−1^ (*p *< 0.05). Dunnett's post hoc test confirmed also the increase in the percentage of open arm entries for compounds **FG‐1** (30 mg kg^−1^, *p* < 0.01), **FG‐6** (15 mg kg^−1^ *p *< 0.01 and 7.5 mg kg^−1^ *p *< 0.05), and **FG‐7** at the dose of 30 mg kg^−1^ (*p *< 0.05).

This activity may reflect antipsychotic properties of these new compounds, since animal models of schizophrenia, commonly employed for preclinical studies of antipsychotic properties of drugs, regard mainly amphetamine and MK‐801 models.^[^ ^18^ ^]^ The first model is based on the manipulation of the dopaminergic system activity, and it may primarily respond to drugs that affect this neurotransmitter system. Many neuroleptics acting as dopaminergic antagonists reverse this effect.^[^ ^18^ ^]^ On the other hand, several preclinical tests have pointed to the role of 5‐HT_2C_ ligands in the modulation of monoaminergic systems, including dopaminergic. Indeed, dysfunction in serotoninergic activity could contribute to the alteration of dopaminergic function seen in schizophrenia.^[^ ^19^ ^]^ Furthermore, due to the modulation of the central serotonin neurotransmission, the new compounds may also show anxiolytic and/or antidepressant activity.^[^ ^20^, ^21^ ^]^ Considering this premise, as well as in vitro data obtained for the compounds (mixed 5‐HT_1A_/5‐HT_2_ affinity profile for the compounds), we examined their antidepressant and anxiolytic potential in behavioral models commonly used in mice, i.e., FST and EPM test. Anxiety and stress‐related disorders are serious mental health problems that affect daily functioning and cause significant costs to public health. Charles Darwin's early observation that both animals and humans have similar emotional expressions paved the way for the study of the mechanisms of mental disorders in other mammals, especially rodents. The anxiolytic effects of the new compounds were studied using the EPM (**Figure** **6**).

**Figure 7:**
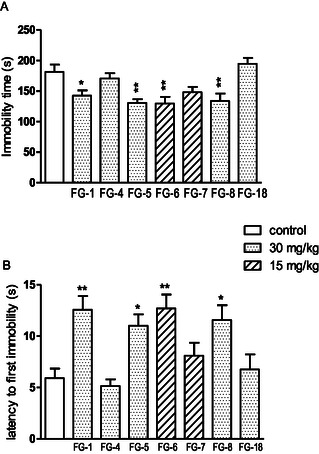
The influence of tested compounds A) (at the higher doses of 30 and 15 mg kg^−1^) on the total duration of immobility B) and latency to first immobility in the FST in mice. The investigated compounds were administered i.p. 60 min before the test. The values represent means ± SEM of the 1 independent experiment. **p < 0.01, *p < 0.05 vs control (Dunnett's post hoc test). One‐way ANOVA showed the statistically significant influence of acute administration of tested compounds A) (at higher doses 30 or 15 mg kg^−1^) on the immobility of mice in the FST: F (7,63) = 6179; *p* < 0.0001 B) and latency to first immobility: F (7,63) = 6171; *p* < 0.0001. Dunnett's post hoc test confirmed a significant decrease in immobility of mice after the administration of compound **FG‐1** (30 mg kg^−1^; *p *< 0.05), **FG‐5** and **FG‐8** (30 mg; *p *< 0.01) and **FG‐6** (15 mg kg^−1^: *p *< 0.01) and a significant increase in latency to first immobility for **FG‐1** (30 mg kg^−1^; *p *< 0.01), **FG‐5** and **FG‐8** (30 mg; *p* < 0.05) and **FG‐6** (15 mg kg^−1^: *p *< 0.01).

**Figure 8:**
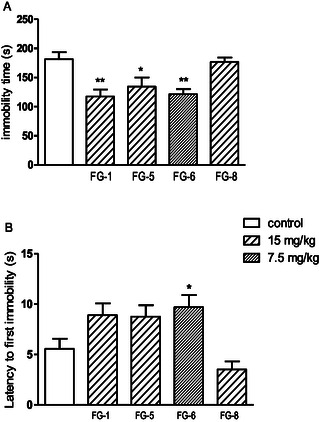
The influence of tested compounds (at the lower doses of 15 and 7.5 mg kg^−1^) on the total duration of immobility A) and latency to first immobility B) in the forced swim test (FST) in mice. The investigated compounds were administered i.p. 60 min before the test. The values represent means ± SEM of the 1 independent experiment. **p < 0.01, *p < 0.05 vs control (Dunnett's post hoc test). One‐way ANOVA showed the statistically significant influence of acute administration of tested compounds (at lower doses 15 or 7.5 mg kg^−1^) on the immobility of mice in the FST: F (4,39) = 7093; *p* < 0.001 (A) and latency to first immobility: F (4,39) = 5609; *p *< 0.001). Dunnett's post hoc test confirmed a significant decrease in immobility of mice after the administration of compound **FG‐1** (15 mg kg^−1^; *p *< 0.01), **FG‐5** (15 mg kg^−1^; *p *< 0.05) and **FG‐6** (7.5 mg kg^−1^; *p *< 0.01) and a significant increase in latency to first immobility for **FG‐6** (7.5 mg kg^−1^: *p *< 0.05).

This test exploits the natural tendency of mice to prefer closed, dark spaces and their fear of open spaces. A reduction in the time spent in the closed arms of the maze is considered an indicator of reduced anxiety in mice. The EPM test is considered an effective method for assessing anxiety‐related behaviors and evaluating the therapeutic potential of new drugs.^[^ ^22^ ^]^ Among others, buspirone, an aryl piperazine derivative similar to the compounds tested in this study, proved active in this test. It has been confirmed as a partial agonist of 5‐HT_1A_ serotonin receptors and affects serotonin transmission in limbic structures of the brain. Buspirone was shown to be effective in treating generalized anxiety disorder, although it is less effective in relieving panic.^[^ ^23^ ^]^ It represents a well‐tolerated drug with few side effects and an alternative to benzodiazepines, whose frequent use may lead to the development of tolerance.^[^ ^24^ ^]^ The results obtained from the experiments carried out in this study showed that compounds **FG‐1** and **FG‐18** (30 mg kg^−1^), **FG‐6** (30 and 15 mg kg^−1^) and **FG‐7** (15 mg kg^−1^) were active in the EPM test, which significantly increased the time spent in open arms and increased the number of open arm entries. It may be assumed that its anxiolytic effect, as in the case of buspirone, due to its affinity, may result from the interaction with the 5‐HT_1A_ receptors. The next step was to test the antidepressant effect of the new compounds. We used FST (**Figure** **7**), which is a simple and fast test established in experimental pharmacology for detecting the antidepressant effect of tested substances.^[^ ^25^ ^]^ The animals were placed in the water and the depressive behavior was observed as immobility and floating of the rodent in the water with only slight movements. In this test, drugs with an antidepressant effect shorten the time of immobility and extend the latency to first immobility, and those with depressive properties act oppositely.^[^ ^26^ ^]^ It is designed to reflect human depression. Selective serotonin reuptake inhibitors are an example of antidepressants active in this test. Due to their effectiveness, they are one of the most commonly used drugs in depression. After administration of tested compounds **FG‐1**, **FG‐5**, **FG‐8** (at dose of 30 mg kg^−1^) and **FG‐6** (15 mg kg^−1^), mice remained motionless for a shorter time compared to the control group, and the time to the first immobility has increased which indicates antidepressant‐like activity of these compounds. Three of them, **FG‐1**, **FG‐5**, and **FG‐6** were also active in a dose twice as small, e.g., 15 and 7.5 mg kg^−1^, respectively (**Figure** **8**).

**Figure 9:**
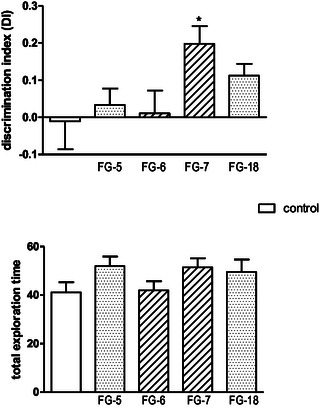
The influence of tested compounds (at the doses of 30 and 15 mg kg^−1^) on the performance of mice in the novel object recognition (NOR) test and total exploration time. The investigated compounds were administered i.p. 60 min prior to testing in the first trial. The data are expressed as mean ± SEM values. *p < 0.05 versus control group (Dunnett's post hoc test).

Mental illnesses such as depression are often associated with impaired cognitive functions, learning and memory. Also in this context, these new derivatives seem to be good candidates. It has been shown, inter alia, that the well‐known antidepressant vortioxetine, also characterized by the arylpiperazine structure, has an antidepressant and anxiolytic effect, and is also pro‐cognitive.^[^ ^27^ ^]^ In this study, it was therefore decided to assess the pro‐cognitive properties of the new compounds. Only **FG‐7** showed activity in the novel object recognition (NOR) test (**Figure** **9**), supporting memory consolidation, expressed by the discrimination index (DI).

**Figure 10:**
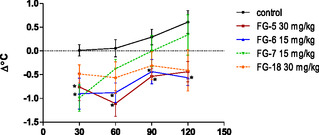
The influence of tested compounds (at doses of 30 and 15 mg kg^−1^) on the mice body temperature. Body temperature was measured over a total period of 120 min after the tested compound injection. Data are expressed as mean ± SEM values. *p < 0.05 versus control (Dunnett's post hoc test). Data analysis showed the statistically significant influence of tested compounds (at doses 30 and 15 mg kg^−1^) on the body temperature in normothermic mice: two‐way ANOVA revealed statistically significant effects of the compound F (4160) = 8.57, *p* < 0.0001). There was no significant effect for time F (3160) =4.95, *p* = 0.0026) as well as compound × time (F 12,160) = 0.69, *p* = 0.7571).

The results of measurement of the body temperature in normothermic mice may also confirm the correlation between the serotonergic system and mechanism of action of studied compounds (**Figure** **10**). According to literature data, the system involved in the body temperature regulation is in hypothalamus.^[^ ^28^ ^]^ 5‐HT_2_ receptor agonists (e.g., DOI) and 5‐HT_1A_ antagonists can induce hyperthermia, whereas 5‐HT_2_ antagonists (e.g., 8‐OH‐DPAT, Ketanserine) or 5‐HT_1A_ agonists cause hypothermia.^[^ ^29^ ^]^ Bonferroni's post hoc test revealed a significant decrease in the body temperature of mice after the administration of the compounds **FG‐5** (30 mg kg^−1^) and **FG‐6** (15 mg kg^−1^) from 30 to 90 min (*p* < 0.05), and after intake of the compound **FG‐6** also in 120 min (*p* < 0.05).

**Figure 11:**
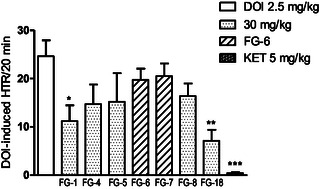
The influence of tested compounds (at the doses of 30 and 15 mg kg^−1^) on DOI‐induced HTR in mice. The test was performed immediately after DOI and 60 min after administration of the tested compounds. The data are expressed as mean ± SEM values. ***p < 0.001, **p < 0.01, *p < 0.05 versus control group (Dunnett's post hoc test). One‐way ANOVA showed the statistically significant influence of acute administration of tested compounds (at doses 30 and 15 mg kg^−1^) on the HTR in mice: F (8,81) = 4,983; *p *< 0.001. Dunnett's post hoc test confirmed a significant decrease in HTR of mice after the administration of compound **FG‐1** (30 mg kg^−1^; *p* < 0.05) and **FG‐18** (30 mg kg^−1^; *p* < 0.01).

Other tested compounds did not affect body temperature in a clear, statistically significant manner, as compared to the control. The results of body temperature measurement allow for conclusion that the action of **FG‐5** and **FG‐6** could be an effect of a stimulation of 5‐HT_1A_ receptors. The other feature of some tested derivatives was also the ability to diminish the number of DOI‐induced head twitch reactions (HTR) in mice. In this test (**Figure** **11**) compounds **FG‐1** and **FG‐18** were active, although a similar trend can be seen in the case of other compounds, but the results obtained were not statistically significant. This activity probably reflects antagonistic properties towards 5‐HT_2A_ receptors.

### 2.7 Zebrafish Experiments

**Figure 12:**
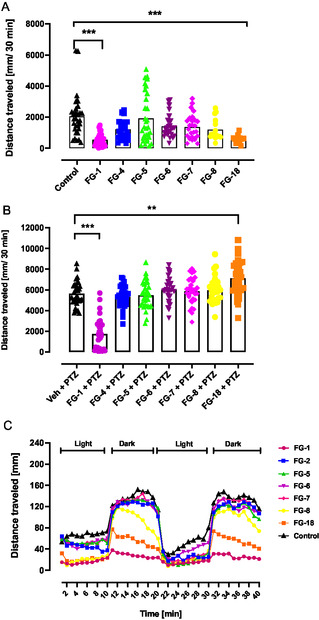
The effect of arylpiperazine derivatives **FG‐1** (500 μM), **FG‐2** (250 μM), **FG‐5** (400 μM), **FG‐6** (100 μM), **FG‐7** (100 μM), **FG‐8** (500 μM), and **FG‐18** (25 μM) on: A) basic locomotor activity, B) PTZ‐induced (20 mM) seizure‐like behavior, and C) behavior in the light–dark transition assay at 5 dpf larval zebrafish. Larval zebrafish were exposed to arylpiperazine derivatives at 4 dpf for 20 h, after which respective analysis took place. The data are shown as total distance traveled (panels **A** and **B**) or distance traveled within 2‐min long time bins (panel **C**). Data were analyzed using Kruskal–Wallis or Friedman test. Data are shown as individual values (panels **A** and **B**) or median only (panel **C**). **p < 0.01, ***p < 0.001 versus respective control groups. Number of larvae: A) *n* = 16–32 per group, B) *n* = 30–34 per group, C) *n* = 40 per group. PTZ—pentylentetrazole; veh—vehicle.

In addition to the in vivo tests conducted on mice, further studies were performed using zebrafish as an alternative vertebrate model. Our study revealed that new arylpiperazine derivatives influenced basic locomotor activity of zebrafish larvae (*p *< 0.001; Kruskal–Wallis statistic 58.57). Both **FG‐1** and **FG‐18** decreased (*p *< 0.001) larval zebrafish basic locomotor activity which may suggest their sedative effects.^[^ ^30^ ^]^ Additionally, **FG‐4**, **FG‐6**, **FG‐7**, and **FG‐8** slightly decreased larval activity, although results did not reach statistical significance (**Figure** **12**A). Subsequently, the effect of arylpiperazine derivatives via acute seizure assay in larval zebrafish was examined. Seizures, induced by acute application of pentylentetrazole (PTZ), manifests in the form of increased locomotor activity, and compounds with antiseizure potential decrease distance traveled compared to only PTZ‐treated larvae.^[^ ^31^, ^32^, ^33^ ^]^ Here, we revealed that **FG‐1** substantially decreased (*p *< 0.001) distance traveled by larvae exposed to an acute dose of PTZ (*p* < 0.001; Kruskal–Wallis statistic 97.46). In contrast, **FG‐18** significantly increased the distance traveled by PTZ‐exposed larvae (*p *< 0.01; Figure 12B), suggesting that, unlike **FG‐1** which may possess anticonvulsant properties, **FG‐18** may instead exacerbate convulsions.

Finally, in the light‐dark transition assay, Friedman test revealed that arylpiperazine derivatives influenced larval behavior (*p *< 0.001; 227.1) (Figure 12C). Suddenly turning off the light induced in larval zebrafish an abrupt increase of activity, which is interpreted as anxiety‐like behavior and compounds which attenuate this reflex are considered to exert anxiolytic activity.^[^ ^34^, ^35^ ^]^ In our scenario, only **FG‐1** (*p *< 0.001) and **FG‐18** (*p *< 0.001) influenced larval behavior under the dark conditions, therefore, they may have anxiolytic activity. The remaining substances did not affect the larvae's behavior compared to control larvae.

### 2.8 Molecular Docking Studies

**Figure 13:**
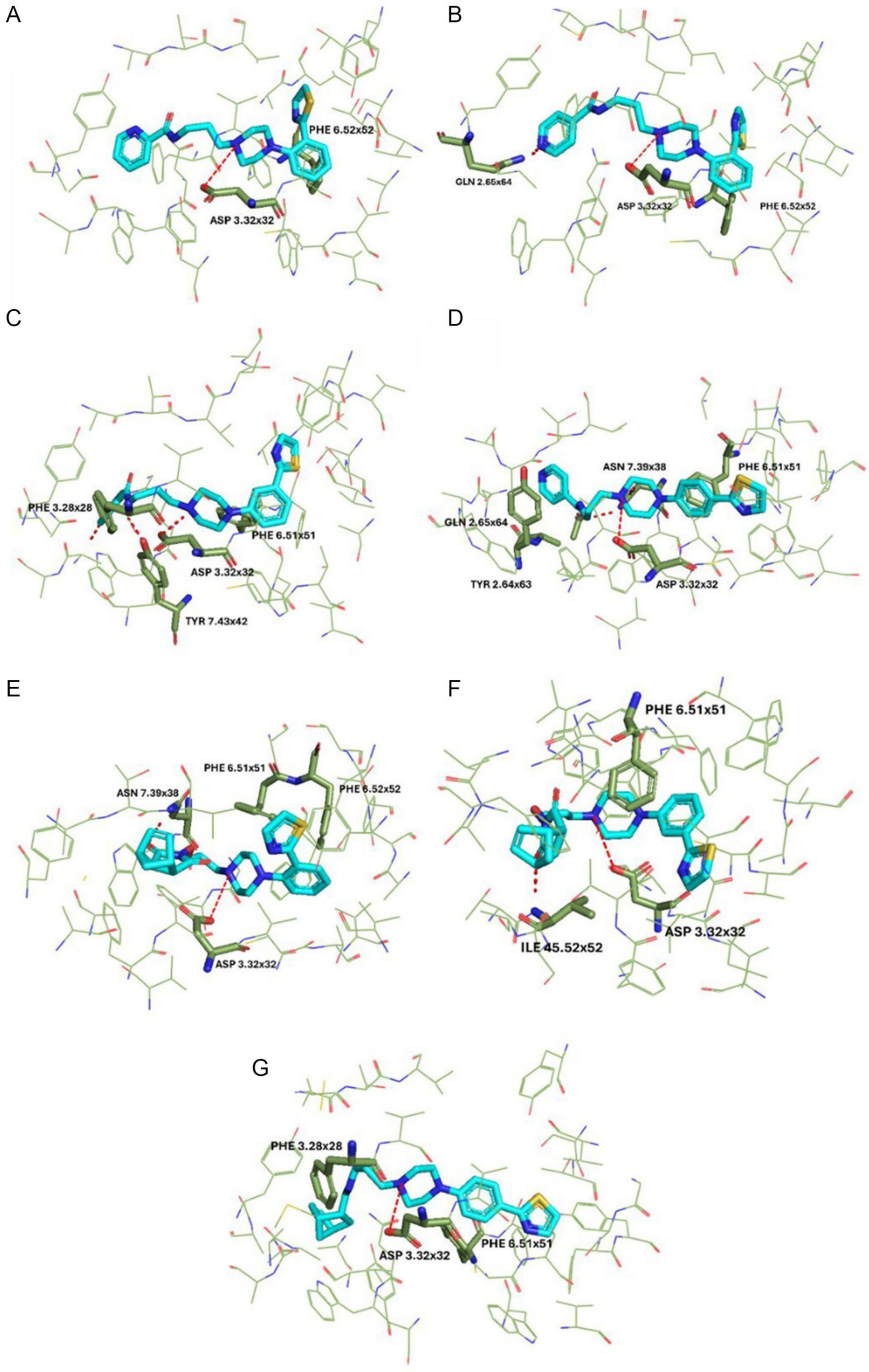
Selected ligands in complex with serotonin 5‐HT_1A_ receptor: A) **FG‐1**, B) **FG‐4**, C) **FG‐5**, D) **FG‐6**, E) **FG‐7**, F) **FG‐8**, and G) **FG‐18**. Protein shown in wire representation with green carbon atoms. The most important residues shown as sticks. Ligands shown as sticks with light‐blue carbon atoms. Polar interactions shown as red dashed lines. All hydrogen atoms of the receptor and nonpolar hydrogen atoms of the ligands omitted for clarity.

**Figure 14:**
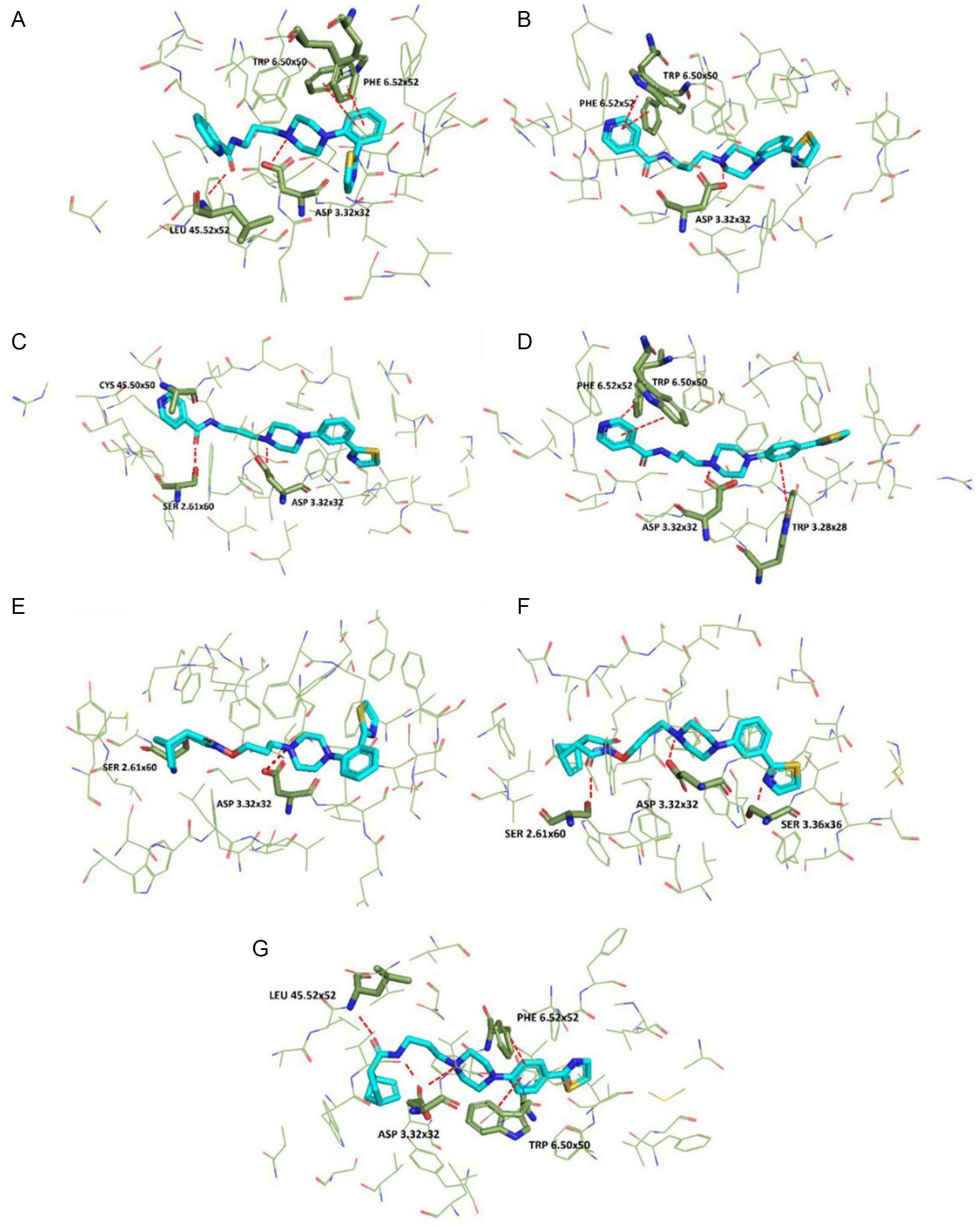
Selected ligands in complex with serotonin 5‐HT_2A_ receptor: A) **FG‐1**, B) **FG‐4**, C) **FG‐5**, D) **FG‐6**, E) **FG‐7**, F) **FG‐8**, and G) **FG‐18**. Protein shown in wire representation with green carbon atoms. The most important residues shown as sticks. Ligands shown as sticks with light‐blue carbon atoms. Polar interactions shown as red dashed lines. All hydrogen atoms of the receptor and nonpolar hydrogen atoms of the ligands omitted for clarity.

**Figure 15:**
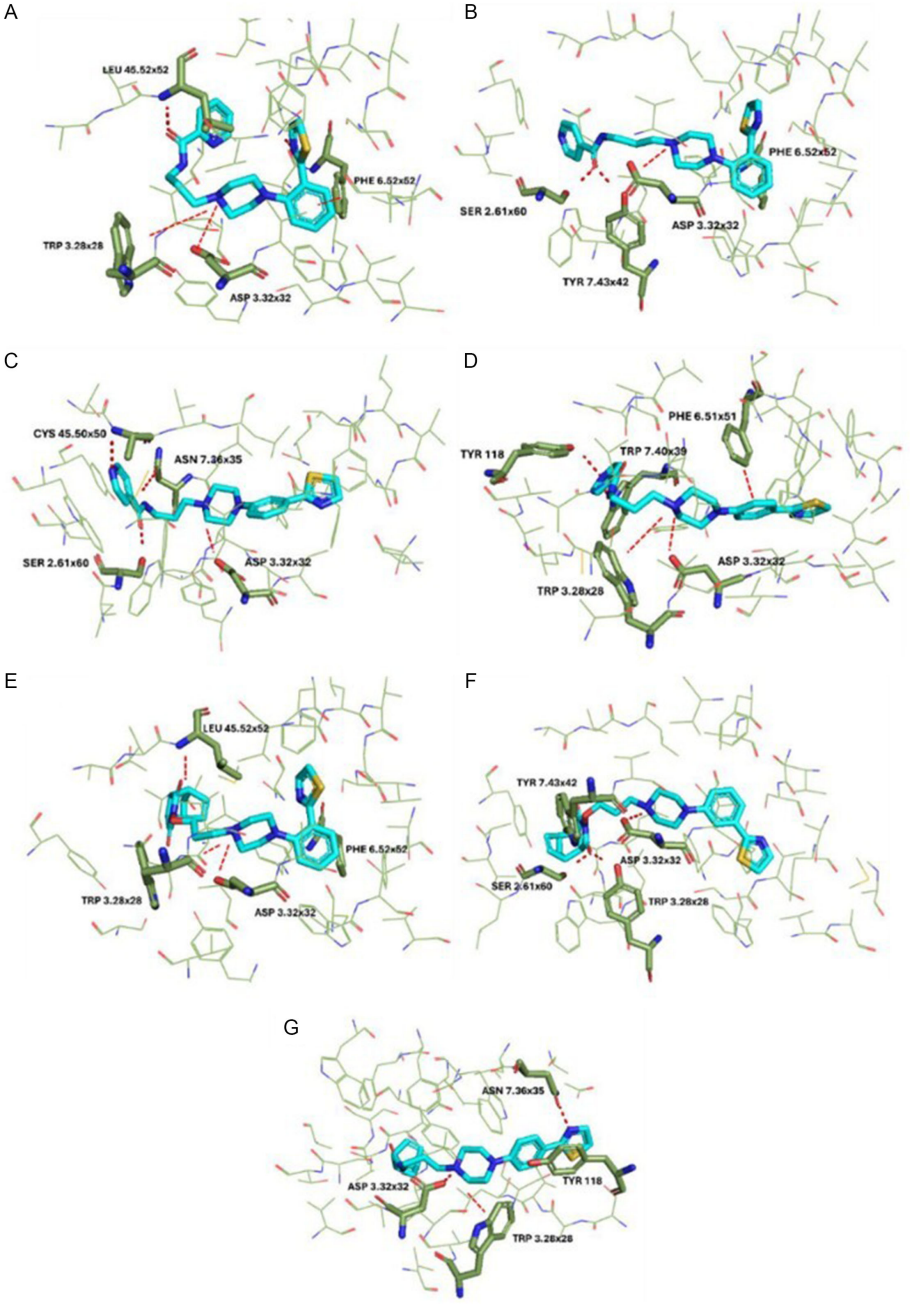
Selected ligands in complex with serotonin 5‐HT_2C_ receptor: A) **FG‐1**, B) **FG‐4**, C) **FG‐5**, D) **FG‐6**, E) **FG‐7**, F) **FG‐8**, and G) **FG‐18**. Protein shown in wire representation with green carbon atoms. The most important residues shown as sticks. Ligands shown as sticks with light‐blue carbon atoms. Polar interactions shown as red dashed lines. All hydrogen atoms of the receptor and nonpolar hydrogen atoms of the ligands omitted for clarity.

Molecular docking was performed to further investigate the ligand–receptor interactions of the reported compounds. Among all those synthesized, seven compounds were selected after careful evaluations arising from the results from the in vivo assays, and a detailed description of the molecular interactions was conducted for them. The above compounds, **FG‐1**, **FG‐4**, **FG‐5**, **FG‐6**, **FG‐7**, **FG‐8**, and **FG‐18**, were docked on the serotonergic receptor subtypes 5‐HT_1A_, 5‐HT_2A_, and 5‐HT_2C_ models as shown in **Figure** **11**, **12**, **15**. The compounds under investigation, follow the classical pharmacophore model for the aminergic G protein‐coupled receptor (GPCRs) ligands.^[^ ^36^ ^]^ In medicinal chemistry, this model is well known and is essential to be successful in understanding the interactions of potential ligands with specific receptors; in this case, the presence of an electrostatic interaction between the protonatable nitrogen atom (represented in all the synthesized compounds by piperazine nitrogen) and the conserved Asp3.32 of the receptor is essential. This residue is located in the third transmembrane helix TM3 and mediates an essential salt bridge; this is a key interaction, shown in the figures, and is recurring in all complexes.^[^ ^37^ ^]^ As presented, the interaction with Phe6.52 is recurrent in the considered ligands.^[^ ^38^ ^]^ This residue is involved in π–π stacking interactions with the N‐aryl piperazine moiety. Thus, certainly Asp3.32 and Trp6.48 are required for interaction with the arylpiperazine group, as well as aromatic residues such as Phe 6.52 are essential when the selectivity of serotonergic receptor subtypes is to be investigated. Considering the 5‐HT_2C_ receptor, interactions with Asn7.35 and Ser2.60 are common as shown in Figure 15 for compounds FG**‐4**, **FG‐5**, **FG‐8**, and **FG‐18**; previous studies show that ligands may form hydrogen bonds with this receptor subtype.^[^ ^39^ ^]^ In addition, both compounds **FG‐6** and **FG‐18** interact with Tyr118 from ECL1, the first extracellular loop. In contrast, regarding the 5‐HT_1A_ receptor, crucial is the interaction with Asn7.38, which is highlighted for compounds **FG‐6** and **FG‐7** in Figure 13; the interaction with Phe3.28 for compounds **FG‐5** and **FG‐18** and the interaction with Tyr7.42 showed in compound **FG‐5** were found to be essential in many ligand–receptor complexes. Finally, π–π stacking interaction with Phe6.52 is common in the 5‐HT_2A_ receptor subtype as demonstrated by compounds **FG‐1**, **FG‐4**, **FG‐6**, and **FG‐18** (Figure 14).

## 3 Experimental Section

### 3.1 General Information (Synthesis)

All reagents were commercial products purchased from Aldrich. Melting points were determined using a Buchi melting point M560 apparatus and are uncorrected. ^1^H NMR and ^13^C NMR spectra were obtained with a Bruker Avance NEO 400 MHz instrument (Bruker BioSpin Corporation, Billerica, MA, USA). Unless otherwise stated, all spectra were recorded in CDCl_3_. Chemical shifts are reported in ppm using Me_4_Si as internal standard. The following abbreviations are used to describe peak patterns when appropriate: s (singlet), d (doublet), t (triplet), m (multiplet), q (quartet), qt (quintet), dd (double doublet), ddd (double dd), and bs (broad singlet). Mass spectra of intermediates and final products were performed on LTQ Orbitrap XL Fourier transform mass spectrometer (FTMS) equipped with an ESI ION MAX (Thermo Fisher). Where analyses are indicated only by the symbols of the elements, and results obtained are within ±0.4% of the theoretical values. All reactions were followed by TLC, carried out on Merck silica gel 60 F254 plates with fluorescent indicator and the plates were visualized with UV light (254 nm). Preparative chromatographic purifications were performed using silica gel column (Kieselgel 60). Solutions were dried over Na_2_SO_4_ and concentrated with Buchi rotary evaporator at low pressure.

### 3.2 Synthetic Procedures

#### 3.2.1 Synthesis of N‐(3‐Chloropropyl) Picolinamide (B)

DCC (1 equiv.) and hydroxybenzotriazole, HOBt (1 equiv.) were added to a mixture of picolinic acid (2.00 g, 16 mmol) in acetonitrile (20 mL) and the reaction was stirred for 1 h at 0 °C. Then, TEA (1 equiv.) and 3‐chloropropan‐1 amine hydrochloride (1 equiv.) was added to the reaction mixture and the resulting solution was stirred for 24 h at room temperature. The mixture was then cooled to 0 °C to precipitate N,N’‐dicyclohexylurea (DCU) that was filtered under vacuum. The filtrate was evaporated and after dissolution in DCM, the residue was washed with NaHCO_3_, water and brine. The combined organic layers were dried on anhydrous Na_2_SO_4_ and concentrated in vacuo. The crude chloroalkylpicolinamide was purified by column chromatography (dichloromethane/methanol 9:1 v/v), yielding N‐(3‐chloropropyl) picolinamide (**B**).

#### 3.2.2 Synthesis of N‐(3‐Chloropropyl) Isonicotinamide (D)

DCC, (1.1 equiv.) and HOBt, (1.1 equiv.) were added to a mixture of isonicotinic acid (2.00 g, 16 mmol) in DMF (20 mL) and the reaction was stirred for 30 min. at 0 °C. Then, TEA (1.1 equiv.) and 3‐chloropropan‐1‐amine hydrochloride (1 equiv.) were added to the reaction mixture and the resulting solution was stirred for 24 h at room temperature. The mixture was then cooled to 0 °C, to precipitate DCU, and filtered. The filtrate was evaporated and after dissolution in DCM, the residue was washed with NaHCO_3_, water and brine. The combined organic layers were dried on anhydrous Na_2_SO_4_ and concentrated in vacuo. The crude chloroalkylisonicotinamide was purified by column chromatography (DCM/methanol 9:1 v/v), yielding N‐(2 chloroethyl) isonicotinamide.

#### 3.2.3 Synthesis of 2‐(3‐Chloropropoxy)‐3a,4,7,7a‐Tetrahydro‐1H‐4,7‐Methanoisoindole‐1,3(2H)‐Dione (F)

A solution of absolute ethanol (20 mL) and sodium hydroxide (1 equiv.) was reacted with commercially available endo‐N‐hydroxy‐5‐norbornene‐2,3‐dicarboximide (2.00 g, 11 mmol), and 1‐bromo‐3‐chloropropane (1 equiv.) at 70 °C for 24 h. Later, the mixture was cooled to room temperature. The residue was evaporated and after dissolution in DCM, was washed with water and brine. The combined organic layers were dried on anhydrous Na_2_SO_4_ and concentrated in vacuo. The crude product was purified by silica gel open chromatography using DCM/methanol (9:1 v/v) as eluent. The combined product fractions were evaporated yielding the desired product.

#### 3.2.4 Synthesis of 2‐(3‐Chloropropoxy)‐3a,4,7,7a‐Tetrahydro‐1H‐4,7‐Epoxyisoindole‐1,3 (2H) ‐Dione (H)

A solution of absolute ethanol (20 mL) and sodium hydroxide (1 equiv.) was reacted with commercially available endo‐N‐hydroxy‐5‐norbornene‐2,3‐dicarboximide (2.00 g, 11 mmol) and 1‐bromo‐3‐chloropropane (1 equiv.) at 70 °C for 24 h. Later, the mixture was cooled to room temperature. The residue was evaporated and after dissolution in DCM, was washed with water and brine. The combined organic layers were dried on anhydrous Na_2_SO_4_ and concentrated in vacuo. The crude product was purified by silica gel open chromatography using DCM/methanol (9:1 v/v) as eluent. The combined and evaporated product fractions were crystallized from diethyl ether yielding the desired product.

#### 3.2.5 Synthesis of N‐(3‐Chloropropyl) Bicyclo [2.2.1] hept‐5‐ene‐2‐Carboxamide (J)

Commercially available 5‐norbornene‐2‐carboxilic acid (mixture of *endo* and *exo*, predominantly *endo*) (5.00 g, 36 mmol) was solubilized in acetonitrile (50 mL) and cooled to 0 °C for 30 min. DCC (1.1 equiv.) and HOBt (1.1 equiv.) were added and the mixture was stirred for one hour. Finally, TEA (1.1 equiv.) and 3‐chloropropylamine hydrochloride (1 equiv.) were added, and the reaction mixture was stirred at room temperature for 8 h. When the reaction was completed, it was cooled to 0 °C. Subsequently, the mixture was filtered and evaporated under reduced pressure. The resulting residue was diluted with DCM and washed with NaHCO_3_ and brine. The organic phase was dried over Na_2_SO_4_ and concentrated, yielding the desired intermediate as a brown oil, the mixture of *exo*/*endo* isomers of which was used without further purification in the next steps.

### 3.3 General Procedures for the Synthesis of FG Derivatives (FG 1‐18)

To a solution of the corresponding starting nucleus (**B**, **J**, 0.500 g) in acetonitrile (30 mL), sodium iodide (1.1 equiv.) was added. The mixture was heated at reflux and stirred for 30 min. Then, the appropriate 4‐X‐substitued‐piperazine (1 equiv.) and anhydrous K_2_CO_3_ (1 equiv.) were added. The reaction was stirred at 80 °C for 24 h. Regarding the starting nucleus **D**, **F**, **H** (0.500 g), they were solubilized in acetonitrile (30 mL) together with sodium iodide (1.5 equiv.), and the mixture was stirred under reflux for 30 min. Then the appropriate 4‐X‐substituted piperazine (1 equiv.) and anhydrous K_2_CO_3_ (1.5 equiv.) were added. The reaction was stirred at 80 °C for 24h. In both cases, after cooling to room temperature, the mixture was filtered, concentrated to dryness and the residue was dissolved in dichloromethane (20 mL) and washed with water and brine. The organic layer was dried over anhydrous Na_2_SO_4,_ and the solvent removed under vacuum. The crude product was purified by silica gel open chromatography using DCM/methanol (9:1 v/v) as eluent. The combined and evaporated product fractions were crystallized from diethyl ether or converted into the corresponding hydrochloride salt, yielding the desired products (FG 1–18) as white solids

#### 3.3.1 Synthesis of N‐(3‐(4‐(2‐(4,5‐Dihydrothiazol‐2‐yl) Phenyl) Piperazin‐1‐Yl) Propyl) Picolinamide (FG‐1)

Following the synthetic procedure reported above, **FG‐1** was synthetized starting from **B** (0.500 g, 2.52 mmol) and **2a** (1 equiv.). The final compound was obtained as pure form after conversion in the corresponding oxalate salt.

#### 3.3.2 Synthesis of N‐(3‐(4‐(3‐(4,5‐Dihydrothiazol‐2‐yl) Phenyl) Piperazin‐1‐Yl) Propyl) Picolinamide (FG‐2)

Following the synthetic procedure reported above, **FG‐2** was synthetized starting from **B** (0.500 g, 2.52 mmol) and **2b** (1 equiv.). Final compound was obtained as pure form after conversion in the corresponding hydrochloride salts adding HCl ethereal solution to an ethanolic solution of the free base.

#### 3.3.3 Synthesis of N‐(3‐(4‐(4‐(4,5‐Dihydrothiazol‐2‐yl) Phenyl) Piperazin‐1‐Yl) Propyl) Picolinamide (FG‐3)

Following the synthetic procedure reported above, **FG‐3** was synthetized starting from **B** (0.500 g, 2.52 mmol) and **2c** (1 equiv.). Final compound was crystallized from diethyl ether.

#### 3.3.4 Synthesis of N‐(3‐(4‐(2‐(4,5‐Dihydrothiazol‐2‐yl) Phenyl) Piperazin‐1‐Yl) Propyl) Isonicotinamide (FG‐4)

Following the synthetic procedure reported above, **FG‐4** was synthetized starting from **D** (0.500 g, 2.52 mmol) and **2a** (1 equiv.). The final compound was obtained as pure form after conversion in the corresponding oxalate salt.

#### 3.3.5 Synthesis of N‐(3‐(4‐(3‐(4,5‐Dihydrothiazol‐2‐yl) Phenyl) Piperazin‐1‐Yl) Propyl) Isonicotinamide (FG‐5)

Following the synthetic procedure reported above, **FG‐5** was synthetized starting from **D** (0.500 g, 2.52 mmol) and **2b** (1 equiv.). Final compound was crystallized from diethyl ether.

#### 3.3.6 Synthesis of N‐(3‐(4‐(4‐(4,5‐Dihydrothiazol‐2‐yl) Phenyl) Piperazin‐1‐Yl) Propyl) Isonicotinamide (FG‐6)

Following the synthetic procedure reported above, **FG‐6** was synthetized starting from **D** (0.500 g, 2.52 mmol) and **2c** (1 equiv.). Final compound was crystallized from diethyl ether.

#### 3.3.7 Synthesis of 2‐(3‐(4‐(2‐(4,5‐Dihydrothiazol‐2‐yl) Phenyl) Piperazin‐1‐Yl) Propoxy)‐3a,4,7,7a‐Tetrahydro‐1H‐4,7‐Methanoisoindole‐1,3 (2H) ‐Dione (FG‐7)

Following the synthetic procedure reported above, **FG‐7** was synthetized starting from **F** (0.500 g, 2.00 mmol) and **2a** (1 equiv.). Final compound was crystallized from diethyl ether.

#### 3.3.8 Synthesis of 2‐(3‐(4‐(3‐(4,5‐Dihydrothiazol‐2‐yl) Phenyl) Piperazin‐1‐Yl) Propoxy)‐3a,4,7,7a‐Tetrahydro‐1 H‐4,7‐Methanoisoindole‐1,3(2 H)‐Dione (FG‐8)

Following the synthetic procedure reported above, **FG‐8** was synthetized starting from **F** (0.500 g, 2.00 mmol) and **2b** (1 equiv.). Final compound was obtained as pure form after conversion in the corresponding hydrochloride salts adding HCl ethereal solution to an ethanolic solution of the free base.

#### 3.3.9 Synthesis of 2‐(3‐(4‐(4‐(4,5‐Dihydrothiazol‐2‐yl) Phenyl) Piperazin‐1‐Yl) Propoxy)‐3a,4,7,7a‐Tetrahydro‐1H‐4,7‐Methanoisoindole‐1,3 (2H) ‐Dione (FG‐9)

Following the synthetic procedure reported above, **FG‐9** was synthetized starting from **F** (0.500 g, 2.00 mmol) and **2c** (1 equiv.). Final compound was crystallized from diethyl ether.

#### 3.3.10 Synthesis of 2‐(3‐(4‐(2‐(4,5‐dihydrothiazol‐2‐yl) phenyl) piperazin‐1‐yl) propoxy)‐3a,4,7,7a‐tetrahydro‐1H‐4,7‐epoxyisoindole‐1,3(2H)‐dione (FG‐10)

Following the synthetic procedure reported above, **FG‐10** was synthetized starting from **H** (0.500 g, 2.00 mmol) and **2a** (1 equiv.). Final compound was crystallized from diethyl ether.

#### 3.3.11 Synthesis of 2‐(3‐(4‐(3‐(4,5‐dihydrothiazol‐2‐yl) phenyl) piperazin‐1‐yl) propoxy)‐3a,4,7,7a‐tetrahydro‐1H‐4,7‐epoxyisoindole‐1,3(2H)‐dione (FG‐11)

Following the synthetic procedure reported above, **FG‐11** was synthetized starting from **H** (0.500 g, 2.00 mmol) and **2b** (1 equiv.). Final compound was crystallized from diethyl ether.

#### 3.3.12 Synthesis of 2‐(3‐(4‐(4‐(4,5‐dihydrothiazol‐2‐yl) phenyl) piperazin‐1‐yl) propoxy)‐3a,4,7,7a‐tetrahydro‐1H‐4,7‐epoxyisoindole‐1,3(2H)‐dione (FG‐12)

Following the synthetic procedure reported above, **FG‐12** was synthetized starting from **H** (0.500 g, 2.00 mmol) and **2c** (1 equiv.). Final compound was crystallized from diethyl ether.

#### 3.3.13 Synthesis of Exo‐N‐(3‐(4‐(2‐(4,5‐dihydrothiazol‐2‐yl) phenyl) piperazin‐1‐yl) propyl) bicyclo [2.2.1] hept‐5‐ene‐2‐carboxamide (FG‐13) and Endo‐N‐(3‐(4‐(2‐(4,5‐dihydrothiazol‐2‐yl) phenyl) piperazin‐1‐yl) propyl) bicyclo[2.2.1] hept‐5‐ene‐2‐carboxamide (FG‐16)

Following the synthetic procedure reported above, **FG‐13** and **FG‐16** were synthetized starting from **J** (0.500 g, 2.34 mmol) and **2a** (1 equiv.). After the purification by silica gel open chromatography using DCM/methanol obtaining the final compound as a mixture of *endo* and *eso* isomers, the separation of two isomers was carried out by silica gel open chromatography with diethyl ether/methanol (8:2 v/v) as eluent. Final compounds were obtained as pure form after conversion in the corresponding oxalate salts.

#### 3.3.14 Synthesis of Exo‐ N‐(3‐(4‐(3‐(4,5‐dihydrothiazol‐2‐yl) phenyl) piperazin‐1‐yl) propyl) bicyclo [2.2.1] hept‐5‐ene‐2‐carboxamide (FG‐14) and Endo‐ N‐(3‐(4‐(3‐(4,5‐dihydrothiazol‐2‐yl) phenyl) piperazin‐1‐yl) propyl) bicyclo[2.2.1]hept‐5‐ene‐2‐carboxamide (FG‐17)

Following the synthetic procedure reported above, **FG‐14** and **FG‐17** were synthetized starting from **J** (0.500 g, 2.34 mmol) and **2b** (1 equiv.). After the purification by silica gel open chromatography using DCM/methanol obtaining the final compound as a mixture of *endo* and *eso* isomers, the separation of two isomers was carried out by silica gel open chromatography with diethyl ether/methanol (8:2 v/v) as eluent. Final compounds were obtained as pure form after crystallization from diethyl ether (**FG‐14**) and conversion in the corresponding oxalate salt (**FG‐17**).

#### 3.3.15 Synthesis of Exo‐ N‐(3‐(4‐(4‐(4,5‐dihydrothiazol‐2‐yl) phenyl) piperazin‐1‐yl) propyl) bicyclo [2.2.1] hept‐5‐ene‐2‐carboxamide (FG‐15) and Endo‐ N‐(3‐(4‐(4‐(4,5‐dihydrothiazol‐2‐yl) phenyl) piperazin‐1‐yl) propyl) bicyclo[2.2.1]hept‐5‐ene‐2‐carboxamide (FG‐18)

Following the synthetic procedure reported above, **FG‐15** and **FG‐18** were synthetized starting from **J** (0.500 g, 2.34 mmol) and **2c** (1 equiv.). After the purification by silica gel open chromatography using DCM/methanol obtaining the final compound as a mixture of *endo* and *eso* isomers, the separation of two isomers was carried out by silica gel open chromatography with diethyl ether/methanol (8:2 v/v) as eluent. Final compounds were crystallized from diethyl ether.

### 3.4 Chemical Characterization

**N‐(3‐chloropropyl) picolinamide** **(B,** Yield: 69%); Mp: 134‐135 °C. ^1^H NMR (400 MHz, CDCl_3_) d: 1.86 (m, 2H,–CH_2_ CH_2_‐CH_2_, *J* = 5.4); 3.60 (q, 2H,–NH‐CH_2_, *J* = 5.4); 3.86 (t, 2H, CH_2_‐Cl, *J* = 5.4); 7.35 (t, 1H, *J* = 7.0); 7.83 (t, 1H, *J* = 7.0); 8.18 (d, 1H, *J* = 7.6); 8.42 (d, 1H, *J* = 5.8); 9.02 (bs, 1H, NH).

**N‐(3‐(4‐(2‐(4,5‐dihydrothiazol‐2‐yl) phenyl) piperazin‐1‐yl) propyl) picolinamide** (**FG‐1**, Yield: 77%), Mp: 60–61 °C. ^1^H NMR (400 MHz, CDCl_3_) δ: 1.85 (m, 2H,–CH_2_‐CH_2_‐CH_2_,); 2.62 (t, 2H, CH_2_‐Npip, *J* = 6.4 Hz); 2.74 (bs, 4H, 2CH_2_ pip.); 3.09 (bs, 4H, 2CH_2_ pip.); 3.24 (t, 2H, ‐CH_2,_ *J* = 8.3 Hz) 3.60 (q, 2H,–NH‐CH_2,_ *J* = 6.00 Hz); 4.29 (t, 2H, ‐CH_2,_ *J* = 8.3 Hz); 7.09 (t, 1H, *J* = 7.5 Hz); 7.15 (d, 1H, *J* = 8.0 Hz); 7.40 (m, 2H,); 7.77 (d, 1H, *J* = 7.7 Hz); 7.83 (t, 1H, *J* = 7.7 Hz); 8.20 (d, 1H, *J* = 7.8 Hz); 8.51 (d, 1H, *J* = 4.2 Hz); 8.99 (bs, 1H, NH). ^13^C NMR (101 MHz, CDCl_3_) δ: 25.93; 33.44; 39.42; 53.02; 53.31; 57.66; 63.07; 119.48; 122.39; 123.26; 126.13; 129.42; 130.28; 131.33; 137.37; 148.17; 150.47; 151.90; 164.58; 168.32. ESI‐MS m/z [M+H]^+^ calculated for C_22_H_27_N_5_OS 409.55 Found = 410.4. Anal. Calcd for C_22_H_27_N_5_OS: C, 64.52; H, 6.65; N, 17.10. Found C, 64.58; H, 6.66; N, 17.16.

**N‐(3‐(4‐(3‐(4,5‐dihydrothiazol‐2‐yl) phenyl) piperazin‐1‐yl) propyl) picolinamide (FG‐2**, Yield: 73%) Mp: 97‐98 °C. ^1^H NMR (400 MHz, CDCl_3_) δ: 1.85 (m, 2H,–CH_2_‐CH_2_‐CH_2_) ; 2.59 (t, 2H, CH_2_‐Npip., *J* = 6.4 Hz); 2.66 (m, 4H, 2CH_2_ pip.); 3.34 (m, 4H, 2CH_2_ pip.); 3.40 (t, 2H, ‐CH_2,_ *J* = 8.3 Hz); 3.60 (q, 2H,–NH‐CH_2_, *J* = 5.9 Hz); 4.45 (t, 2H, ‐CH_2_, *J* = 8.3 Hz); 7.03 (m,1H); 7.30 (m, 2H,); 7.35 (t, 1H, *J* = 7.5 Hz); 7.44 (s, 1H,); 7.81 (t, 1H, *J* = 8.4 Hz); 8.18 (d, 1H, *J* = 7.8 Hz); 8.42 (d, 1H, *J* = 4.5 Hz); 9.06 (bs, 1H, NH). ^13^C NMR (101 MHz, CDCl_3_) δ: 25.76; 33.69; 39.42; 48.91; 53.33; 57.61; 63.30; 115.21; 118.72; 119.98; 122.26; 126.11; 129.34; 134.17; 137.29; 148.16; 150.32; 151.50; 164.55; 169.09. ESI‐MS m/z [M+H]^+^ calculated for C_22_H_27_N_5_OS 409.55, Found = 410.3. Anal. Calcd for C_22_H_27_N_5_OS: C, 64.52; H, 6.65; N, 17.10. Found C, 64.71; H, 6.64; N, 17.04.

**N‐(3‐(4‐(4‐(4,5‐dihydrothiazol‐2‐yl) phenyl) piperazin‐1‐yl) propyl) picolinamide (FG‐3**, Yield: 54%), Mp: 134‐135 °C. ^1^H NMR (400 MHz, CDCl_3_) δ: 1.85 (m, 2H,–CH_2_‐CH_2_‐CH_2_) ; 2.60 (t, 2H, CH_2_‐Npip., *J* = 6.3 Hz); 2.65 (m, 4H, 2CH_2_ pip.); 3.39 (m, 6H); 3.61 (q, 2H,–NH‐CH_2_, *J* = 5.9 Hz); 4.42 (t, 2H, ‐CH_2_, *J* = 8.2 Hz); 6.90 (d, 2H, *J* = 8.9 Hz); 7.35 (t, 1H, *J* = 7.5 Hz); 7.74 (d, 2H, *J* = 8.8 Hz); 7.81 (t, 1H, *J* = 8.5 Hz); 8.18 (d, 1H, *J* = 7.8 Hz); 8.38 (d, 1H, *J* = 4.6 Hz); 9.12 (bs, 1H, NH). ^13^C NMR (101 MHz, CDCl_3_) δ: 25.73; 33.69; 39.56; 48.02; 53.22; 57.75; 65.14; 114.48; 122.33; 123.95; 126.16; 129.87; 137.36; 148.13; 150.38; 153.36; 164.56; 168.00. ESI‐MS m/z [M+H]^+^ calculated for C_22_H_27_N_5_OS 409.55, Found = 410.3. Anal. Calcd for C_22_H_27_N_5_OS: C, 64.52; H, 6.65; N, 17.10. Found C, 64.32; H, 6.63; N, 17.06.

**N‐(3‐chloropropyl) isonicotinamide (D**, Yield: 93%). Mp 116‐118 °C. ^1^H NMR (400 MHz, CDCl_3_) d: 1.86 (m, 2H, CH_2_‐CH_2_‐CH_2_, *J* = 5.4); 3.60 (q, 2H, ‐NH‐CH_2_); 3.86 (t, 2H, CH_2_‐Cl, *J* = 5.4); 7.61 (d, 2H, *J* = 5.8); 8.35 (bs,1H, NH); 8.69 (d, 2H, *J* = 5.8).

**N‐(3‐(4‐(2‐(4,5‐dihydrothiazol‐2‐yl) phenyl) piperazin‐1‐yl) propyl) isonicotinamide** (**FG‐4** Yield: 35%), Mp: 168‐169 °C. ^1^H NMR (400 MHz, CDCl_3_) δ: 1.84 (m, 2H,–CH_2_‐CH_2_‐CH_2_,); 2.68 (t, 2H, CH_2_‐Npip, *J* = 5.7 Hz); 2.76 (bs, 4H, 2CH_2_ pip.); 2.98 (bs, 4H, 2CH_2_ pip.); 3.23 (t, 2H, ‐CH_2,_ *J* = 8.3 Hz) 3.61 (q, 2H,–NH‐CH_2,_ *J* = 5.4 Hz); 4.28 (t, 2H, ‐CH_2,_ *J* = 8.3 Hz); 7.04 (t, 1H, *J* = 8.01 Hz); 7.12 (d, 1H, *J* = 7.5 Hz); 7.42 (t, 2H, *J* = 7.7 Hz); 7.69 (d, 1H, *J* = 5.7 Hz); 7.78 (d, 1H, *J* = 7.7 Hz); 8.66 (bs, 1H, NH); 8.73 (d, 2H, *J* = 5.7 Hz). ^13^C NMR (101 MHz, CDCl_3_) δ: 23.99; 33.44; 41.38; 53.16; 53.32; 58.69; 63.08; 119.45; 121.26; 123.88; 129.53; 130.37; 131.55; 142.18 150.61; 151.23; 165.47; 167.85. ESI‐MS m/z [M+H]^+^ calculated for C_22_H_27_N_5_OS 409.55, Found = 410.3. Anal. Calcd for C_22_H_27_N_5_OS: C, 64.52; H, 6.65; N, 17.10. Found C, 64.26; H, 6.67; N, 17.04.

**N‐(3‐(4‐(3‐(4,5‐dihydrothiazol‐2‐yl) phenyl) piperazin‐1‐yl) propyl) isonicotinamide (FG‐5**, Yield: 46%) Mp: 8788 °C. ^1^H NMR (400 MHz, CDCl_3_) δ: 1.85 (m, 2H,–CH_2_‐CH_2_‐CH_2_) ; 2.63 (t, 2H, CH_2_‐Npip., *J* = 6.4 Hz); 2.68 (m, 4H, 2CH_2_ pip.); 3.23 (m, 4H, 2CH_2_ pip.); 3.41 (t, 2H, ‐CH_2,_ *J* = 8.3 Hz); 3.60 (q, 2H,–NH‐CH_2_, *J* = 5.6 Hz); 4.45 (t, 2H, ‐CH_2_, *J* = 8.3 Hz); 6.99 (m, 1H,); 7.30 (t, 2H, *J* = 7.5 Hz); 7.43 (bs, 1H,); 7.63 (d, 2H, *J* = 8.4 Hz); 8.35 (bs, 1H, NH), 8.66 (d. 2H, *J* = 5.9 Hz). ^13^C NMR (101 MHz, CDCl_3_) δ: 24.24; 33.75; 41.08; 49.29; 53.45; 58.38; 65.29; 115.33; 118.95; 120.73; 121.09; 129.49; 134.28; 141.95; 150.63; 151.08; 165.46; 169.04. ESI‐MS m/z [M+H]^+^ calculated for C_22_H_27_N_5_OS 409.55, Found = 410.3. Anal. Calcd for C_22_H_27_N_5_OS: C, 64.52; H, 6.65; N, 17.10. Found C, 64.71: H, 6.62; N, 17.16.

**N‐(3‐(4‐(4‐(4,5‐dihydrothiazol‐2‐yl) phenyl) piperazin‐1‐yl) propyl) isonicotinamide (FG‐6,** Yield: 36%) Mp: 158–159 °C. ^1^H NMR (400 MHz, CDCl_3_) δ: 1.85 (m, 2H, –CH_2_‐CH_2_‐CH_2_) ; 2.64 (m, 6H) 3.29 (m, 4H, 2CH_2_ pip.); 3.38 (t, 2H, ‐CH_2_ *J* = 8.2 Hz); 3.61 (q, 2H,–NH‐CH_2_, *J* = 5.6 Hz); 4.41 (t, 2H, ‐CH_2_, *J* = 8.2 Hz); 6.86 (d, 2H, *J* = 8.8 Hz); 7.62 (d, 2H, *J* = 7.5 Hz); 7.74 (d, 2H, *J* = 8.8 Hz) 8.21 (bs, 1H, NH); 8.66 (d, 2H, *J* = 5.8 Hz). ^13^C NMR (101 MHz, CDCl_3_) δ: 24.39; 33.72; 41.01; 48.32; 53.30; 58.35; 65.15; 114.50; 121.04; 129.91; 133.72; 141.94; 150.67; 152.83; 165.44; 167.89. ESI‐MS m/z [M+H]^+^ calculated for C_22_H_27_N_5_OS 409.55, Found = 410.3. Anal. Calcd for C_22_H_27_N_5_OS: C, 64.52; H, 6.65; N, 17.10. Found C, 64.32; H, 6.63; N, 17.13.

**2‐(3‐chloropropoxy)‐3a,4,7,7a‐tetrahydro‐1H‐4,7‐methano isoindole‐1,3(2H)‐dione** (**F,** Yield: 65%). Mp: 59–61 °C; ^1^H NMR (400 MHz, CDCl_3_) d:1.50 (d, 1H *J* = 9.1); 1.76 (dt, 1H *J* = 9.1); 2.16 (q, 2H, *J* = 6.2); 3.18 (dd, 2H, *J* = 1.4, 2.8); 3.42 (s, 2H); 3.58 (t, 2H, *J* = 6.4); 4.09 (t, 2H, *J* = 5.9); 6.16 (t, 2H, *J* = 1.8).

**2‐(3‐(4‐(2‐(4,5‐dihydrothiazol‐2‐yl) phenyl) piperazin‐1‐yl) propoxy)‐3a,4,7,7a‐tetrahydro‐1 H‐4,7‐methanoisoindole‐1,3(2 H)‐dione (FG‐7,** Yield: 37%) Mp: 161‐162 °C. ^1^H NMR (400 MHz, CDCl_3_) δ: 1.51 (d, 1H, *J* = 8.8 Hz); 1.77 (dt, 1H, *J* = 8.9 Hz); 1.89 (m,2H); 2.59 (t, 2H, CH_2_‐Npip, *J* = 7.0 Hz); 2.69 (bs, 4H, 2CH_2_ pip.); 2.98 (bs, 4H, 2CH_2_ pip.); 3.19 (dd, 2H, *J* = 2.7, 1.4 Hz); 3.23 (t, 2H, CH_2_, *J* = 8.3 Hz); 3.43 (s, 2H); 4.04 (t, 2H, O‐CH_2_, *J* = 6.4 Hz); 4.28 (t, 2H, CH_2_, *J* = 8.3 Hz); 6.17 (s, 2H); 7.07 (t, 1H, *J* = 7.4 Hz); 7.12 (d, 1H, J= 8.1 Hz); 7.37 (t, 1H, *J* = 7.7 Hz); 7.76 (d, 1H, *J* = 8.8 Hz). ^13^C NMR (101 MHz, CDCl_3_) δ: 15.42; 25.82; 33.43; 42.77; 44.90; 48.03; 51.54; 53.06; 54.72; 63.00; 76.08; 119.78; 123.38; 129.45; 130.13; 134.71; 151.79; 168.29; 172.39. ESI‐MS m/z [M+H]^+^ calculated for C_25_H_30_N_4_O_3_S 466.20, Found = 467.3. Anal. Calcd for C_25_H_30_N_4_O_3_S: C, 64.35; H, 6.48; N, 12.01. Found C, 64.60; H, 6.46; N, 11.96.

**2‐(3‐(4‐(3‐(4,5‐dihydrothiazol‐2‐yl) phenyl) piperazin‐1‐yl) propoxy)‐3a,4,7,7a‐tetrahydro‐1H‐4,7‐methanoisoindole‐1,3(2H)‐dione** (**FG‐8**, Yield: 42%) Mp: 197–198 °C. ^1^H NMR (400 MHz, CDCl_3_) δ: 1.50 (d, 1H, *J* = 8.9 Hz); 1.76 (d, 1H, *J* = 9.0 Hz); 1.87 (m, 2H); 2.58 (m, 6H); 3.18 (dd, 2H, *J* = 2.7, 1.4 Hz); 3.23 (m, 4H, 2CH_2_ pip); 3.39 (s, 1H); 3.42 (t, 2H, ‐CH_2_, *J* = 8.2Hz); 3.48 (m, 1H); 4.04 (t, 2H, O‐CH_2_, *J* = 6.4 Hz); 4.44 (t, 2H, CH_2_, J= 8.3Hz); 6.16 (s, 2H); 7.00 (m, 1H); 7.28 (s, 2H); 7.41 (s, 1H).^13^C NMR (101 MHz, CDCl_3_) δ:15.41; 25.77; 33.72; 42.77; 44.89; 49.00; 51.53; 53.17; 54.55; 65.31; 75.93; 115.17; 118.76; 120.04; 129.31; 134.70; 151.41; 169.13; 172.39. ESI‐MS m/z [M+H]^+^ calculated for C_25_H_30_N_4_O_3_S 466.20, Found = 467.3. Anal. Calcd for C_25_H_30_N_4_O_3_S: C, 64.35; H, 6.48; N, 12.01. Found C, 64.22; H, 6.49; N, 12.04.

**2‐(3‐(4‐(4‐(4,5‐dihydrothiazol‐2‐yl) phenyl) piperazin‐1‐yl) propoxy)‐3a,4,7,7a‐tetrahydro‐1H‐4,7‐methanoisoindole‐1,3(2H)‐dione** (**FG‐9**, Yield: 44%) Mp: 197–198 °C. ^1^H NMR (400 MHz, CDCl_3_) δ: 1.50 (d, 1H, *J* = 8.9 Hz); 1.76 (d, 1H, *J* = 9.0 Hz); 1.87 (m, 2H); 2.57 (m, 6H); 3.18 (dd, 2H, *J* = 2.7, 1.4Hz); 3.28 (m, 4H, 2CH_2_ pip); 3.36 (t, 2H, ‐CH_2_, *J* = 8.2Hz); 3.43 (s, 2H); 4.04 (t, 2H, O‐CH_2_, *J* = 6.3Hz); 4.40 (t, 2H, CH_2_, *J* = 8.2Hz); 6.16 (s, 2H); 6.87 (d, 2H, *J* = 8.8 Hz); 7.71 (d, 2H, *J* = 8.8 Hz). ^13^C NMR (101 MHz, CDCl_3_) δ: 15.41; 25.79; 33.66; 42.77; 44.89; 48.04; 51.54; 53.02; 54.52; 65.12; 75.87; 114.42; 123.89; 129.81; 134.70; 153.18; 167.94; 172.37. ESI‐MS m/z [M+H]^+^ calculated for C_25_H_30_N_4_O_3_S 466,20, Found = 467.4. Anal. Calcd for C_25_H_30_N_4_O_3_S: C, 64.35; H, 6.48; N, 12.01. Found C, 64.41; H, 6.49; N, 12.04.

**2‐(3‐chloropropoxy)‐3a,4,7,7a‐tetrahydro‐1H‐4,7‐epoxyisoindole‐1,3(2H)‐dione** (**H,** Yield: 58%) Mp: 72–73 °C; ^1^H NMR (400 MHz, CDCl_3_) d: 1.61 (s, 1H); 2.13 (m, 2H); 2.77 (s, 2H); 3.76 (t, 1H, *J* = 6.4 Hz); 4.24 (t, 2H, *J* = 5.9 Hz); 5.29 (s, 2H); 6.52 (s, 2H).

**2‐(3‐ (4‐ (2‐ (4,5‐dihydrothiazol‐2‐yl) phenyl) piperazin‐1‐yl) propoxy)‐3a, 4, 7, 7a‐tetrahydro‐ 1H‐ 4,7‐epoxyisoindole‐1,3 (2H)‐dione** (**FG‐10,** Yield: 30%) Mp: 155‐156 °C. ^1^H NMR (400 MHz, CDCl_3_) δ: 1.93 (m, 2H,–CH_2_‐CH_2_‐CH_2_,); 2.62 (t, 2H, CH_2_‐Npip, *J* = 7.2 Hz); 2.70 (bs, 4H, 2CH_2_ pip.); 2.76 (s, 2H); 2.99 (bs, 4H, 2CH_2_ pip.); 3.23 (t, 2H, ‐CH_2,_ *J* = 8.3 Hz); 4.18 (t, 2H, O‐CH_2_, *J* = 6.5 Hz); 4.28 (t, 2H, ‐CH_2_, *J* = 8.3 Hz); 5.29 (s, 2H); 6.52 (s, 2H); 7.08 (t, 1H, *J* = 7.4 Hz); 7.13 (d, 1H, *J* = 8.0 Hz); 7.38 (t, 1H, *J* = 7.7 Hz); 7.76 (d, 1H, *J* = 8.8 Hz). ^13^C NMR (101 MHz, CDCl_3_) δ: 25.72; 33.45; 44.20; 53.08; 53.14; 54.70; 63.02; 76.24; 80.63; 119.83; 123.38; 129.48; 130.15; 131.32; 136.32; 151.82; 168.28; 171.21. ESI‐MS m/z [M+H]^+^ calculated for C_24_H_28_N_4_O_4_S 468.57, Found = 469.3. Anal. Calcd for C_24_H_28_N_4_O_4_S: C, 61.52; H, 6.02; N, 11.96. Found C, 61.58; H, 6.00; N, 12.00.

**2‐(3‐(4‐(3‐(4,5‐dihydrothiazol‐2‐yl) phenyl) piperazin‐1‐yl) propoxy)‐3a,4,7,7a‐tetrahydro‐1H‐4,7‐epoxyisoindole‐1,3(2H)‐dione (FG‐11** Yield: 27%) Mp: 117‐118 °C. ^1^H NMR (400 MHz, CDCl_3_) δ: 1.93 (m, 2H,–CH_2_‐CH_2_‐CH_2_,); 2.59 (m, 6H); 2.76 (s, 2H); 3.25 (m, 4H, 2CH_2_ pip.); 3.39 (t, 2H, ‐CH_2,_ *J* = 8.3 Hz); 4.18 (t, 2H, O‐CH_2_, *J* = 6.4 Hz); 4.44 (t, 2H, ‐CH_2,_ *J* = 8.3 Hz); 5.29 (s, 2H); 6.51 (s, 2H); 7.01 (m, 1H); 7.28 (s, 2H, *J* = 8.0 Hz); 7.41 (s, 1H). ^13^C NMR (101 MHz, CDCl_3_) δ: 25.58; 33.73; 44.19; 48.94; 53.14; 54.55; 65.32; 76.05; 80.62; 115.22; 118.78; 120.08; 129.32;134.16; 136.31; 151.38; 169.11;171.20. ESI‐MS m/z [M+H]^+^ calculated for C_24_H_28_N_4_O_4_S 468.57, Found = 469.2. Anal. Calcd for C_24_H_28_N_4_O_4_S: C, 61.52; H, 6.02; N, 11.96. Found C, 61.33; H, 6.00; N, 11.91.

**2‐(3‐(4‐(4‐(4,5‐dihydrothiazol‐2‐yl) phenyl) piperazin‐1‐yl) propoxy)‐3a,4,7,7a‐tetrahydro‐1H‐4,7‐epoxyisoindole‐1,3(2H)‐dione (FG‐12** Yield: 30%) Mp: 134–135 °C. ^1^H NMR (400 MHz, CDCl_3_) δ: 1.91 (m, 2H,–CH_2_‐CH_2_‐CH_2_,); 2.58 (m, 6H); 2.75 (s, 2H); 3.28 (m, 4H, 2CH_2_ pip.); 3.36 (t, 2H, ‐CH_2_, *J* = 8.2Hz); 4.17 (t, 2H, O‐CH_2_, *J* = 6.4Hz); 4.40 (t, 2H, ‐CH_2,_ *J* = 8.2Hz); 5.29 (s, 2H); 6.51 (s, 2H); 6.87 (d, 2H, *J* = 8.8 Hz); 7.71 (d, 2H, *J* = 8.8 Hz). ^13^C NMR (101 MHz, CDCl_3_) δ: 25.65; 33.66; 44.19; 48.03; 53.01; 54.51; 65.12; 76.04; 80.63; 114.44; 123.88; 129.82; 136.31; 153.19; 167.98;171.20. ESI‐MS m/z [M+H]^+^ calculated for C_24_H_28_N_4_O_4_S 468.57, Found= 469.3. Anal. Calcd for C_24_H_28_N_4_O_4_S: C, 61.52; H, 6.02; N, 11.96. Found C, 61.39; H, 6.03; N, 11.99.

**N‐(3‐Chloropropyl)bicyclo[2.2.1]hept‐5‐ene‐2‐carboxamide** (**J,** Yield: 37%) **exo**: ^1^H NMR (400 MHz, CDCl_3_) :1.29 (d, 1H, *J* = 1.9 Hz), 1.31–1.36 (m, 1H), 1.68 (d, 1H, *J* = 8.2 Hz), 1.88–1.91 (m, 1H), 1.97–2.03 (m, 3H), 2.90 (s, 2H), 3.39 (q, 2H,‐NH‐CH2‐, *J* = 6.4 Hz), 3.56 (t, 2H, *J* = 6.3 Hz), 6.09 (dd, 1H, *J* = 2.2 Hz), 6.13(dd,1H, *J* = 2.2 Hz). ESI‐MS m/z [M+H]^+^calculated for C_11_H_16_ClNO 213.70, Found = 214.4

(**J**, Yield: 67%) **endo**: ^1^H NMR (400 MHz, CDCl_3_) :1.29 (d, 1H, *J* = 3.3 Hz), 1.32–1.33 (m, 1H), 1.44 (dd, 1H, *J* = 8.2, 2.0 Hz), 1.91–1.99 (m, 3H), 2.84–2.88 (m, 1H), 2.92 (s, 1H), 3.13 (s, 1H), 3.33–3.37 (m, 2H,‐NH‐CH_2_‐), 3.54 (t, 2H, *J* = 6.4 Hz), 5.95 (dd, 1H, *J* = 5.5, 2.7Hz), 6.22 (dd, 1H, J= 5.5, 3.1Hz). ESI‐MS m/z [M+H]^+^ calculated for C_11_H_16_ClNO 213.70, Found= 214.5.

**Exo‐N‐(3‐(4‐(2‐(4,5‐dihydrothiazol‐2‐yl) phenyl) piperazin‐1‐yl) propyl) bicyclo [2.2.1] hept‐5‐ene‐2‐carboxamide (FG‐13** Yield: 25%) Mp: 187–188 °C. ^1^H NMR (400 MHz, CDCl_3_) δ: 1.31(m, 2H); 1.64 (m, 2H); 1.94 (m, 1H); 1.99 (m, 1H); 2.56 (t, 2H, N‐CH_2_, *J* = 6.3); 2.72 (bs, 4H, 2CH_2_ pip.); 2.91 (s, 1H); 2.94 (s, 1H); 3.00 (bs, 4H, 2CH_2_ pip.); 3.13 (s, 1H), 3.24 (t, 2H, ‐CH_2_, *J* = 8.3 Hz); 3.38 (q, 2H); 4.29 (t, 2H, ‐CH_2_, *J* = 8.3Hz); 6.09 (dd, 1H, *J* = 5.6, 3.0Hz); 6.14 (dd, 1H, *J* = 5.6, 2.9Hz); 7.09 (dd, 2H, *J* = 8.0, 3.5, 1.4Hz); 7.39 (td, 1H, *J* = 8.0, 1.6 Hz); 7.77 (dd, 1H, *J* = 8.0, 1.6Hz). ^13^C NMR (101 MHz, CDCl_3_) δ: 25.35; 30.50; 33.47; 39.89; 41.72; 45.17; 46.54; 47.40; 53.23; 57.96; 63.09; 119.57;123.58; 129.46; 130.30; 131.40; 136.10; 138.45;151.49; 168;18; 175.52. ESI‐MS m/z [M+H]^+^ calculated for C_24_H_32_N_4_OS 424.61, Found = 425.4. Anal. Calcd for C_24_H_32_N_4_OS: C, 67.89; H, 7.60; N, 13.20. Found C, 68.02; H, 7.58; N, 13.18.

**Exo‐N‐(3‐(4‐(3‐(4,5‐dihydrothiazol‐2‐yl) phenyl) piperazin‐1‐yl) propyl) bicyclo [2.2.1] hept‐5‐ene‐2‐carboxamide (FG‐14,** Yield: 36%) Mp: 162–163 °C. ^1^H NMR (400 MHz, CDCl_3_) δ: 1.33 (m, 2H); 1.70 (m, 2H); 1.90 (m, 2H,); 2.49 (t, 2H, N‐CH_2_, *J* = 6.4); 2.63 (m, 4H, 2CH_2_ pip.); 2.83 (m, 1H); 2.89 (s, 1H); 3.13 (s, 1H); 3.29 (m, 6H); 3.40 (t, 2H, ‐CH_2_, *J* = 8.3Hz); 4.45 (t, 2H, ‐CH_2_, *J* = 8.3Hz); 5.92 (dd, 1H, *J* = 5.3, 2.6Hz); 6.20 (dd, 1H, *J* = 5.3, 3.0Hz); 7.02 (m, 1H); 7.28 (s, 2H), 7.43 (s, 1H). ^13^C NMR (101 MHz, CDCl_3_) δ: 25.40; 30.54; 33.75; 39.71; 42.81; 45.07; 46.51; 49.03; 50.04; 53.42; 57.57; 65.32; 115.15; 118.81; 120.33; 129.40; 132.60; 134.24; 137.70; 151.19; 169.10; 174.33. ESI‐MS m/z [M+H]^+^ calculated for C_24_H_32_N_4_OS 424.61, Found = 425.4. Anal. Calcd for C_24_H_32_N_4_OS: C, 67.89; H, 7.60; N, 13.20. Found C, 67.75; H, 7.62; N, 13.25.

**Exo‐N‐(3‐(4‐(4‐(4,5‐dihydrothiazol‐2‐yl) phenyl) piperazin‐1‐yl) propyl) bicyclo [2.2.1] hept‐5‐ene‐2‐carboxamide** (**FG‐15** Yield: 15%) Mp: 184–185 °C. ^1^H NMR (400 MHz, CDCl_3_) δ: 1.31(m, 2H); 1.64 (m, 2H); 1.93 (m, 2H); 2.52 (t, 2H, N‐CH_2_, *J* = 6.3Hz); 2.62 (m, 5H, 2CH_2_ pip.); 2.91 (d, 2H, *J* = 9.0 Hz); 3.30 (m, 4H, 2CH_2_ pip.); 3.38 (m, 4H); 4.41 (t, 2H, ‐CH_2_, *J* = 8.2Hz); 6.05 (dd, 1H, *J* = 5.6, 3.0 Hz); 6.11 (dd, 1H, *J* = 5.3, 2.8Hz); 6.88 (d, 2H, *J* = 8.8 Hz); 7.73 (d, 2H, *J* = 8.7 Hz). ^13^C NMR (101 MHz, CDCl_3_) δ: 25.52; 30.54; 33.69; 39.66; 41.70; 45.11; 46.53; 47.38; 48.24; 53.20; 57.69; 65.14; 114.55; 124.24; 129.87; 136.07; 138.44; 152.98; 167.91; 175.54. ESI‐MS m/z [M+H]^+^ calculated for C_24_H_32_N_4_OS 424.61, Found = 425.4. Anal. Calcd for C_24_H_32_N_4_OS: C, 67.89; H, 7.60; N, 13.20. Found C, 67.95; H, 7.58; N, 13.17.

**Endo‐N‐(3‐(4‐(2‐(4,5‐dihydrothiazol‐2‐yl) phenyl) piperazin‐1‐yl) propyl) bicyclo[2.2.1] hept‐5‐ene‐2‐carboxamide (FG‐16,** Yield: 58%) Mp: 156–157 °C. ^1^H NMR (400 MHz, CDCl_3_) δ: 1,29 (d, 1H, *J* = 8.2 Hz); 1.36 (m, 1H); 1.44 (m, 1H); 1.69 (m, 1H); 1.93 (m, 1H); 2.52 (t, 2H, N‐CH_2_, *J* = 6.4); 2.71 (bs, 4H, 2CH_2_ pip.); 2.85 (m, 1H); 2.91 (s, 1H); 3.02 (bs, 4H, 2CH_2_ pip.); 3.16 (s, 1H); 3.24 (t, 2H, ‐CH_2_, *J* = 8.3 Hz); 3.31 (q, 2H); 4.29 (t, 2H, ‐CH_2_, *J* = 8.3 Hz); 5.98 (dd, 1H, *J* = 5.5, 2.7 Hz); 6.21 (dd, 1H, *J* = 5.5, 3.0 Hz); 7.10 (m, 2H); 7.39 (m, 1H); 7.77 (dd, 1H, *J* = 7.7, 1.3). ^13^C NMR (101 MHz, CDCl_3_) δ: 25.53; 29.98; 33.56; 39.44; 42.94; 45.08; 46.29; 50.03; 53.30; 57.65; 63.07; 119.58; 123.55; 129.45; 130.28; 131.39; 132.60; 137.52; 151.51; 168;14; 174.27. ESI‐MS m/z [M+H]^+^ calculated for C_24_H_32_N_4_OS 424.61, Found = 425.5. Anal. Calcd for C_24_H_32_N_4_OS: C, 67.89; H, 7.60; N, 13.20. Found C, 67.82; H, 7.61; N, 13.16.

**Endo‐N‐(3‐(4‐(3‐(4,5‐dihydrothiazol‐2‐yl) phenyl) piperazin‐1‐yl) propyl) bicyclo[2.2.1]hept‐5‐ene‐2‐carboxamide (FG‐17,** Yield: 18%) Mp: 171–172 °C. ^1^H NMR (400 MHz, CDCl_3_) δ: 1.23 (d, 1H, *J* = 8.3 Hz); 1.31 (m, 1H); 1.40 (m, 1H); 1.69 (m, 2H); 1.88 (m, 1H); 2.46 (t, 2H, N‐CH_2_, *J* = 6.4); 2.60 (m, 4H, 2CH_2_ pip.); 2.81 (m, 1H); 2.87 (s, 1H); 3.10 (s, 1H); 3.24 (m, 6H); 3.38 (t, 2H, ‐CH_2_, *J* = 8.3Hz); 4.43 (t, 2H, ‐CH_2_, *J* = 8.3Hz); 5.96 (dd, 1H, *J* = 5.6, 2.8Hz); 6.18 (dd, 1H, *J* = 5.6, 3.1Hz); 7.00 (m, 1H); 7.27 (s, 2H); 7.41 (s, 1H). ^13^C NMR (101 MHz, CDCl_3_) δ: 25.57; 30.00; 33.74; 39.37; 42.80; 45.07; 46.27; 49.06; 50.03; 53.43; 57.60; 65.32; 115.12; 118.79; 120.30; 129.39; 132.60; 134.23; 137.69; 151.21; 169.11; 174.31. ESI‐MS m/z [M+H]^+^ calculated for C_24_H_32_N_4_OS 424.61, Found = 425.3. Anal. Calcd for C_24_H_32_N_4_OS: C, 67.89; H, 7.60; N, 13.20. Found C, 68.09; H, 7.61; N, 13.25.

**Endo‐ N‐(3‐(4‐(4‐(4,5‐dihydrothiazol‐2‐yl) phenyl) piperazin‐1‐yl) propyl) bicyclo[2.2.1]hept‐5‐ene‐2‐carboxamide** (**FG‐18** Yield: 11%) Mp: 163–164 °C. ^1^H NMR (400 MHz, CDCl_3_) δ: 1.27 (d, 1H, 8.4Hz); 1.33 (m, 1H); 1.43 (m, 1H); 1.69 (m, 2H); 1.91 (m, 1H); 2.47 (t, 2H, N‐CH_2_, *J* = 6.4Hz); 2.61 (m, 4H, 2CH_2_ pip.); 2.84 (m, 1H); 2.89 (s, 1H); 3.13 (s, 1H); 3.31 (m, 6H); 3.37 (t, 2H, ‐CH_2_, *J* = 8.2Hz); 4.41 (t, 2H, ‐CH_2_, *J* = 8.2Hz); 5.98 (dd, 1H, *J* = 5.4, 2.7Hz); 6.21 (dd, 1H, *J* = 5.4, 3.0Hz);6.88 (d, 2H, *J* = 8.8Hz); 7.73 (d, 2H, *J* = 8.8 Hz). ^13^C NMR (101 MHz, CDCl_3_) δ: 25.71; 30.01; 33.69; 39.28; 41.70; 45.09; 46.27; 48.14; 50.04; 53.28; 57.51; 65.13; 114.51; 124.16; 129.96; 136.07; 137.73; 153.00; 167.93; 174.29. ESI‐MS m/z [M+H]^+^ calculated for C_24_H_32_N_4_OS 424.61, Found = 425.4. Anal. Calcd for C_24_H_32_N_4_OS: C, 67.89; H, 7.60; N, 13.20. Found C, 68.09; H, 7.61; N, 13.17.

### 3.5 Prediction of Activity Spectra for Substances

ChemDraw Professional 16.0 was used to create the structures of target compounds **FG 1**‐**18**. Subsequently, Chem3D 16.0 was used to transform into the appropriate SD format. The SD files were used separately with the Academic PASS Standard version to predict the biological spectrum. The biological activity is predicted by comparing the structure of a new compound with the structure of a known biologically active molecule. By using the structural formula as input data, it is possible to estimate activity and generate biological activity profiles for virtual molecules before their chemical synthesis and biological evaluation. The algorithm used for activity prediction is based on a Bayesian approach. This tool calculates the probability ratio of activity (P_a_) to inactivity (P_i_), arranging the results in descending order of P_a_ − P_i_. This ensures that the most probable activities appear at the top of the list. Only activities where P_a_ > P_i_ are taken under investigation.

### 3.6 Ex vivo Receptor Assays

#### 3.6.1 5‐HT_1A_, 5‐HT_2A_, and 5‐HT_2C_ Competition Binding Assay

Sprague‐Dawley rats were sacrificed by isoflurane overdose. Brains were rapidly removed and placed on ice. Hippocampi (for the 5‐HT_1A_ assay) and frontal cortices (for the 5‐HT_2A_ and 5‐HT_2C_ assays) were dissected on a Petri dish. The tissue from 10 rats was homogenized in 30 vol. homogenization buffer (50 mM Tris–HCl, pH = 4.7, 1 mM EDTA, 1 mM dithiothreitol) with a handheld Teflon‐glass homogenizer. The homogenate was centrifuged at 48,000 × g at 4 °C for 15 min. The pellet was suspended and homogenized in the homogenization buffer and incubated for 10 min. at 36 °C. The centrifugation and suspension steps were repeated twice. The final pellet was homogenized in 5 vol. 50 mM Tris–HCl, pH = 7.4 buffer and stored at −80 °C for no longer than 6 months. Seven concentrations equally spaced on a log scale (10^−10^ M–10^−4^ M) of each compound were incubated in duplicate with either: 1 nM [^3^ H]8‐OH‐DPAT (specific activity: 200 Ci mmol^−1^, Revvity, MA, USA) for 60 min at 36 °C in a 50 mM Tris–HCl (pH 7.4) buffer, supplemented with 0.1% ascorbate, 5 mM MgCl_2_, 10μM pargyline and 80 μg of hippocampal membrane suspension (the 5‐HT_1A_ assay). For the 5‐HT_2A_ assay, 160 μg of frontal cortex membrane suspension was incubated with 1 nM [^3^H] ketanserin (specific activity: 22.8 Ci mmol^−1^, Revvity, MA, USA) for 60 min. at 36 °C in a 50 mM Tris‐HCl (pH 7.4) buffer, supplemented with 0.1% ascorbate, 10 μM pargyline and 3 mM CaCl_2_. For the 5‐HT_2C_ assay, compounds were incubated in duplicate with 5 nM [^3^H]mesulergine (specific activity: 80.2 Ci mmol^−1^, Revvity, MA, USA) for 60 min. at 36 °C in a 50 mM Tris‐HCl (pH 7.4) buffer, supplemented with 5 mM MgCl_2_, 140 mM NaCl, 1 mM DTT, 1 mM CaCl_2_, 10 μM pargyline and 160μg of the frontal cortex membrane suspension. Non‐specific binding was determined with 10μM serotonin. The final DMSO concentration in the assay was 5%. After incubation, the reaction mixture was deposited onto Unifilter GF/C plates (Revvity, MA, USA) presoaked in 0.4% PEI (or 0.4% PEI and 0.5% Triton X‐100 for the 5‐HT_2C_ assay) for 1 h with the FilterMate‐96 Harvester (Revvity, MA, USA). Then, each well was washed with 2 mL of 50 mM Tris–HCl (pH 7.4) buffer to separate bound ligands from free and the plates were left to dry overnight. Then, 35μl of Microscint‐20 scintillation fluid (Revvity, MA, USA) was added to each filter well and left to equilibrate for 2h. Filter‐bound radioactivity was counted in a MicroBeta2 LumiJet scintillation counter (Revvity, MA, USA). Binding curves were fitted with one site non‐linear regression. Binding affinity (pKi and Ki) for each compound was calculated from the EC_50_ values with the Cheng‐Prusoff equation from two separate experiments.

#### 3.6.2 5‐HT_1A_ Receptor Activation in the [^35^S] GTP‐γ‐S Assay

Seven compound concentrations equally spaced on a log scale (10–4 m to 10–10 m) were incubated in duplicate with hippocampal membrane preparations (5μg per well) in assay buffer (50 mM Tris‐HCl, pH = 7.4, 1 mM EGTA, 3 mM MgCl_2_, 100 mM NaCl, and 30 μM GDP) and 0.08 nM [^35^S]GTP‐γ‐S (specific activity: 1250 Ci mmole^−1^, Revvity). Nonspecific binding was determined with 10μM of unlabeled GTP‐γ‐S. The final DMSO concentration in the assay was 5%. The reaction mixture was incubated for 90 min. at 30 °C on an orbital shaker set at 250 rpm. Next, the reaction mixture was deposited under vacuum with the FilterMate Harvester (Revvity, USA) onto Unifilter GF/C Plates (Revvity, MA, USA) presoaked with wash buffer (50 mM Tris‐HCl, pH = 7.4). The wells were then rapidly washed with 2 mL of wash buffer. Filter plates were dried overnight at room temperature. Once completely dry, 35 μl of MicroScint PS (Revvity, MA, USA) scintillation fluid was added to each well. Radioactivity was counted in a MicroBeta2 LumiJet scintillation counter (Revvity, MA, USA). Data were analyzed with GraphPad Prism 5.0 software (GraphPad Software, San Diego California USA, www.graphpad.com). The curves were fitted with three‐parameter non‐linear regression model. Potency (EC_50_) and efficacy (E_max_) were calculated and expressed as means from two separate experiments ±95% confidence intervals (95% CI).

### 3.7 Ex Vivo Assays

#### 3.7.1 General Procedures

Male rats (Sprague‐Dawley, 160–200 g; Charles River Laboratories; Calco, Italy) were manipulated and cared for in strict compliance with the principles of laboratory animal care. Experimental procedures were conducted in conformity with Italian (D.L. 26/2014) and European (directive 2010/63/EU) regulations on the protection of animals used for scientific purposes and approved by the Italian Ministry. Animal housing complied with recent pharmacological guidance.^[^ ^40^ ^]^ All animals weighing 160–200 g were used after a 1‐week acclimation period (temperature 23 ± 2 °C; humidity 60%, free access to water and standard food).

#### 3.7.2 Ileum Preparation and Evaluation of 5‐HT‐Evoked Contractions

Rats were asphyxiated using CO_2_ and segments (1–1.5 cm) of ileum were removed, flushed of luminal contents, and placed in Krebs solution (119 mM NaCl, 4.75 mM KCl, 1.2 Mm KH_2_PO_4_, 25 mM NaHCO_3_, 2.5 mM CaCl_2_, 1.5 mM MgSO_4_, and 11 mM Glucose). The segments were prepared as previously described:^[^ ^41^ ^]^ the segments were set up in such a way as to record contractions mainly from the longitudinal axis, in an organ bath containing 20 mL of Krebs solution, bubbled with 95% O_2_ and 5% CO_2_ and maintained at 37 °C. The tissues were connected to an isotonic transducer (load: 0.5 g), connected to Power Lab system (Ugo Basile, Comerio, Italy). Ileal segments were equilibrated for 60 min^[^ ^16^ ^]^ followed by three repeated additions of submaximal concentration of 5‐HT (10^−5^ M) in order to record stable control contractions. To evaluate the inhibitory activity, the responses were observed in the presence of increasing concentrations (10^−8^–10^−5^ M). In preliminary experiments, the effect of 5‐HT was observed in the presence of the neuronal blocker tetrodotoxin (0.3 μM), the muscarinic receptor antagonist atropine (1 μM), the adrenergic receptor antagonists phentolamine (10^−6^ M) plus propranolol (10^−6^ M) and the 5‐HT_2A_ antagonist ketanserin (0.1 μM). The contact time for each concentration was 10 min. The compounds were dissolved in DMSO. DMSO (<0.01%) did not modify 5‐HT‐induced contractions. Results are expressed as mean (SEM). The concentration of the compounds that produced 50% inhibition of 5‐HT‐induced contractions (IC_50_) or maximal inhibitory effect (E_max_) were used to characterize compounds potency and efficacy, respectively. The IC_50_ and E_max_ values were calculated with the aid of a computer program (GraphPad Prism 5).

### 3.8 In Vivo Behavioral Tests

#### 3.8.1 General Procedures

The tests were performed on male white mice of the Albino Swiss strain, whose weight was between 24 and 30 g in Experimental Medicine Center, Medical University of Lublin, Poland. The animals were housed (four/five per cage). Before conducting the experimental tests, the animals were subjected to an adaptation that lasted seven days. This consisted of keeping them in a room with relatively constant conditions: a temperature of 22 ± 1 °C, a humidity of 55 ± 5% and lighting that mimicked the natural day and night rhythm (12 h/12 h). Constant access to food and water was provided. Standard feed—LSM Motycz pelleted feed—and tap water were used. Mice were randomly allocated to study groups. Each group was represented by 8–10 animals, depending on the research schedule. The experiments were performed according to the procedures set out in the regulations for animal experiments: National Institute of Health Guidelines for the Care and Use of Laboratory Animals (8th edition) and to the European Community Council Directive for the Care and Use of Laboratory Animals of 22 September 2010 (2010/63/EU). The study was approved by the Local Ethical Committee in Lublin (number of ethical approvals: 27/2023). Tested compounds: **FG‐1**, **FG‐4**, **FG‐5**, **FG‐6**, **FG‐7**, **FG‐8**, and **FG‐18** were finely ground in mortars with the addition of the emulsifier Tween‐80. A suspension was obtained, which was then diluted with aqueous solution of 0.5% methylcellulose (tylose). The mixture was then administered intraperitoneally (i.p.) to the animals 60 min before the start of the experiments. Animals in the control groups received a 0.5% tylose solution. All substances were administered via the same route of administration in appropriate dose and volume of 0.1 mL per 10 g of animal body weight. Prior to the administration of the drug, the animals were weighed, during the interval between tests, they were provided with food, water, and constant living conditions.

#### 3.8.2 Rota‐Rod and Chimney Tests

The motor coordination of mice was evaluated using the rota‐rod test (Ataner Zakład Uslugowy Elektroniki Inż K. Fic, Lublin, Poland) and in the chimney test. The rota‐rod technique was pioneered by Dunham and Miya^[^ ^42^ ^]^ and has proved very useful in preclinical studies for testing drugs affecting motor coordination. The rota‐rod consists of five 75 mm wide compartments, each with a long 3 cm diameter cylindrical rod rotating with a constant speed of 8 rev min^−1^. A pre‐test was performed before the actual experiment; for this purpose, animals were placed on the rod for 3 consecutive days for 3 min. Then, on the day of the test, only pre‐selected animals were studied, and they were kept on the rod up to 60s. These animals were administered the tested substances, and 60 min later were placed individually on a rod and the time they stayed on it was measured. The rota‐rod test is used to assess not only the rodent's motor coordination but also its sense of balance.^[^ ^11^ ^]^ The chimney test was introduced by Boisser^[^ ^43^ ^]^ as a simple test for the effects of sedatives and muscle relaxants. In this test, the animals’ impaired motor coordination was manifested as the inability of mice to climb backwards up a transparent plastic tube with a rough surface (3 cm internal diameter and 25 cm long) within 60s. Before the test, animals were trained once a day for three days. Animals that were capable of leaving the chimney for 60s were allowed to participate in the experiments. On the day of the test, the animal's escape from the tube before 60s indicates correct motor coordination.^[^ ^11^ ^]^

#### 3.8.3 Amphetamine‐Induced or MK‐801‐Induced Hyperactivity Test

An Opto‐Varimex‐4 Auto‐Track animal activity meter (Columbus Instruments, USA) was used to study animal movement, which consists of eight transparent cages with lids measuring 43 × 43 × 32 cm, a set of four infrared transmitters (each transmitter has 16 laser beams) and four detectors to monitor animal movements. Testing was initiated 60 min after compound administration. For the spontaneous locomotor activity study, tylose was administered to the control group and the tested compounds. For the amphetamine‐induced or MK‐801‐induced hyperactivity tests, tylose, the tested compounds and after 30 min, amphetamine (3 mg kg^−1^) or MK‐801 (0.3 mg kg^−1^) were administered. While the test was being performed, animals were individually caged, initially for 5 min of adaptation, followed by 20 min of actual mobility testing.

#### 3.8.4 Elevated plus Maze Test

The EPM test is performed using an apparatus developed by Lister;^[^ ^44^ ^]^ it consists of two open arms (30 × 5 cm) and two enclosed arms (30 × 5 × 15 cm) and a central platform (5 × 5 cm) it is placed at a height of 45 cm from the floor. The labyrinth was made of Plexiglas. The experiments were conducted in a darkened and quiet room. The central square of the maze was evenly illuminated with a dull red light. In the test, the mouse is observed for 5 min, and the number of entries into the open and closed arms and the time spent in them are recorded. The person carrying out the experiment remains constantly in the same place. Entering an individual arm is considered when the mouse has placed all four limbs outside the central square. The experiment measures: 1) the time spent by the mouse into the open arms of the maze, expressed as a percentage of the total exploration time, 2) the number of entries into the open arms of the maze, expressed as a percentage of the total number of entrances to both types of arms, and 3) the total number of entries into all arms of the maze—as a locomotor activity of the animals. If the mouse spends more time in the open arms than in the closed arms, this indicates an anxiolytic effect of the compounds tested. Exposed arms pose a risk of falling and therefore act as a mild stressor. Anxious mice are more likely to switch between open and enclosed arms and spend more time in enclosed arms than less anxious mice.^[^ ^11^, ^17^, ^44^, ^45^ ^]^

#### 3.8.5 Forced Swim Test (FST, Porsolt's Test)

The study was carried out using the test proposed by R. Porsolt.^[^ ^46^ ^]^ The method is based on the observation of an animal forced to swim in a confined space from which there is no escape. After an initial period of vigorous attempts to get out, the animal finally gives up on escaping. The test consists of immersing the mouse individually in a cylinder (diameter 10 cm, height 25 cm) filled with water (at a temperature of 23–25 °C) to a height of 10 cm for 6 min. In this test, two parameters were measured: immobility time in seconds (s), understood as the state in which the mouse makes only those movements that are necessary to keep the head above water, and the latency to the first immobility (s). Measurements were taken during the last 4 min of the experiment, starting from the 2nd to the 6th minute. At the end of each session, the animals were removed from the cylinders, dried with a towel, and placed near a heater until completely dry. Antidepressants typically show increased latency and decreased immobility in this test.

#### 3.8.6 DOI‐Induced Head‐Twitch Reaction

Head‐twitch reaction (HTR) is a very characteristic behavior of the head. The experiment employs a hallucinogenic substance, the psychedelic drug DOI, which in rodents, cause characteristic side‐to‐side movements of the head, commonly known as the HTR.^[^ ^47^ ^]^ DOI‐induced HTR was observed in a glass container (12 cm in diameter). Each mouse was individually transferred and acclimatized to the test environment for 30 min. Then, after administration of the tested compounds, DOI was immediately observed for 20 min, and the number of HTR was counted. Tested compounds were injected i.p. 60 min before the administration of DOI. The number of HTR was scored using a tally counter by an observer who did not know what group (control or tested group) was being studied.

#### 3.8.7 Body Temperature in Normothermic Mice

Body temperature in normothermic mice was measured in the rectum of animals by thermistor thermometer till 120 min after tested compounds injection. The mean value from the first two measurements (60 and 30 min before drug administration) was assumed as initial temperature (ti). The final temperature (tf) was measured 30, 60, 90, and 120 min after the injection of tested compounds. Body temperature changes (Δt) were calculated according to the formula: Δt = tf − ti.

#### 3.8.8 NOR Test

The setup for the NOR test consisted of a square, open‐ended Plexiglas box (46 cm long × 35 cm high × 33 cm wide), with a 10‐lux light source suspended 50 cm above it. Three objects were used for discrimination: a wooden prism, a blue plastic block, and a small ball‐shaped glass bottle. These objects were too heavy for the animals to move. The NOR test was performed according to established protocols.^[^ ^48^, ^49^ ^]^ On the day before the test, each mouse spent 15 min in an empty box to acclimate to the environment. On the experimental days, mice underwent three trials with breaks of 1 h and 24 h between them. Each trial lasted 5 min. In the first trial (T1), the box contained two identical objects (blue plastic blocks) placed in opposite corners, 10 cm from the side walls. The mouse was always placed in the center of the box. After T1, the mouse was returned to the cage. Then, after 1 h or 24 h, T2 and T3 were performed. At T2 or T3, one of the objects from T1 was replaced by a novel object (N), so that mice encountered one familiar (F) object and one novel (N) object. To avoid olfactory cues, both the box and the objects were cleaned after each mouse. Exploration was defined as pointing the nose at an object within 2 cm or touching the object with the nose. Behaviors such as turning away or sitting on the object were not considered exploration. The duration of exploration of each object at T1, T2, and T3 was manually recorded using a stopwatch. The discrimination between F and N during T2 or T3 was assessed by comparing the time spent exploring each of them. Memory performance was assessed using the DI, calculated as (N − F)/ (N + F), representing the difference in exploration time between N and F, normalized by the total exploration time for both objects. A higher DI indicates stronger retention of memory for familiar objects. The NOR test assessed both short‐term and long‐term memory based on the interval between the pretest and the subsequent retention test. Short‐term memory was assessed 1 h after the training trial, whereas long‐term memory was assessed after 24 h. Drugs were administered before the pretest, with the expectation that they might disrupt the physiological process of information acquisition.^[^ ^50^, ^51^ ^]^

### 3.9 Statistical Analysis

The results were calculated by two‐way analysis of variance (ANOVA) followed by Bonferroni post hoc test (body temperature) and one‐way analysis of variance ANOVA, followed by Dunnett's post hoc test (other tests). The results are presented as mean  ± standard errors (SEM). The level of *p* < 0.05 was considered as statistically significant. All the figures were prepared by the GraphPad Prism version 5.00 for Windows, GraphPad Software (San Diego, CA; www.graphpad.com).

### 3.10 Zebrafish Experiments

All experiments were conducted on zebrafish (*Danio rerio*) larvae up to 5 days post fertilization (dpf), therefore, in accordance with current European legislation, ethical permission to conduct assays was not required. However, housing and experimental procedures were performed in compliance with the National Institute of Health Guidelines for the Care and Use of Laboratory Animals and the European Union Directive of 22 September 2010 (2010/63/EU) on protecting animals used for scientific purposes. All efforts were taken to minimize larvae stress and suffering. Larvae were euthanized with an overdose of tricaine (15 μM). The zebrafish facility of Experimental Medicine Centre (Medical University of Lublin, Poland) provided wild‐type zebrafish embryos of the AB strain. Embryos as well as larvae were maintained in Petri dishes filled with E3 embryo medium, i.e., fish water (1.5 mM HEPES, pH 7.6; 17.4 mM NaCl; 0.21 mM KCl; 0.12 mM MgSO_4_; and 0.18 mM Ca (NO_3_)_2_). These were maintained under standard, generally accepted housing conditions (temperature 28.5 ± 1 °C, light to dark cycle 14 h to 10 h). Plastic transfer Pasteur pipettes were used to replace medium each day. Before the respective behavioral tests were conducted, larvae at 4 dpf were incubated for 20 h in different arylpiperazine derivatives solutions of maximum‐tolerated concentrations (MCTs), determined during toxicological screening.^[^ ^30^, ^32^ ^]^ The control group was exposed to equivalent concentration of DMSO solvent. All locomotor tracking tests were performed at the same period of the day. Basic locomotor activity was measured using an automated tracking device, Daniovision by Noldus, and the total distance traveled was quantified using EthoVision XT software of the same company (Wageningen, the Netherlands) over a 30 min long observation period. Larvae were placed in 48‐well plates, individually (single larva per well), using a plastic transfer pipette. The PTZ‐induced hyperlocomotion (acute seizure assay) was assessed in 5 dpf larval zebrafish (after 20 h incubation with tested derivatives at MCTs). After the incubation, larvae were exposed to an acute dose of PTZ (20 mM). Larval locomotor activity after exposure to PTZ was tracked (Daniovision by Noldus) over 30 mins, after 5‐mins of habituation. Additionally, larval locomotor activity was assessed in response to alternate lighting conditions (lights ON vs. lights OFF). Light intensity under illumination was 100%. Challenge to light/dark conditions lasted 10 min each, twice repeated.^[^ ^52^ ^]^ Similarly, the data were recorded as the distance traveled (mm) by larvae when exposed to alternating lightning. All experiments were repeated three times, and data pooled. For statistical analysis and figure generation, GraphPad Prism 9.3.1 version (San Diego, CA, USA) was employed, and Grubbs test was applied to identify outliers. Shapiro‐Wilk test was then employed to assess whether data are normally distributed. Moreover, nonparametric ANOVA, Kruskal–Wallis, or Friedman (for repeated measures) test was used to analyze data, with statistical significance level set at *p *< 0.05. Data are presented as median or median + individual values.

### 3.11 Molecular Docking

Induced fit docking^[^ ^53^ ^]^ from Schrödinger release 2019‐4 was utilized for molecular docking of the studied compounds to receptor models. Grid files were generated based on co‐crystallized ligands. Residues at the distance of 5 Å from the ligand pose were made flexible. Twenty poses were generated for each ligand–receptor complex. Visualization of molecular modeling results was achieved with Maestro Release 2019.4^[^ ^54^ ^]^ and PyMol 2.0.4^[^ ^55^ ^]^ software.

### 3.12 Protein Preparation

X‐ray structures of respective receptors were taken for molecular docking after necessary mutations: serotonin 5‐HT_2A_ receptor in complex with the antagonist risperidone (PDB ID: 6A93^[^ ^56^ ^]^), serotonin 5‐HT_2C_ receptor in complex with the agonist ergotamine (PDB ID: 6BQG^[^ ^57^ ^]^), and serotonin 5‐HT_1A_ receptor in complex with the agonist serotonin (PDB ID:7E2Y^[^ ^58^ ^]^). The structures of the biomolecules were preprocessed using the Protein Preparation Wizard of Maestro Release 2019.4^[^ ^54^ ^]^ to optimize the hydrogen bonding network and to remove any possible artefacts. The Yasara Structure v. 20.12.24^[^ ^59^ ^]^ tool for loop modeling was used to build receptors extracellular loops if necessary.

### 3.13 Ligand Preparation

The studied compounds **FG‐1**, **FG‐4**, **FG‐5**, **FG‐6**, **FG‐7**, **FG‐8**, and **FG‐18** were modeled using the LigPrep module and Epik modules of Schrödinger suite of software, v.2019‐4 as previously reported.^[^ ^60^ ^]^

## 4 Conclusion

We have described the synthesis of a new series of arylpiperazine derivatives as serotoninergic ligands (**FG 1‐18**). The 2‐(4,5‐dihydrothiazol‐2‐yl) phenyl piperazine derivatives (**FG‐7**) supporting an endo‐*N*‐hydroxy‐5‐norbornene‐2,3‐dicarboximide as terminal part fragment of LCAPs afforded an interesting affinity/selectivity binding profile associated to a favorable agonistic profile toward 5‐HT_1A_ with pEC_50_ values of 5.5 and a very low EC_50_ of 3.01 μM. Furthermore, **FG‐7, FG‐6**, and **FG‐8** showed higher or equal efficacy than the reference compound 8‐OH‐DPAT. In addition to the remarkable 5‐HT_1A_ receptor affinity and selectivity of compound **FG‐16** (Ki = 25 nM), other noteworthy Ki values were observed for compounds with the most favorable 5‐HT_2C_ affinity/selectivity profiles, such as **FG‐18** (Ki = 17 nM), **FG‐8** (Ki = 46 nM), and **FG‐14** (Ki = 72 nM). Finally, **FG‐14** ligand showed the best binding affinity toward the 5‐HT_2A_R, although not remarkable, but among the compounds tested in vitro to assess their activity on 5‐HT‐evoked contractions, only **FG‐8** exhibited a good IC_50_. Based on the in vitro results, the compounds **FG‐1**, **FG‐4**, **FG‐5**, **FG‐6**, **FG‐7**, **FG‐8, FG‐16**, and **FG‐18** were selected for further in vivo studies to investigate their functional activity. The results demonstrated that compounds **FG‐1**, **FG‐5**, **FG‐8**, and **FG‐6** exhibited clear antidepressant‐like effects. Notably, compounds **FG‐1**, **FG‐6**, **FG‐7**, and **FG‐18** also showed significant anxiolytic activity, and, in the EPM test, their efficacy was comparable to that of the reference drug buspirone. Compound **FG‐7**, in the NOR test, demonstrated notable pro‐cognitive properties by supporting memory consolidation. The measurement of the body temperature in normothermic mice further supports the correlation between the serotonergic system and mechanism of action of the studied compounds. In particular, the results of body temperature measurement these results (**Figure** **10**) allow for conclusion that the activity of compounds **FG‐5** and **FG‐6** could be an effect of a stimulation of 5‐HT_1A_ receptors. Finally, in addition to the in vivo test conducted in mouse models, further study performed in zebrafish models confirmed for **FG‐1** and **FG‐18** an already assessed anxiolytic activity. It is interesting to underline as the prediction analysis by PASS software conducted in the initial stages of design and synthesis revealed a general profile that categorized the compounds as anxiolytics, antipsychotics, antidepressants, or sedatives. These behaviors, correlated to interaction with serotonergic receptors, were first predicted and later concretely confirmed by receptor binding and functional activity assays. Anyway, **FG‐1** showed antidepressant, anxiolytic and anticonvulsant properties, **FG‐7** showed features as anxiolytic associated to pro‐cognitive properties and **FG‐18,** characterized by interesting 5‐HT_2C_ receptor affinity and selectivity associated to an anxiolytic profile, represents the most promising derivatives. Starting from this valuable basis, additional pharmacological studies are essential to fully elucidate the detailed mechanisms of action and assess the prospective clinical applicability of these compounds. In conclusion, the data presented in this study strong support the viability of the new scaffold 4,5‐dihydrothiazol‐2‐yl phenyl piperazine linked through a propylene chain with different nuclei. Several of the derivatives here reported displayed a clear preference for specific serotoninergic receptors, a tendency also corroborated by molecular docking studies. Furthermore, the compounds selected for their functional characterization, exhibit promising potential as antidepressant, anxiolytic or sedative agents. Consequently, future studies will focus on refining these findings and identifying key compounds to further investigate this evidence.

## Conflict of Interest

The authors declare no conflict of interest.

## Supporting information

Supplementary Material

## Data Availability

The data that support the findings of this study are available on request from the corresponding author. The data are not publicly available due to privacy or ethical restrictions.
