# Supplementary material for: Novel 4,5‐Dihydrothiazole‐Phenylpiperazine Derivatives: Synthesis, Docking Studies and Pharmacological Evaluation as Serotonergic Agents
Source: ChemMedChem. 2025 Jul 4;20(15):e202500288. doi: 10.1002/cmdc.202500288 (PMC12321279; doi:10.1002/cmdc.202500288)
Supplement: Supplementary file 1 — Supplementary Material [file CMDC-20-e202500288-s001.pdf]

Experimental Supporting Information

Novel 4,5-Dihydrothiazole-phenylpiperazine Derivatives: Synthesis, Docking Studies and Pharmacological Evaluation as Serotonergic Agents

Giorgia Andreozzi, [a] Natalia Karkoszka,[c,d] Rosa Sparaco, [a] Angela Corvino, [a] Beatrice Severino, [a] Vincenzo Santagada, [a] Elisa Magli, [b] Ewa Gibuła-Tarłowska, [c] Jolanta H. Kotlińska, [c] Kinga Gawel,[d] Raffaele Capasso, [e] Anna Lesniak, [f] Nataliia Semenko, [g] Agnieszka A. Kaczor, [h,i] Anna Bielenica,[j] Grażyna Biała, [c] Giuseppe Caliendo, [a] Ewa Kędzierska, #[c] and Ferdinando Fiorino #*[a]

[a] G. Andreozzi, R. Sparaco, A. Corvino, B. Severino, V. Santagada, G. Caliendo, F. Fiorino Dipartimento di Farmacia, Università degli Studi di Napoli Federico II, Via D. Montesano, 80131 Napoli, Italy.
[b] E. Magli Dipartimento di Sanità Pubblica, Università di Napoli Federico II, Via Pansini, 5, 80131, Naples, Italy
[c] N. Karkoszka, E. Gibuła-Tarłowska, J. H. Kotlińska, G. Biała, E. Kędzierska Department of Pharmacology and Pharmacodynamics, Medical University of Lublin, Chodźki 4a Str, 20-093 Lublin, Poland
[d] N. Karkoszka, K. Gawel Department of Experimental and Clinical Pharmacology, Jaczewskiego Str. 8b, 20-090 Lublin, Poland
[e] R. Capasso Dipartimento di Agraria, Università degli Studi di Napoli "Federico II", Via Università 100, 80055, Portici (Na), Italy
[f] A. Lesniak Faculty of Pharmacy, Department of Pharmacodynamics, Medical University of Warsaw, Centre for Preclinical Research and Technology, Warsaw, Poland
[g] N. Semenko Department of the Surgery, Anesthesiology and Intensive care, Bogomolets National Medical University, Kyiv, Ukraine
[h] A. A. Kaczor Department of Synthesis and Chemical Technology of Pharmaceutical Substances with Computer Modeling Laboratory, Faculty of Pharmacy, Medical University of Lublin, 4A Chodzki St., 20-093 Lublin, Poland
[i] A. A. Kaczor School of Pharmacy, University of Eastern Finland, Yliopistonranta 1, P.O. Box 1627, 70211 Kuopio, Finland
[j] A. Bielenica Chair and Department of Biochemistry, Medical University of Warsaw, Banacha 1 Str, Warsaw 02-097, Poland
# equally contributing authors
fefiorin@unina.it

Contents

1. ^{1}H, ^{13}C NMR and MS Spectra of Compounds **FG 1-18**

1. ^{1}H, ^{13}C NMR and MS Spectra of Compounds FG 1-18

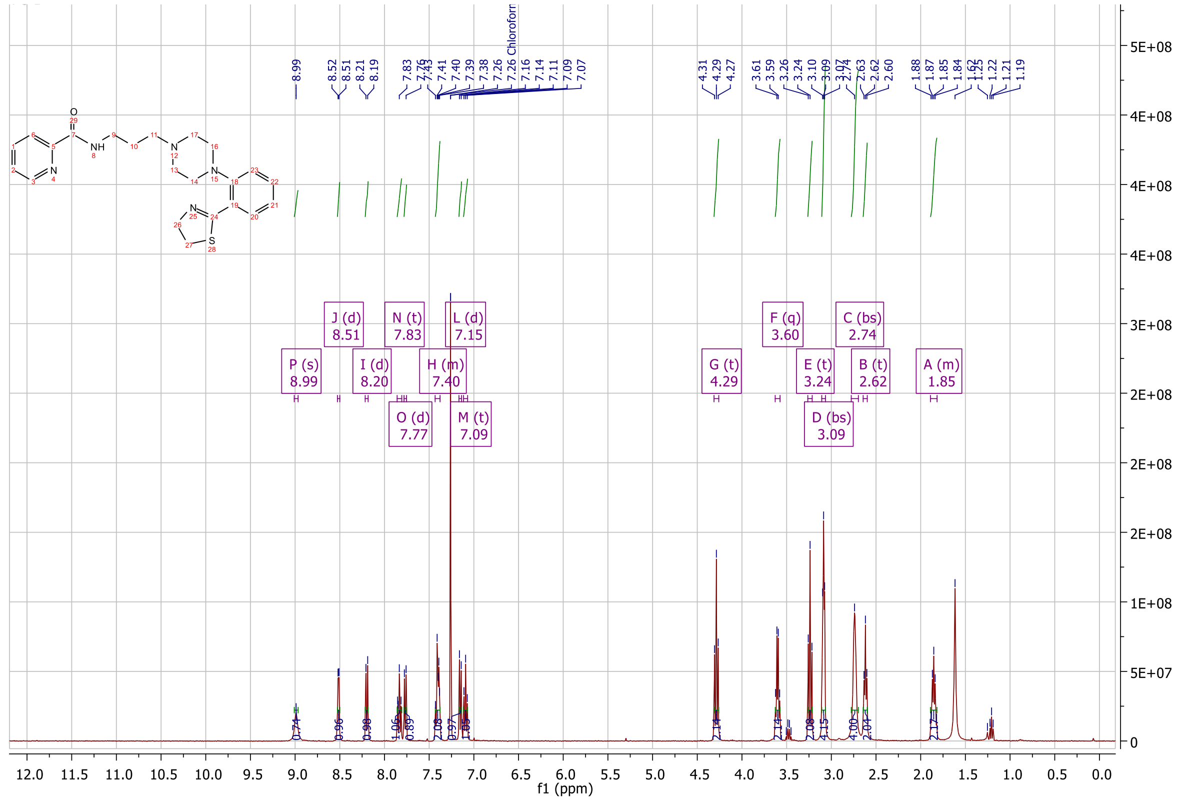

Figure S1: ^{1}H-NMR of N-(3-(4-(2-(4,5-dihydrothiazol-2-yl)phenyl)piperazin-1-yl)propyl)picolinamide (**FG-1**).

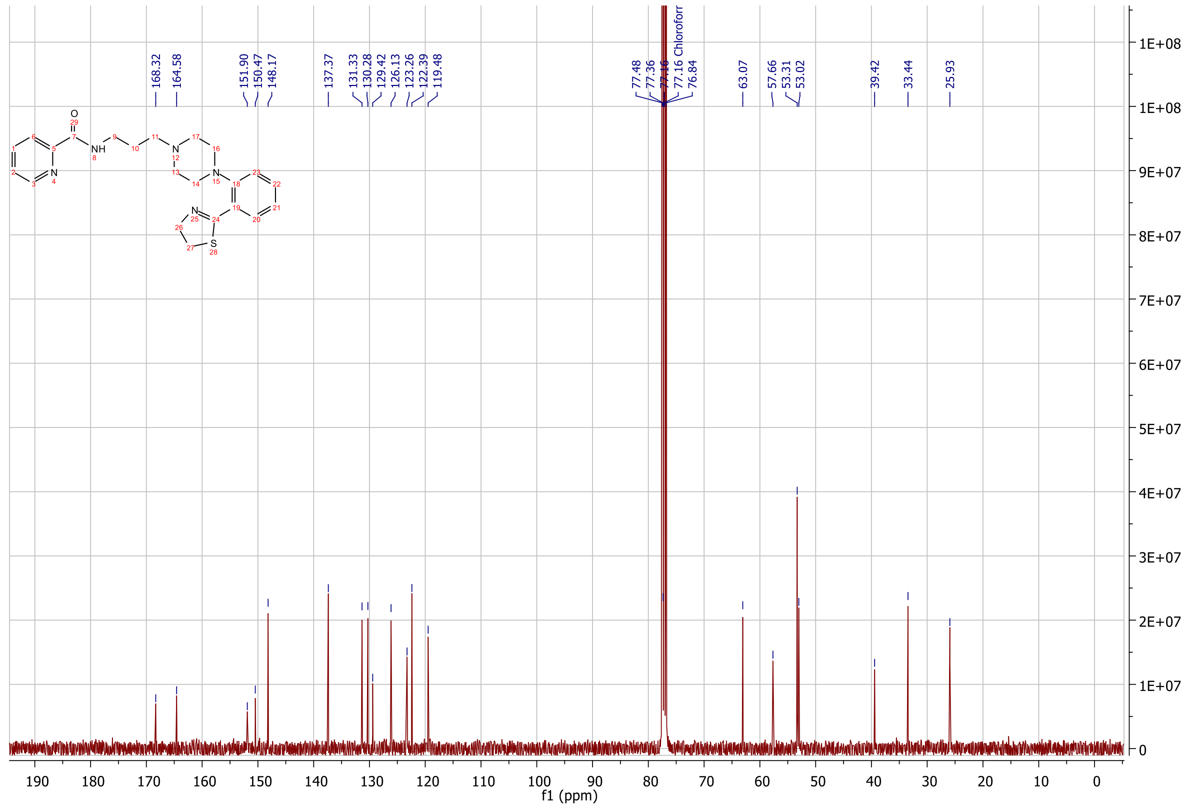

Figure S2: ^{13}C-NMR of N-(3-(4-(2-(4,5-dihydrothiazol-2-yl)phenyl)piperazin-1-yl)propyl)picolinamide (**FG-1**).

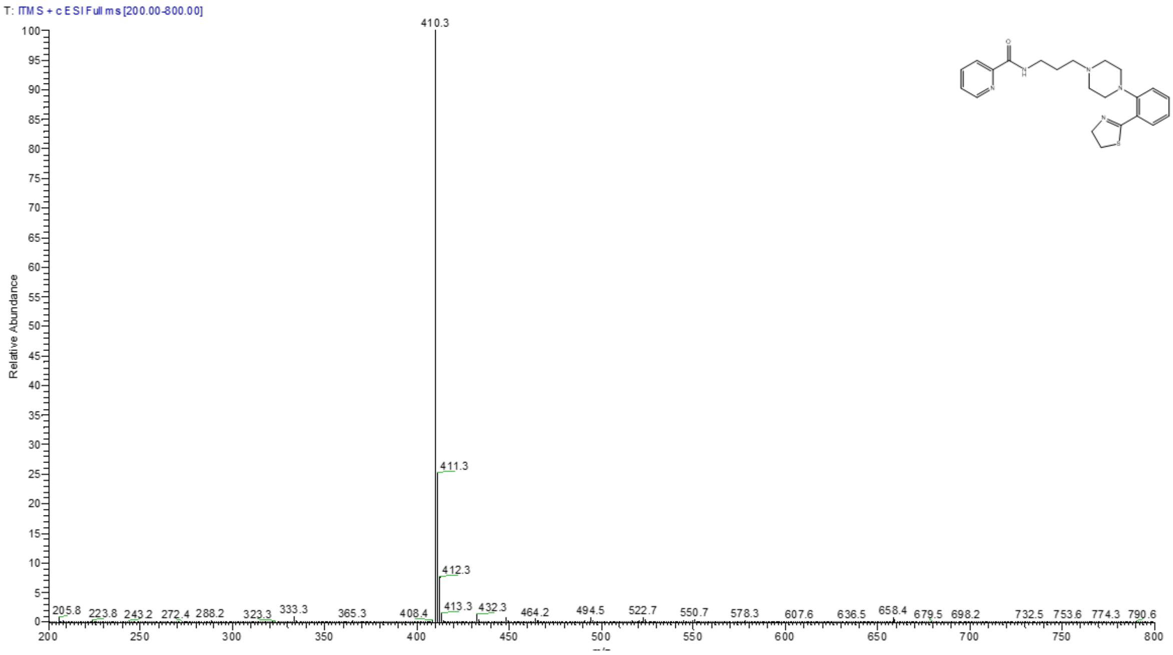

Figure S3: MS of N-(3-(4-(2-(4,5-dihydrothiazol-2-yl)phenyl)piperazin-1-yl)propyl)picolinamide (**FG-1**).

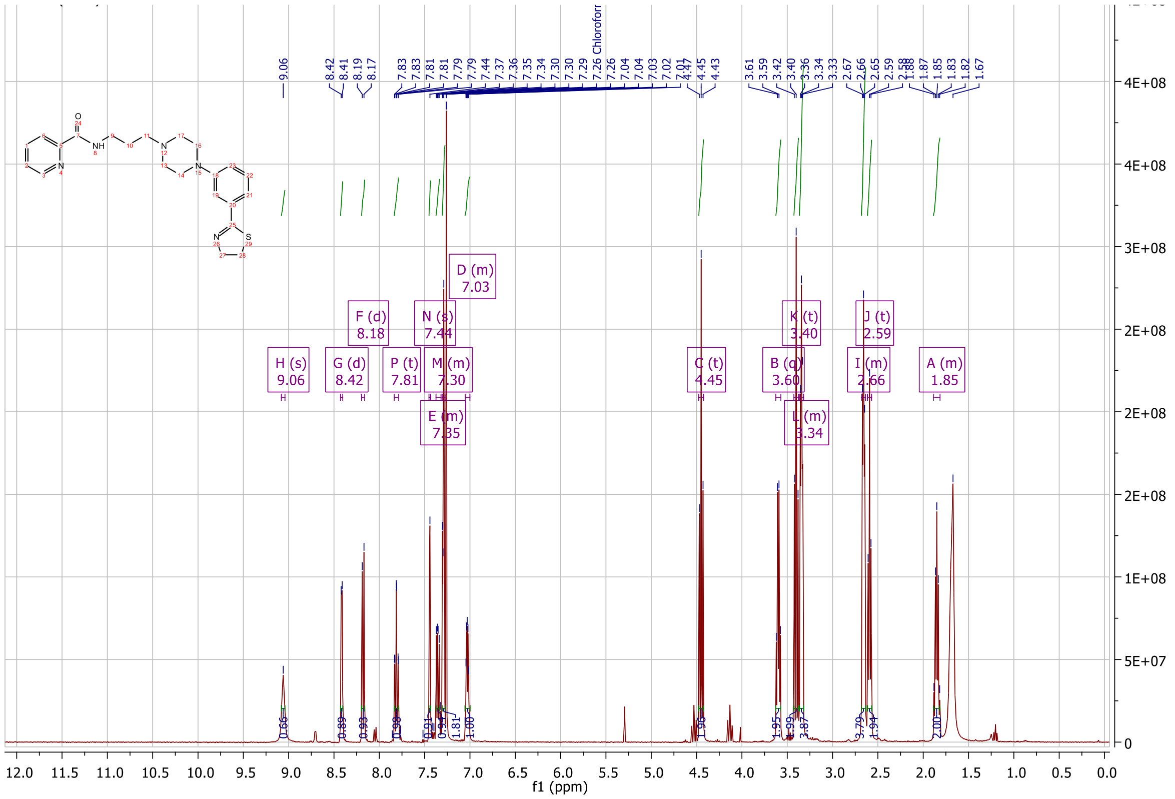

Figure S4: ^{1}H-NMR of N-(3-(4-(3-(4,5-dihydrothiazol-2-yl)phenyl)piperazin-1-yl)propyl)picolinamide (**FG-2**).

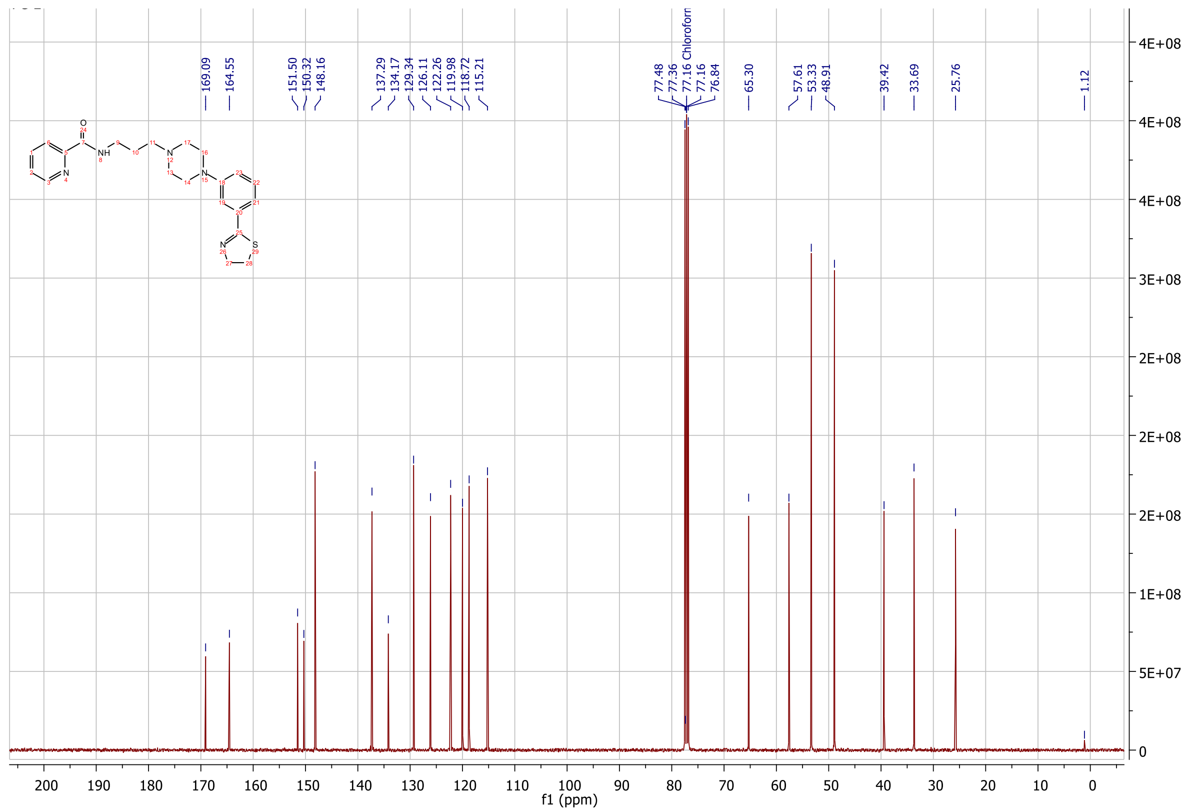

Figure S5: ^{13}C-NMR of N-(3-(4-(3-(4,5-dihydrothiazol-2-yl)phenyl)piperazin-1-yl)propyl)picolinamide (**FG-2**).

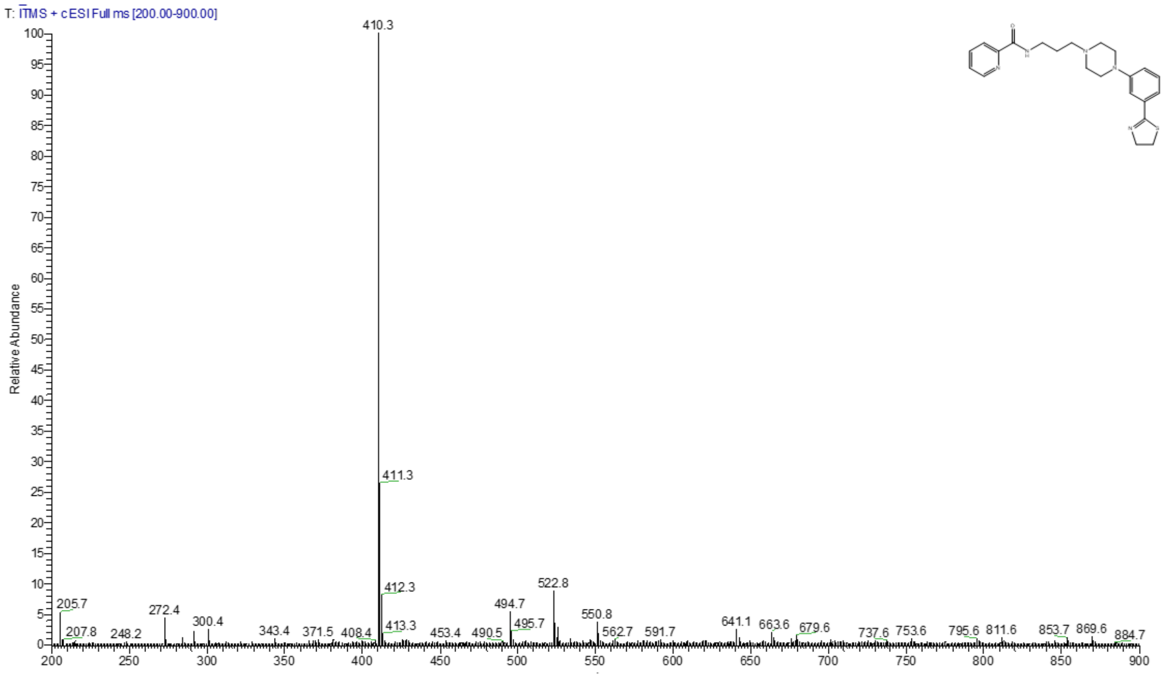

Figure S6: MS of N-(3-(4-(3-(4,5-dihydrothiazol-2-yl)phenyl)piperazin-1-yl)propyl)picolinamide (**FG-2**).

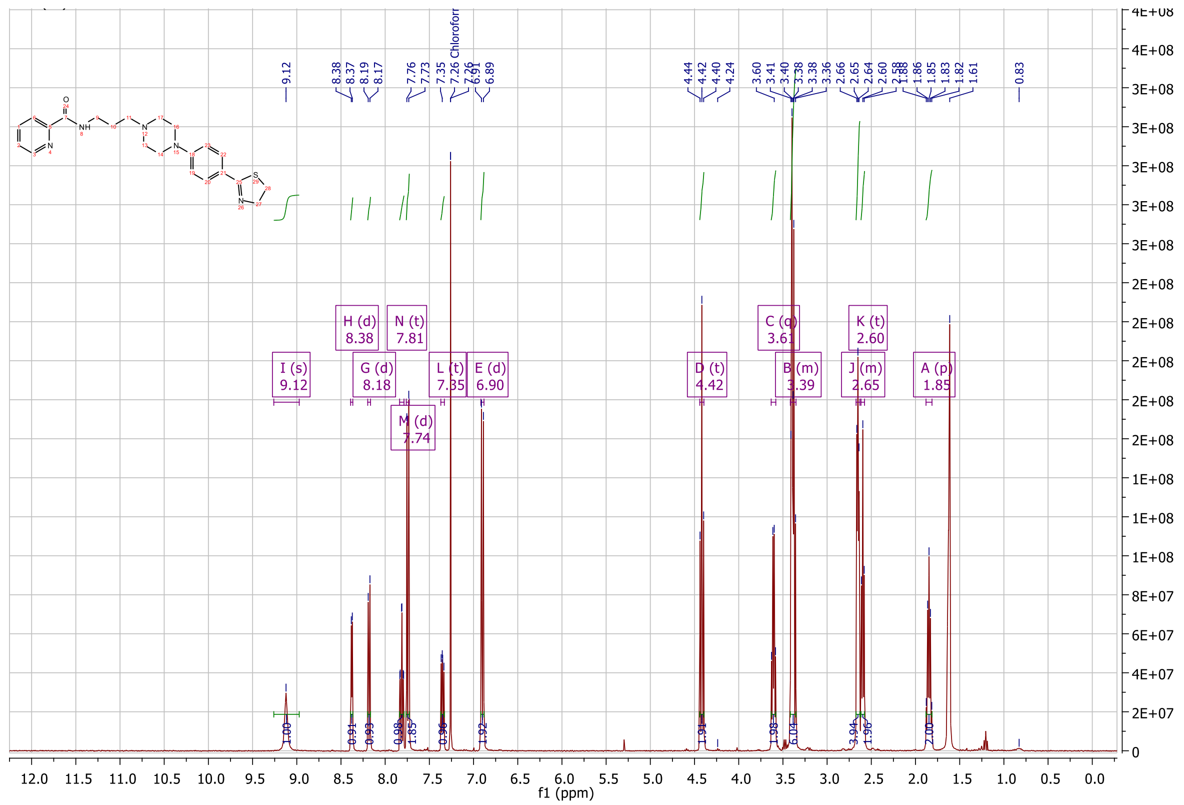

Figure S7: ^{1}H-NMR of N-(3-(4-(4-(4,5-dihydrothiazol-2-yl)phenyl)piperazin-1-yl)propyl)picolinamide (**FG-3**).

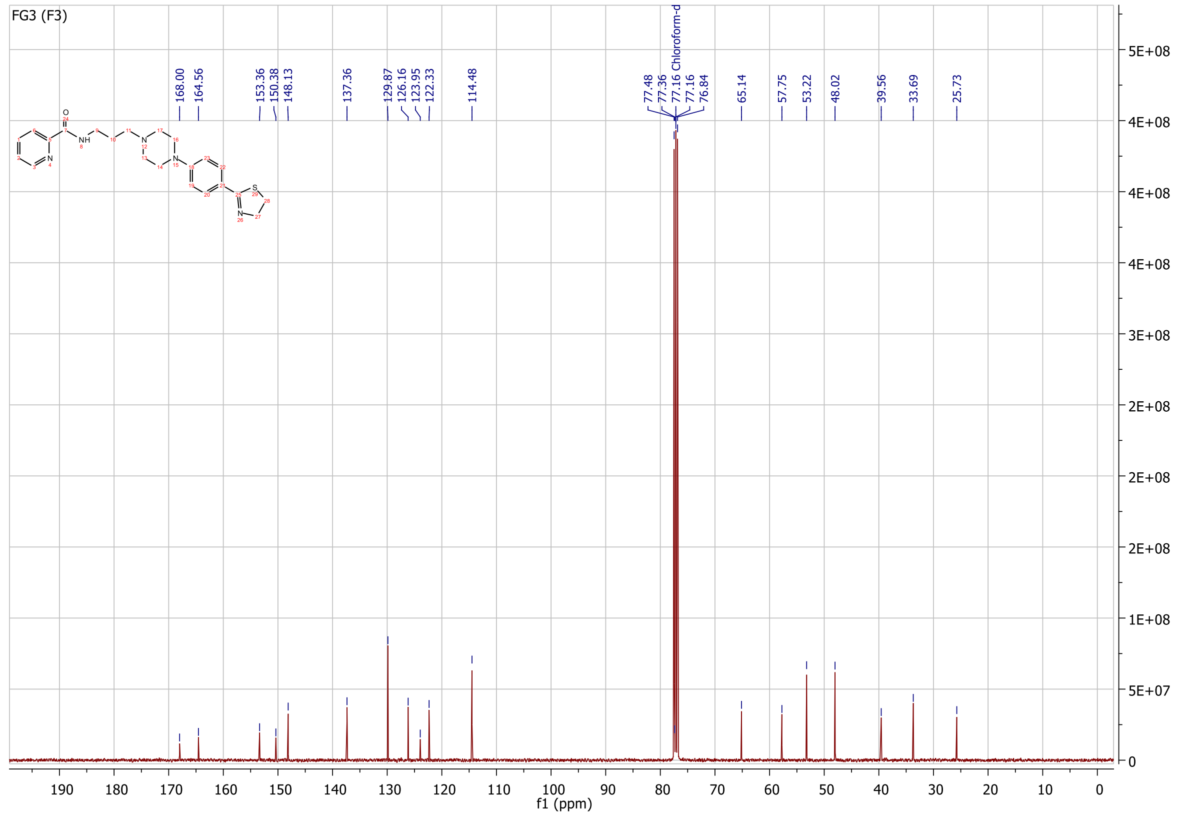

Figure S8: ^{13}C-NMR of N-(3-(4-(4-(4,5-dihydrothiazol-2-yl)phenyl)piperazin-1-yl)propyl)picolinamide (**FG-3**).

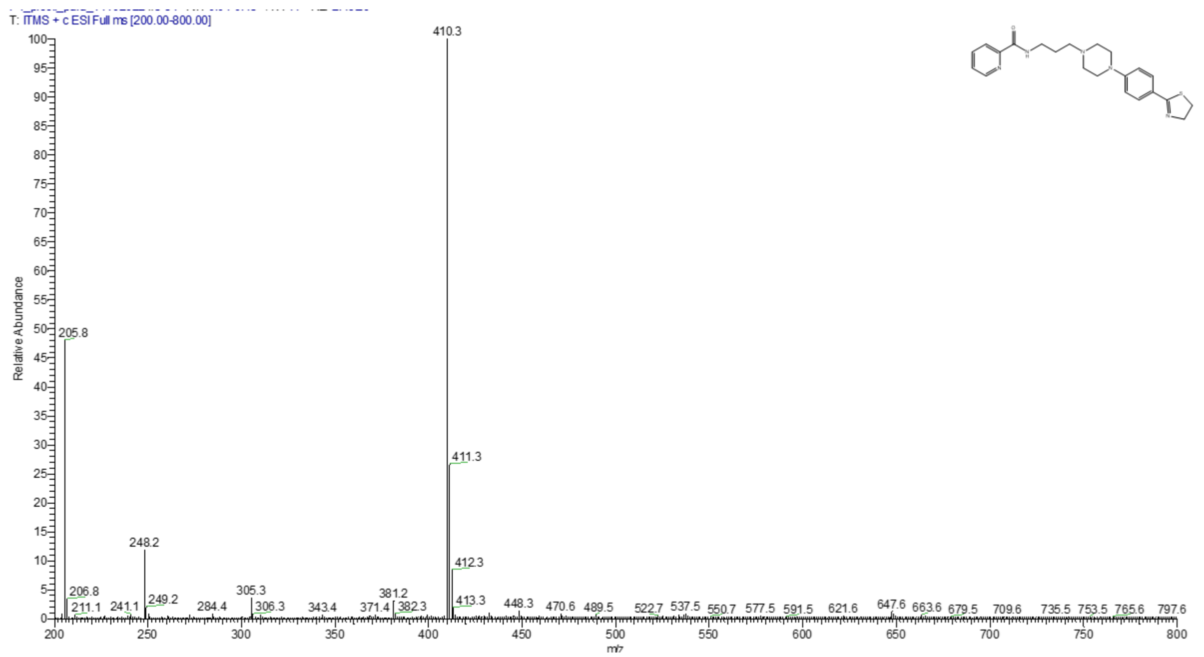

Figure S9: MS of N-(3-(4-(4-(4,5-dihydrothiazol-2-yl)phenyl)piperazin-1-yl)propyl)picolinamide (**FG-3**).

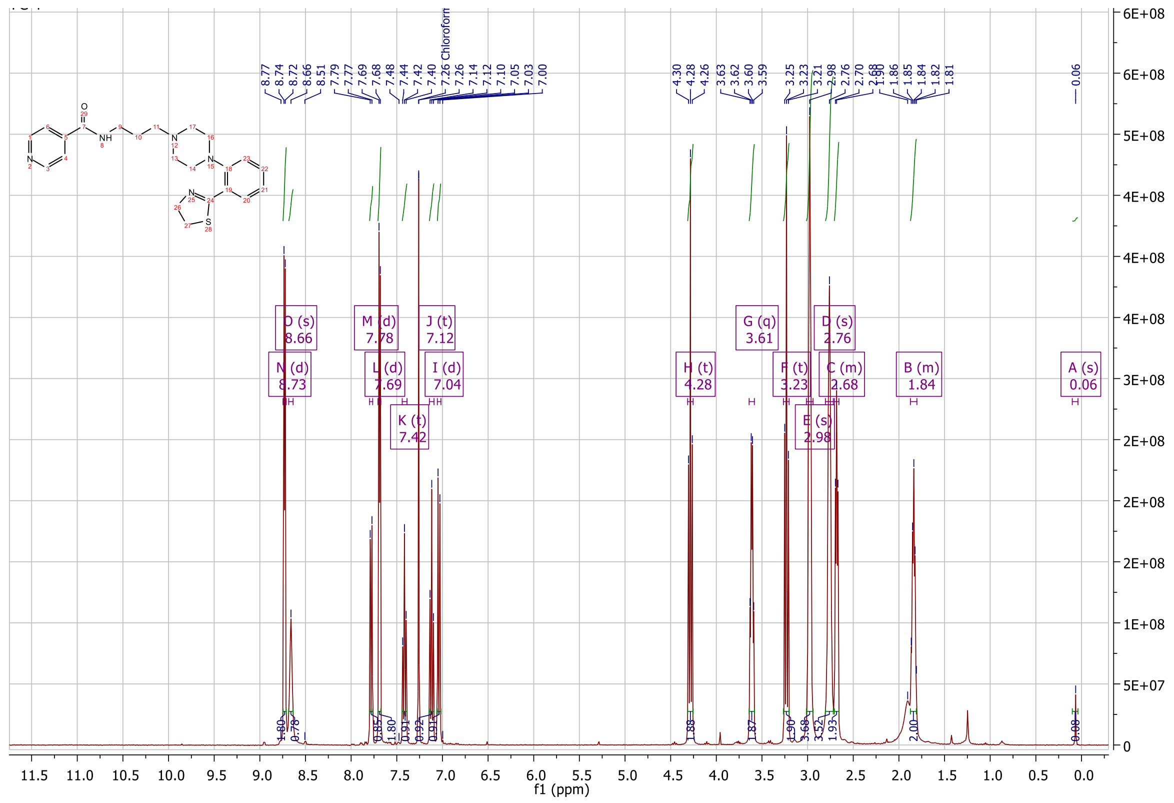

Figure S10: ^{1}H-NMR of N-(3-(4-(2-(4,5-dihydrothiazol-2-yl)phenyl)piperazin-1-yl)propyl)isonicotinamide (**FG-4**).

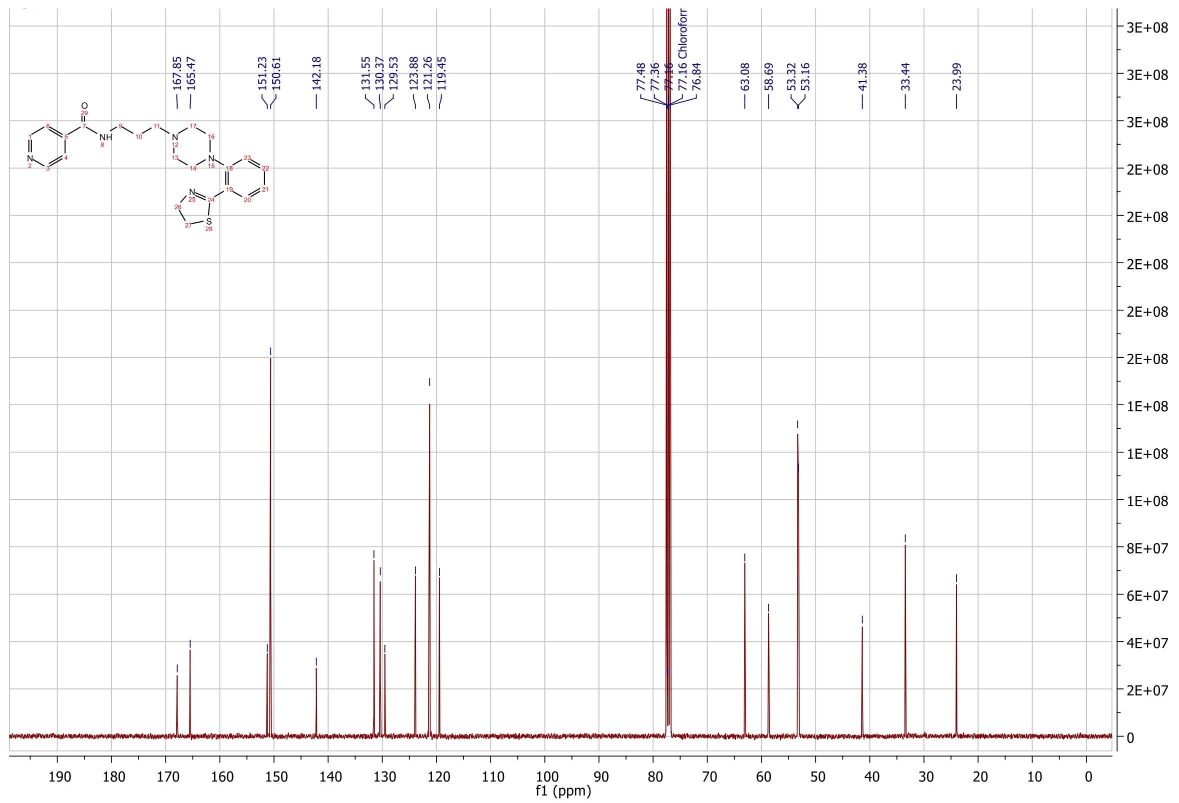

Figure S11: ^{13}C-NMR of N-(3-(4-(2-(4,5-dihydrothiazol-2-yl)phenyl)piperazin-1-yl)propyl)isonicotinamide (**FG-4**).

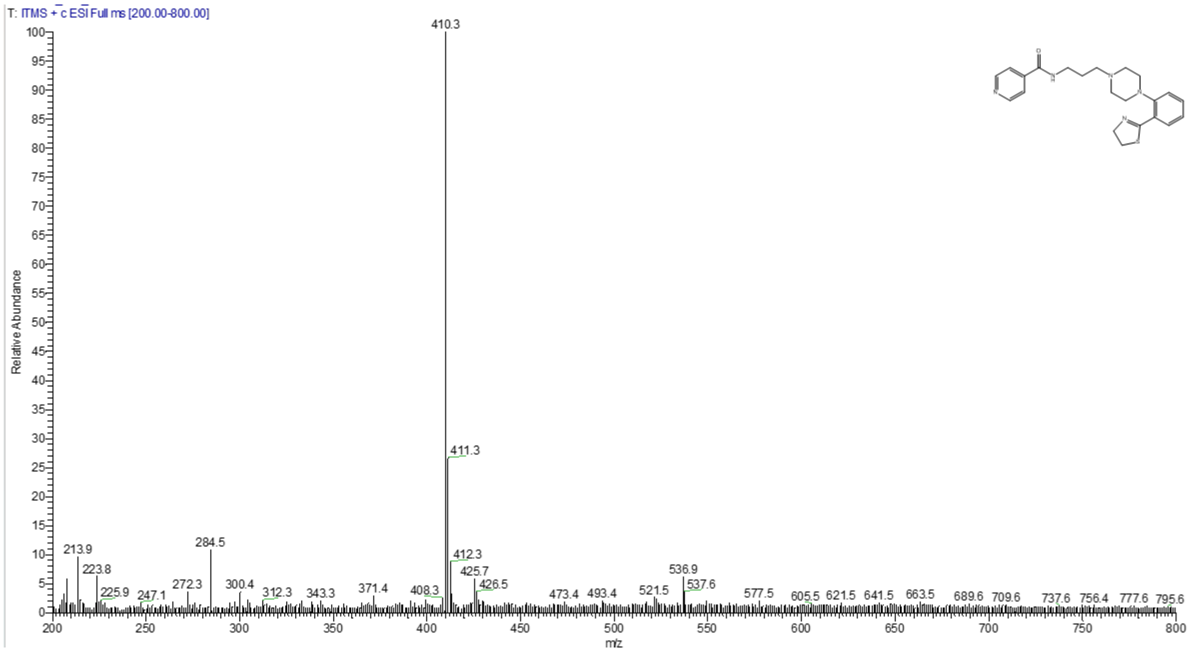

Figure S12:MS of N-(3-(4-(2-(4,5-dihydrothiazol-2-yl)phenyl)piperazin-1-yl)propyl)isonicotinamide (**FG-4**).

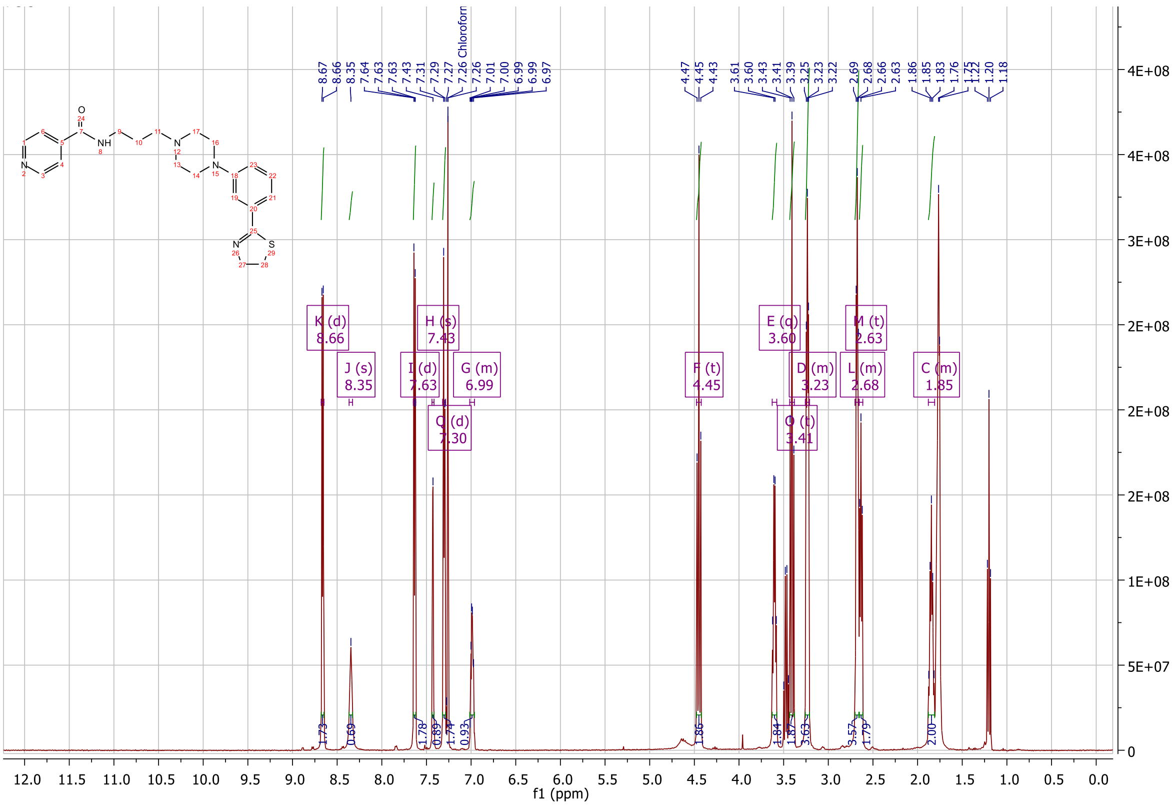

Figure S13: ^{1}H-NMR of N-(3-(4-(3-(4,5-dihydrothiazol-2-yl)phenyl)piperazin-1-yl)propyl)isonicotinamide (**FG-5**).

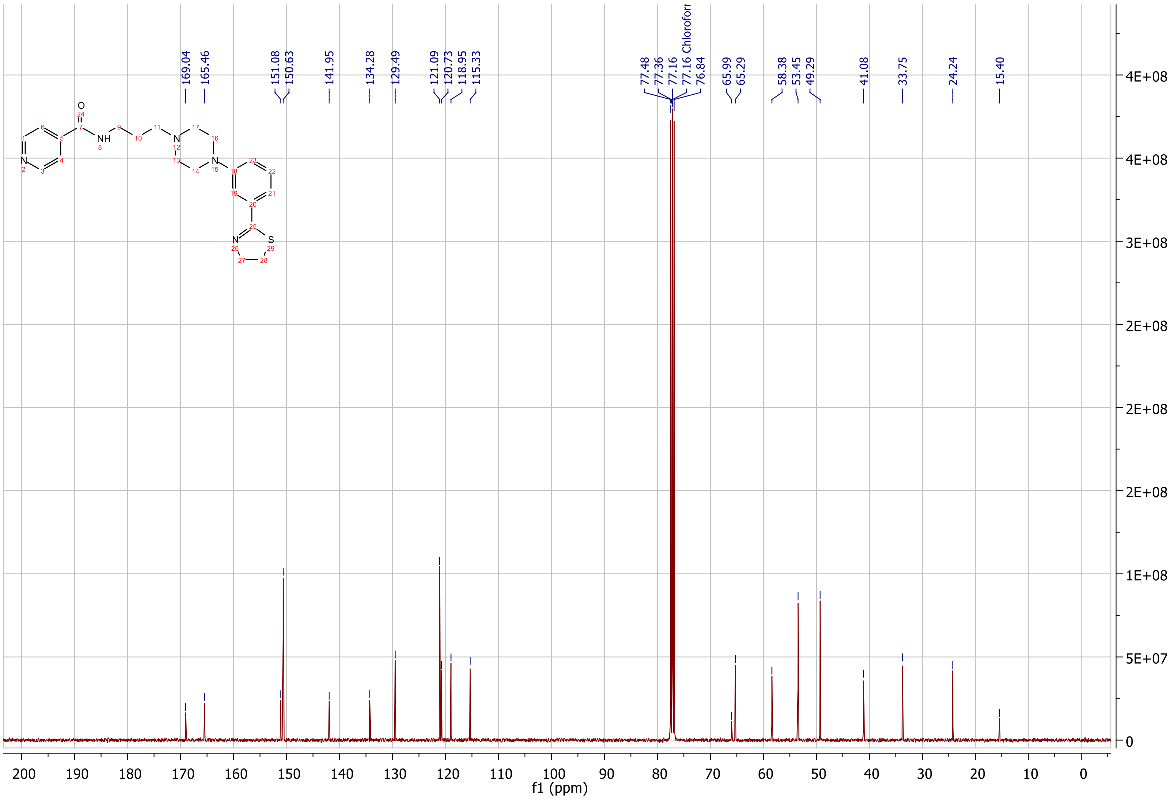

Figure S14: ^{13}C-NMR of N-(3-(4-(3-(4,5-dihydrothiazol-2-yl)phenyl)piperazin-1-yl)propyl)isonicotinamide (**FG-5**).

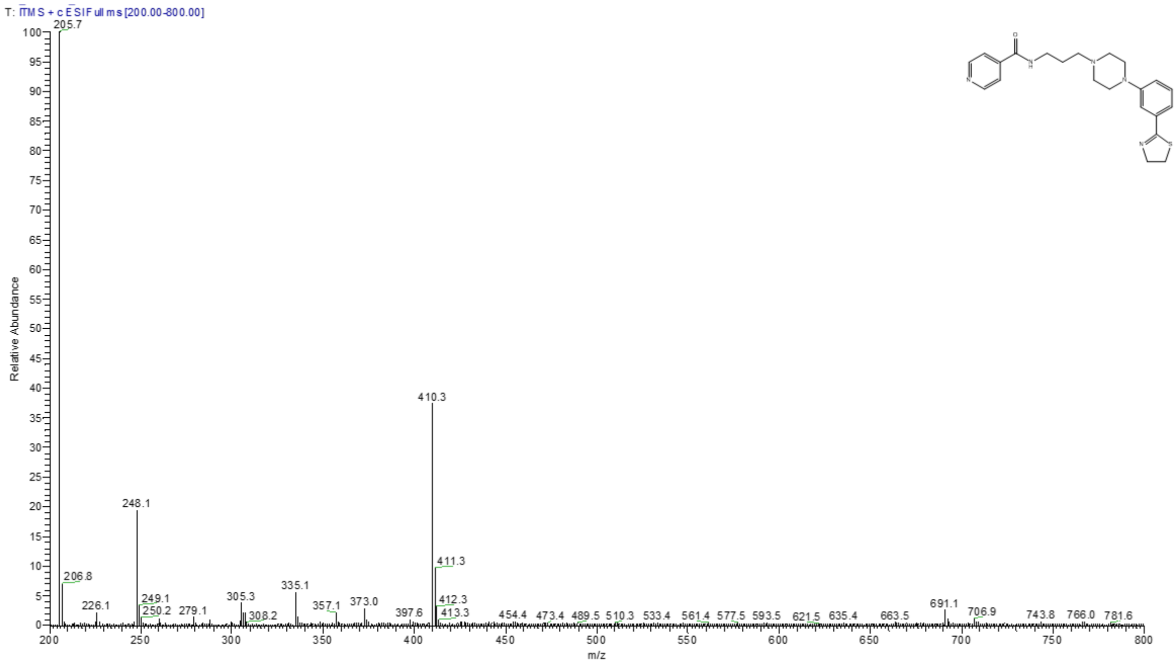

Figure S15: MS of N-(3-(4-(3-(4,5-dihydrothiazol-2-yl)phenyl)piperazin-1-yl)propyl)isonicotinamide (**FG-5**).

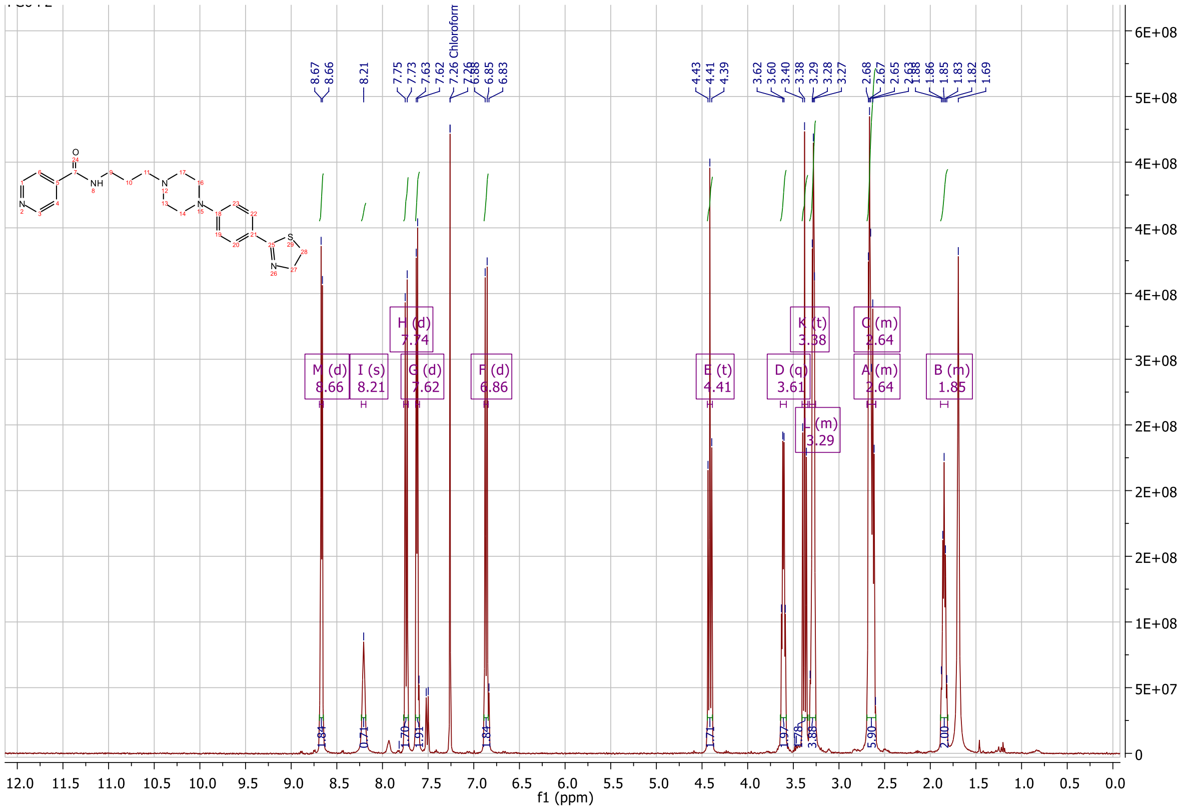

Figure S16: ^{1}H-NMR N-(3-(4-(4-(4,5-dihydrothiazol-2-yl)phenyl)piperazin-1-yl)propyl)isonicotinamide (**FG-6**).

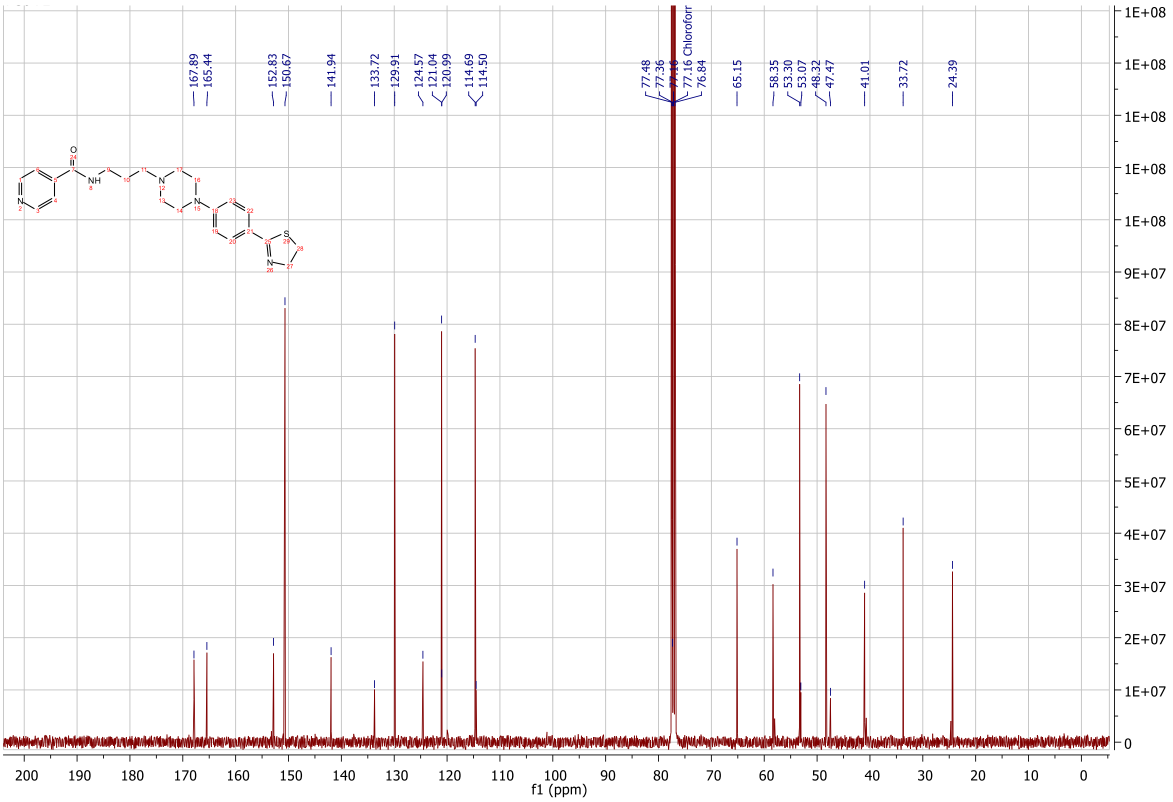

Figure S17: ^{13}C-NMR N-(3-(4-(4-(4,5-dihydrothiazol-2-yl)phenyl)piperazin-1-yl)propyl)isonicotinamide (**FG-6**).

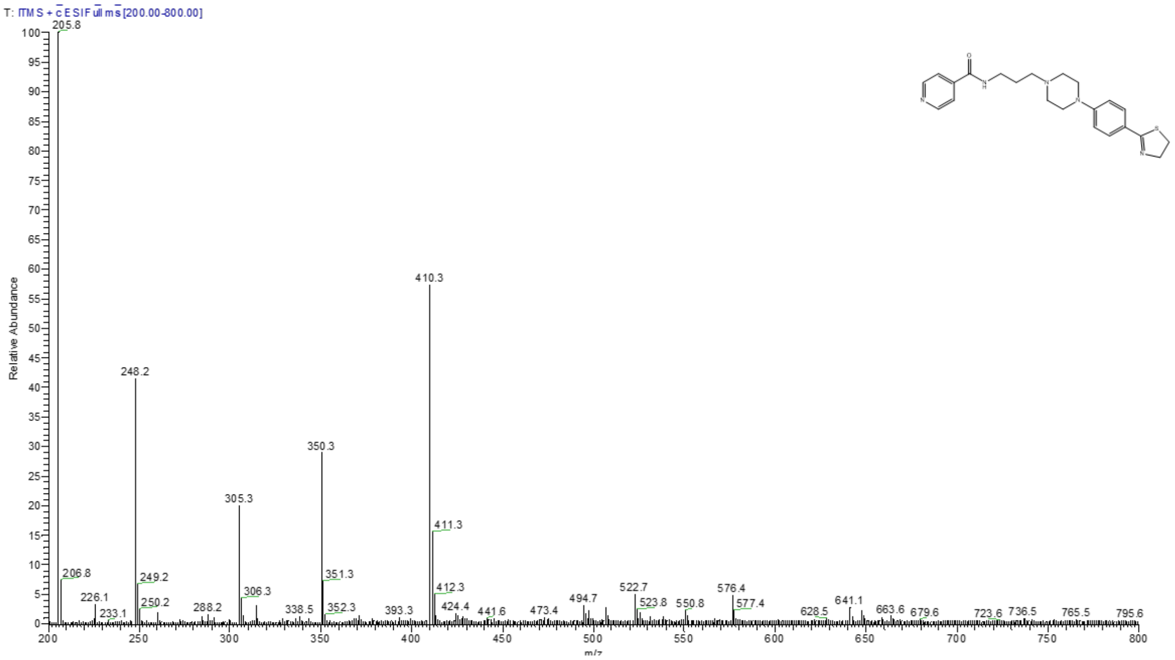

Figure S18: MS of N-(3-(4-(4-(4,5-dihydrothiazol-2-yl)phenyl)piperazin-1-yl)propyl)isonicotinamide (**FG-6**).

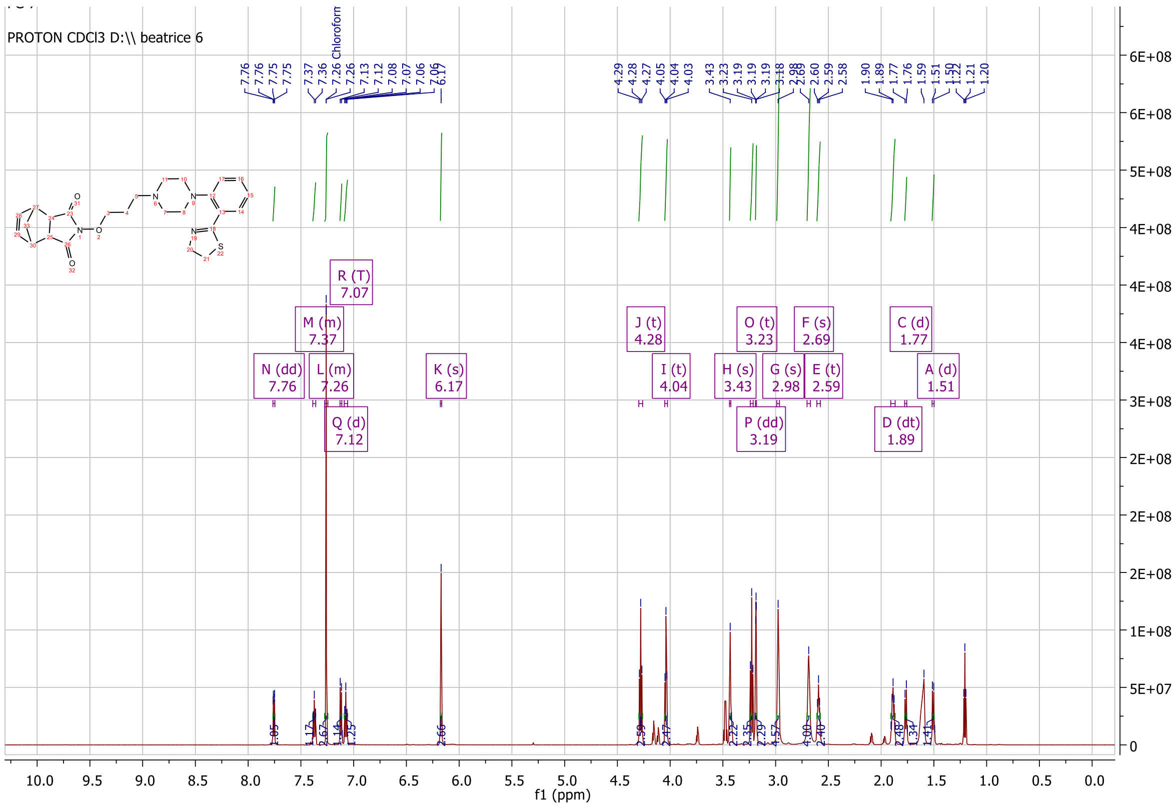

Figure S19: ^{1}H-NMR of 2-(3-(4-(2-(4,5-dihydrothiazol-2-yl)phenyl)piperazin-1-yl)propoxy)-3a,4,7,7a-tetrahydro-1H-4,7-methanoisoindole-1,3(2H)-dione (**FG-7**).

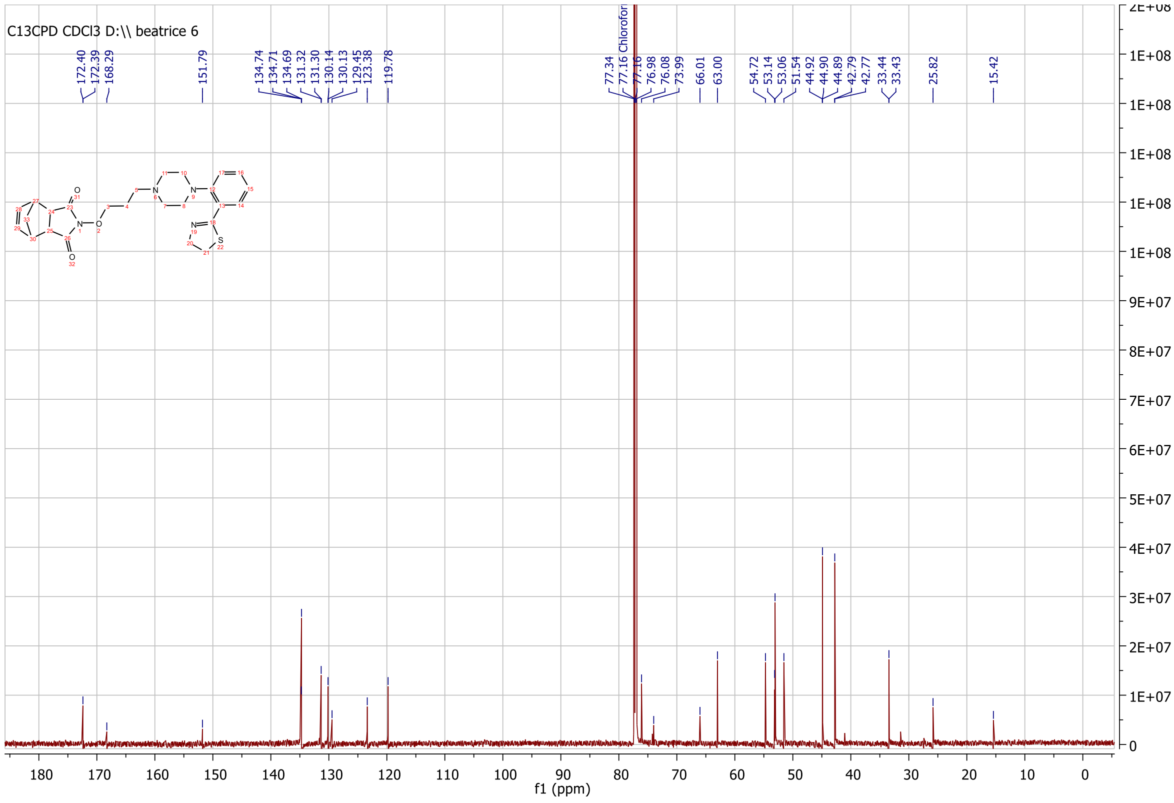

Figure S20: ^{13}C-NMR of 2-(3-(4-(2-(4,5-dihydrothiazol-2-yl)phenyl)piperazin-1-yl)propoxy)-3a,4,7,7a-tetrahydro-1H-4,7-methanoisoindole-1,3(2H)-dione (**FG-7**).

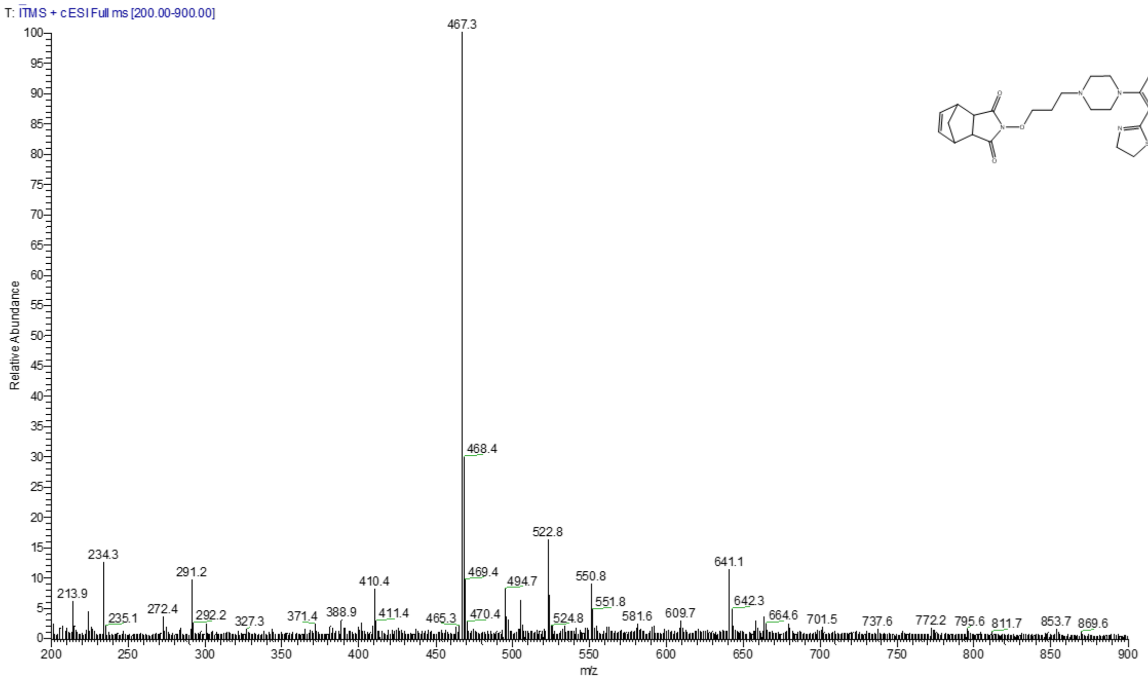

Figure S21: MS of 2-(3-(4-(2-(4,5-dihydrothiazol-2-yl)phenyl)piperazin-1-yl)propoxy)-3a,4,7,7a-tetrahydro-1H-4,7-methanoisoindole-1,3(2H)-dione (**FG-7**).

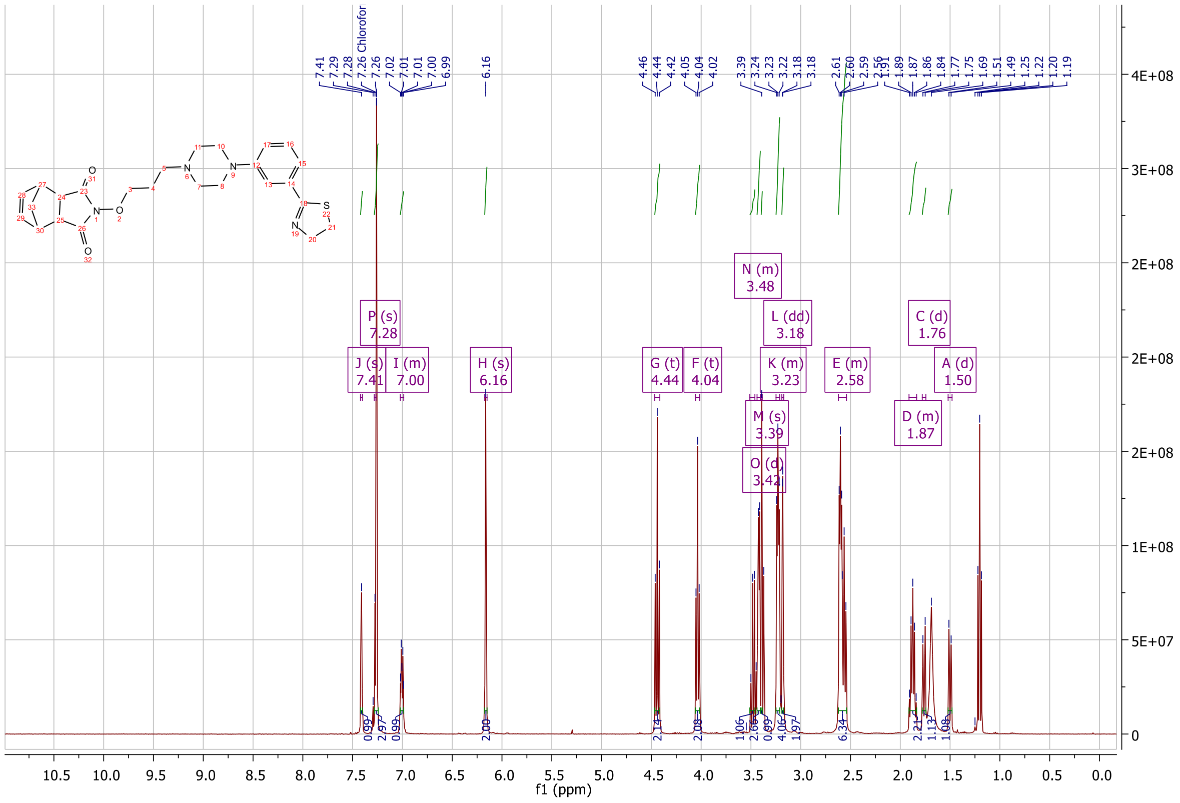

Figure S22: ^{1}H-NMR of 2-(3-(4-(3-(4,5-dihydrothiazol-2-yl)phenyl)piperazin-1-yl)propoxy)-3a,4,7,7a-tetrahydro-1H-4,7-methanoisoindole-1,3(2H)-dione (**FG-8**).

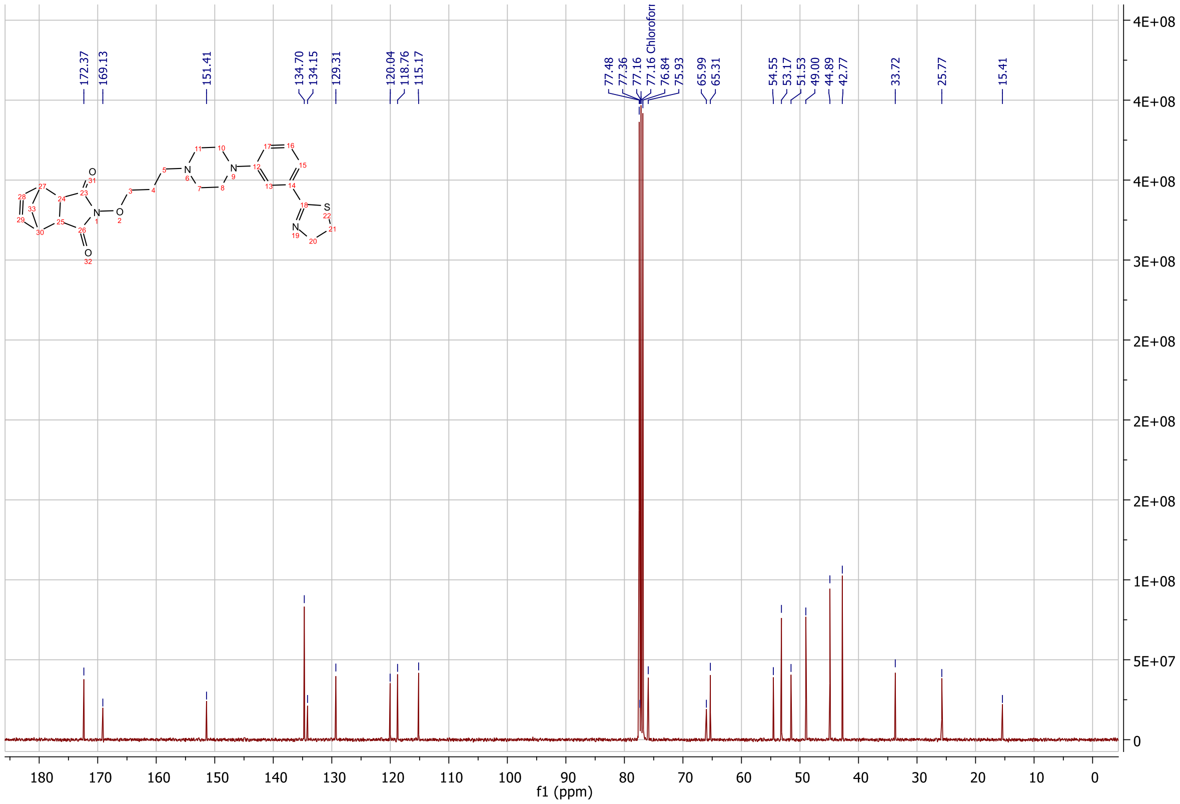

Figure S23: ^{13}C-NMR of 2-(3-(4-(3-(4,5-dihydrothiazol-2-yl)phenyl)piperazin-1-yl)propoxy)-3a,4,7,7a-tetrahydro-1H-4,7-methanoisoindole-1,3(2H)-dione (**FG-8**).

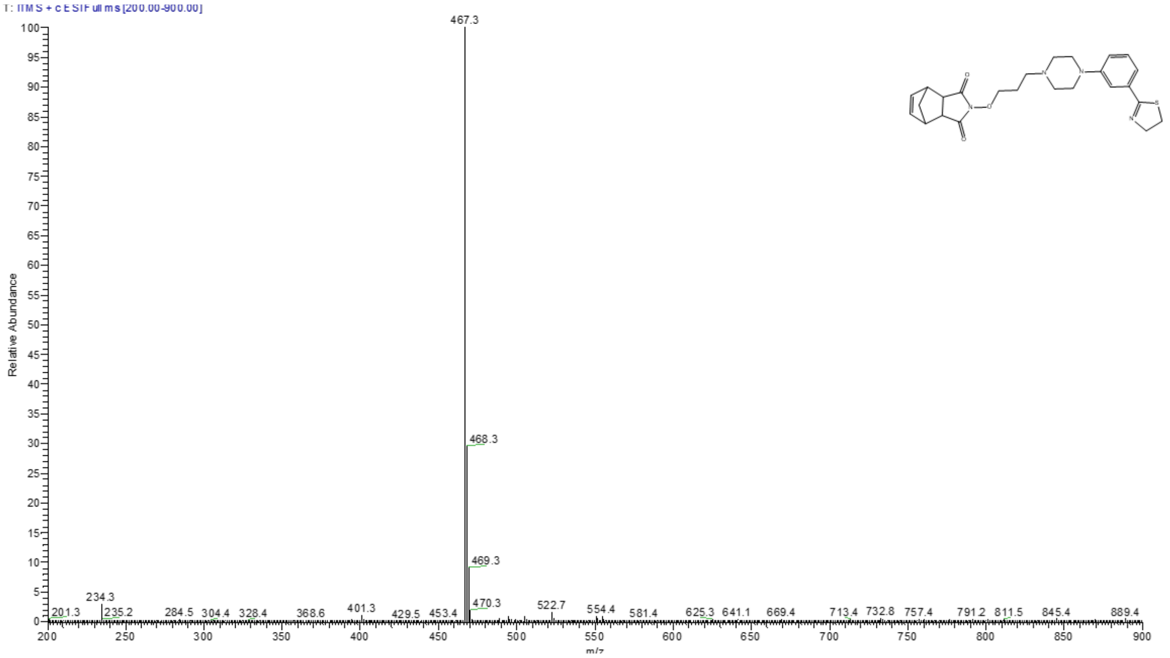

Figure S24: MS of 2-(3-(4-(3-(4,5-dihydrothiazol-2-yl)phenyl)piperazin-1-yl)propoxy)-3a,4,7,7a-tetrahydro-1H-4,7-methanoisoindole-1,3(2H)-dione (**FG-8**).

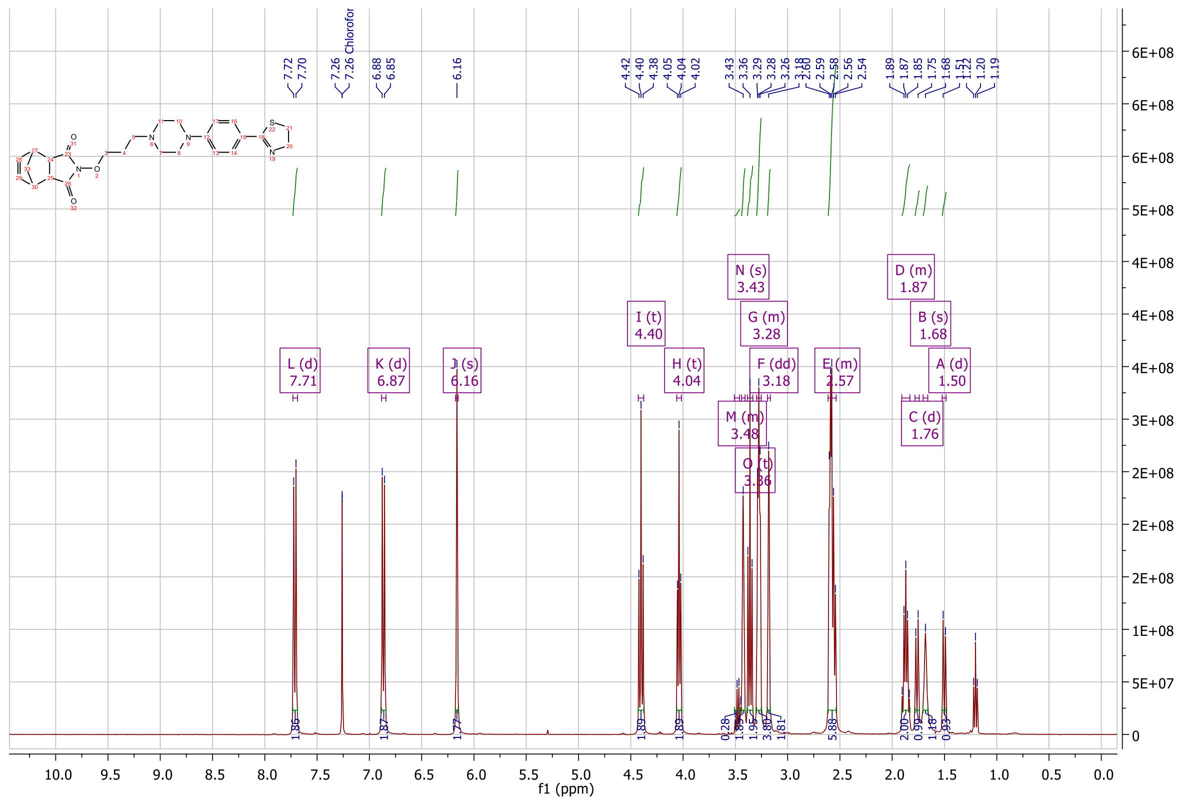

Figure S25: ^{1}H-NMR of 2-(3-(4-(4-(4,5-dihydrothiazol-2-yl)phenyl)piperazin-1-yl)propoxy)-3a,4,7,7a-tetrahydro-1H-4,7-methanoisoindole-1,3(2H)-dione (**FG-9**).

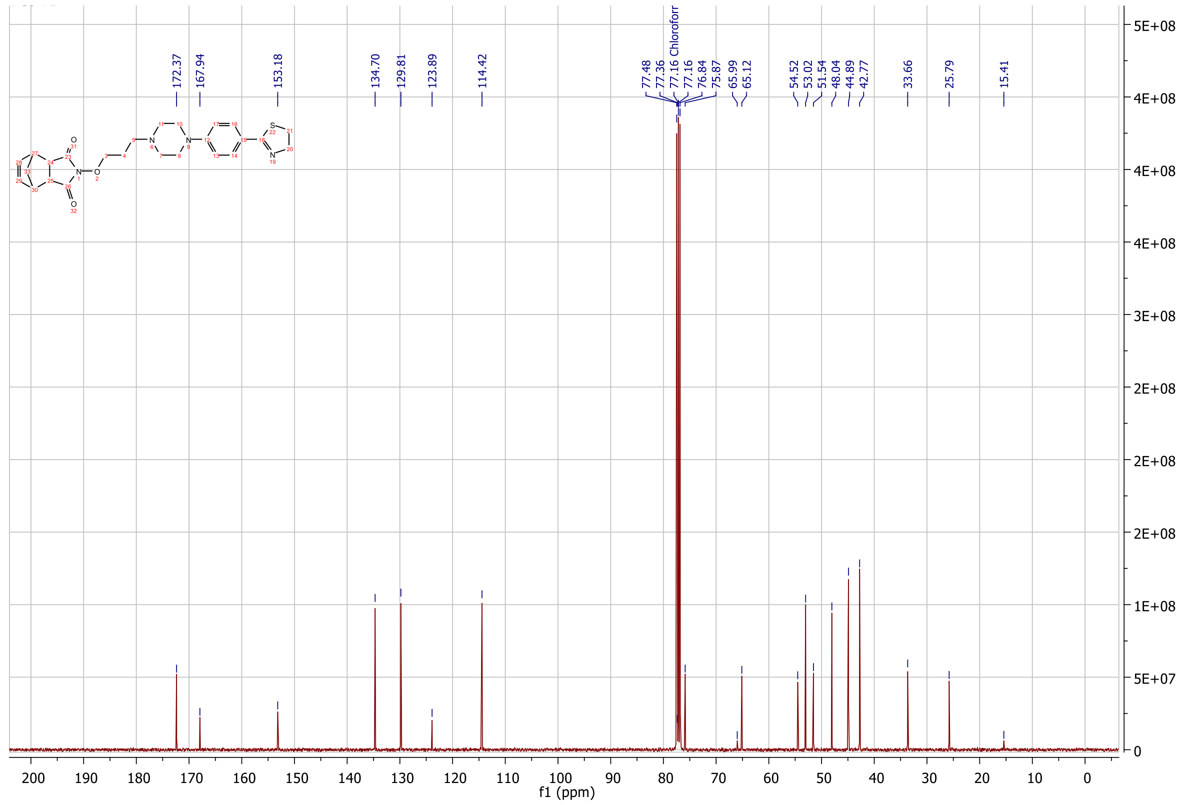

Figure S26: ^{13}C-NMR of 2-(3-(4-(4-(4,5-dihydrothiazol-2-yl)phenyl)piperazin-1-yl)propoxy)-3a,4,7,7a-tetrahydro-1H-4,7-methanoisoindole-1,3(2H)-dione (**FG-9**).

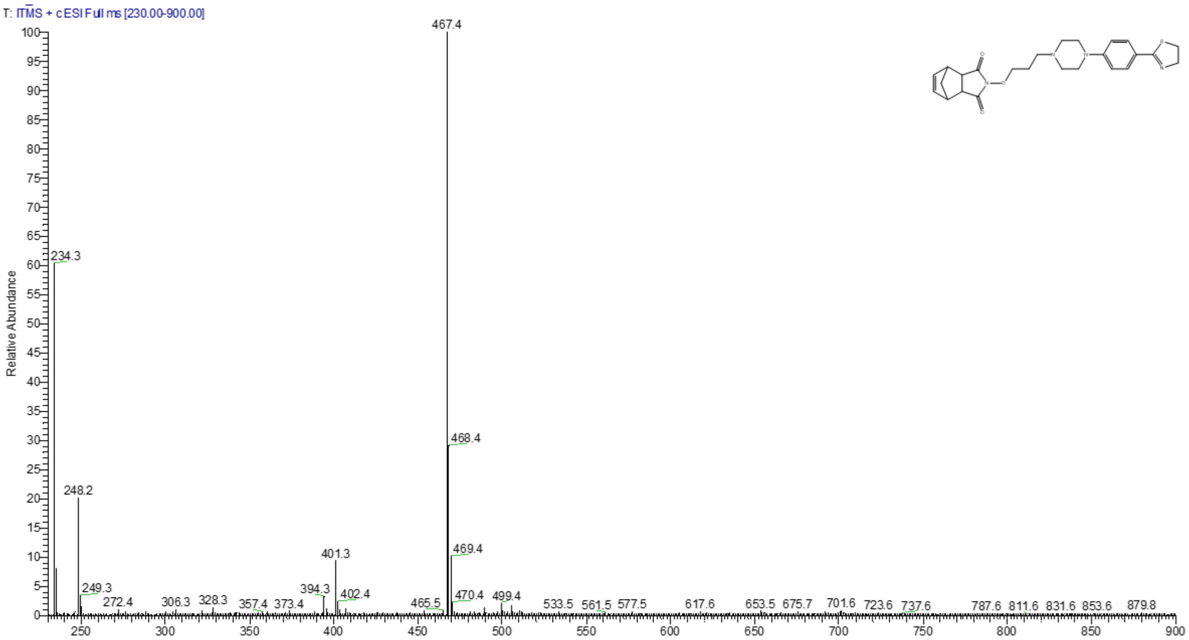

Figure S27: MS of 2-(3-(4-(4-(4,5-dihydrothiazol-2-yl)phenyl)piperazin-1-yl)propoxy)-3a,4,7,7a-tetrahydro-1H-4,7-methanoisoindole-1,3(2H)-dione (**FG-9**).

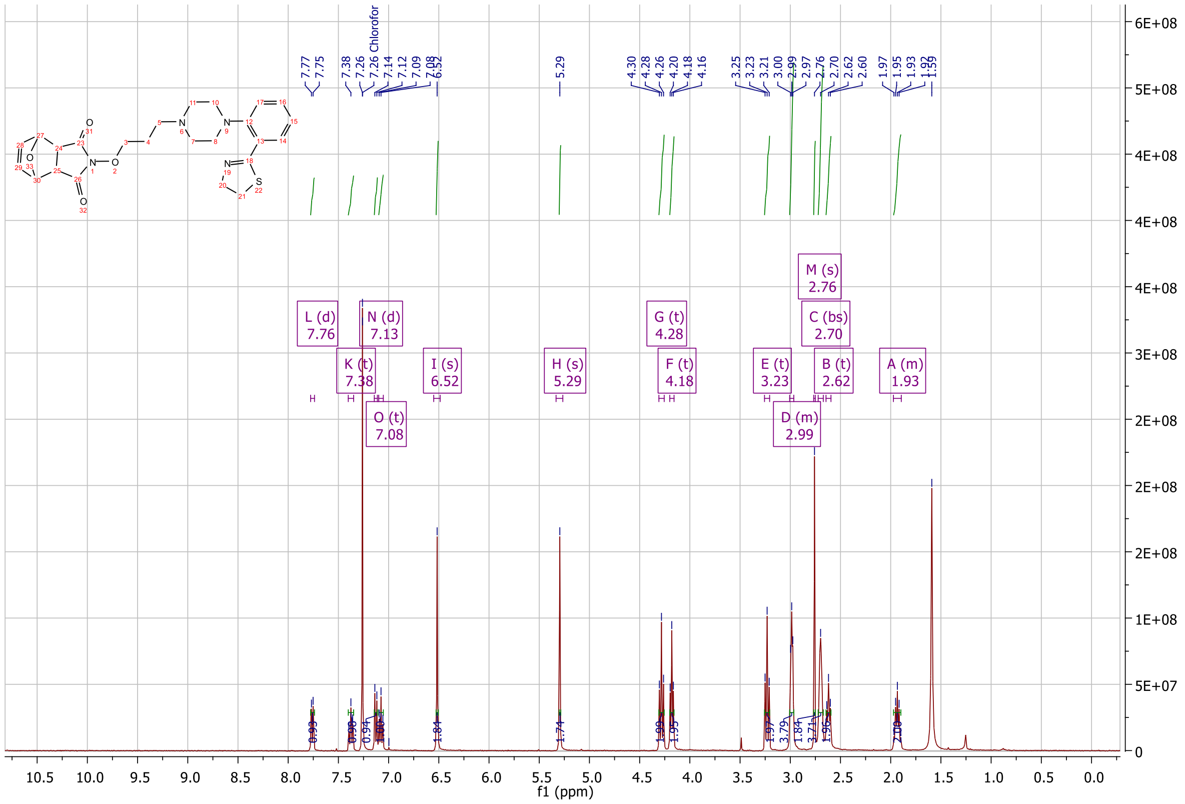

Figure S28: ^{1}H-NMR of 2-(3-(4-(2-(4,5-dihydrothiazol-2-yl)phenyl)piperazin-1-yl)propoxy)-3a,4,7,7a-tetrahydro-1H-4,7-epoxyisoindole-1,3(2H)-dione (**FG-10**).

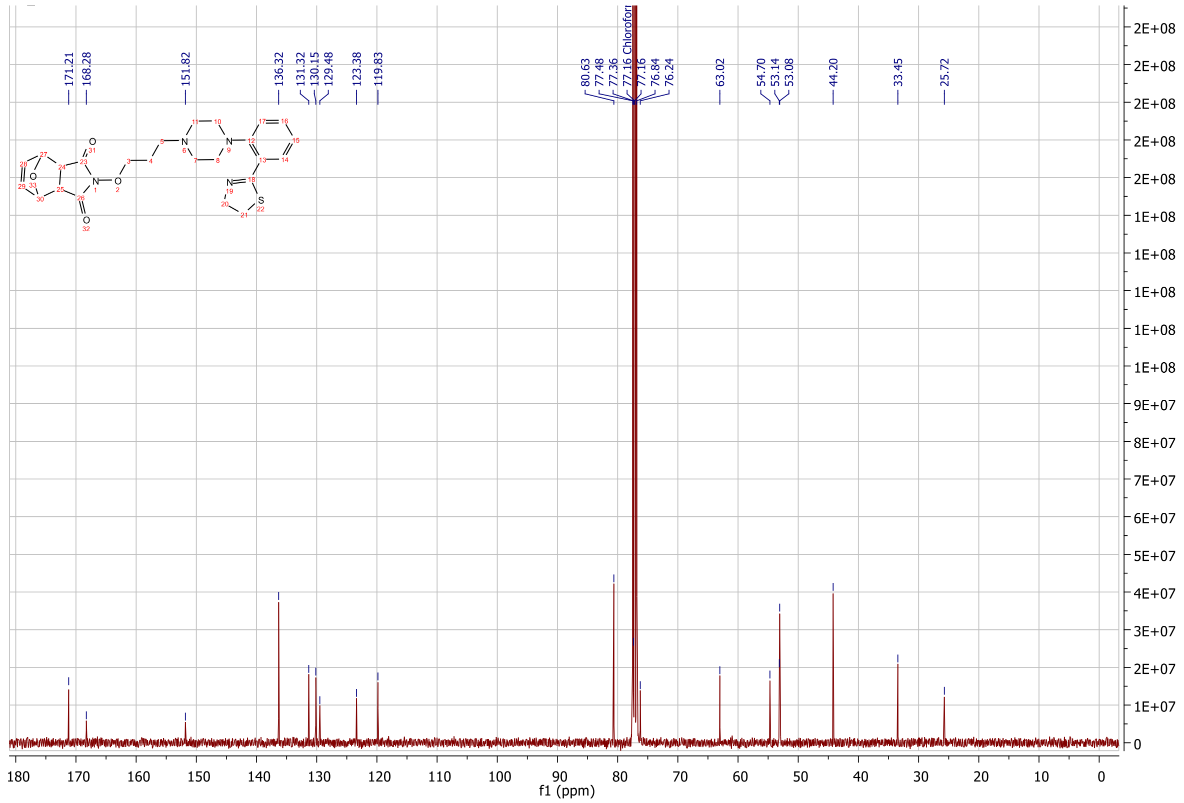

Figure S29: ^{13}C-NMR of 2-(3-(4-(2-(4,5-dihydrothiazol-2-yl)phenyl)piperazin-1-yl)propoxy)-3a,4,7,7a-tetrahydro-1H-4,7-epoxyisoindole-1,3(2H)-dione (**FG-10**).

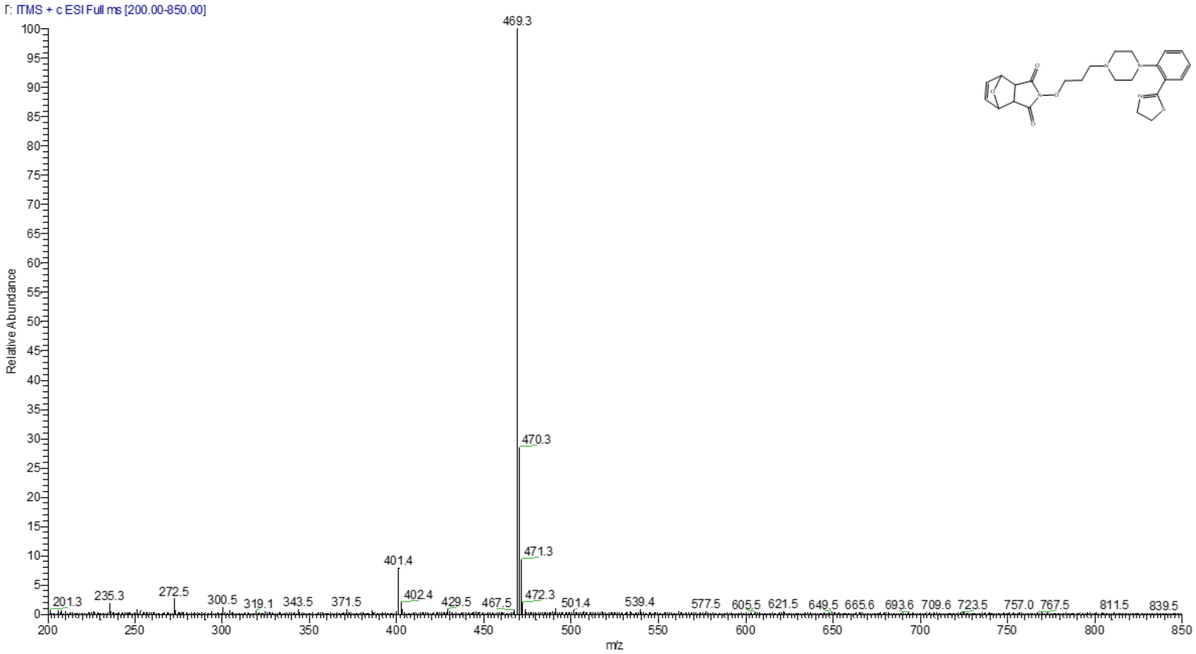

Figure S30: MS of 2-(3-(4-(2-(4,5-dihydrothiazol-2-yl)phenyl)piperazin-1-yl)propoxy)-3a,4,7,7a-tetrahydro-1H-4,7-epoxyisoindole-1,3(2H)-dione (**FG-10**).

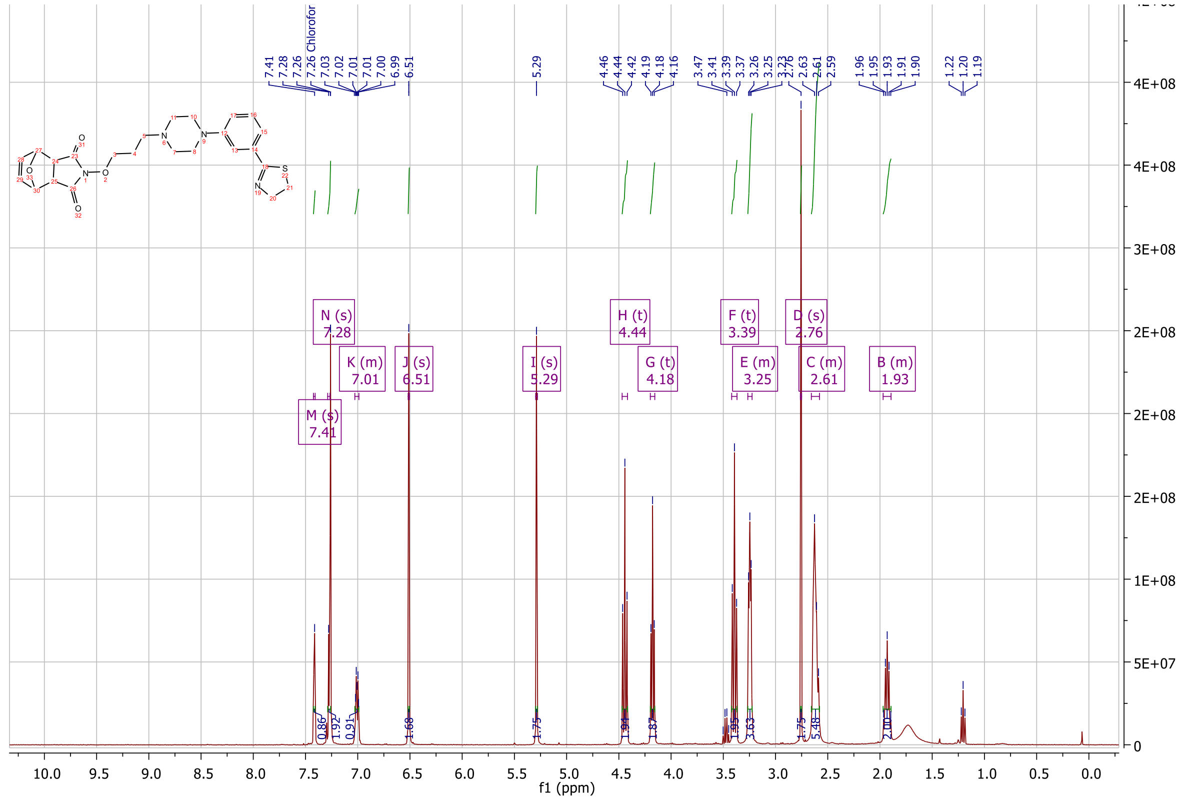

Figure S31: ^{1}H-NMR of 2-(3-(4-(3-(4,5-dihydrothiazol-2-yl)phenyl)piperazin-1-yl)propoxy)-3a,4,7,7a-tetrahydro-1H-4,7-epoxyisoindole-1,3(2H)-dione (**FG-11**).

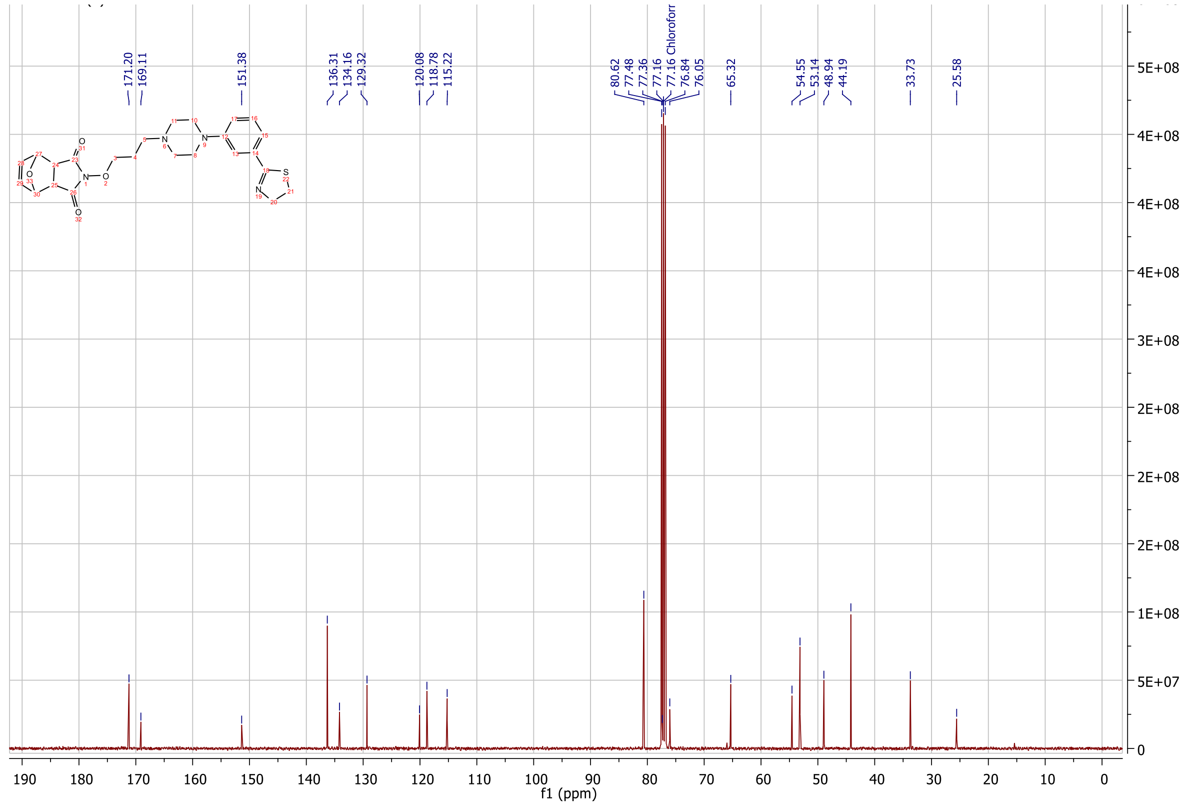

Figure S32: ^{13}C-NMR of 2-(3-(4-(3-(4,5-dihydrothiazol-2-yl)phenyl)piperazin-1-yl)propoxy)-3a,4,7,7a-tetrahydro-1H-4,7-epoxyisoindole-1,3(2H)-dione (**FG-11**).

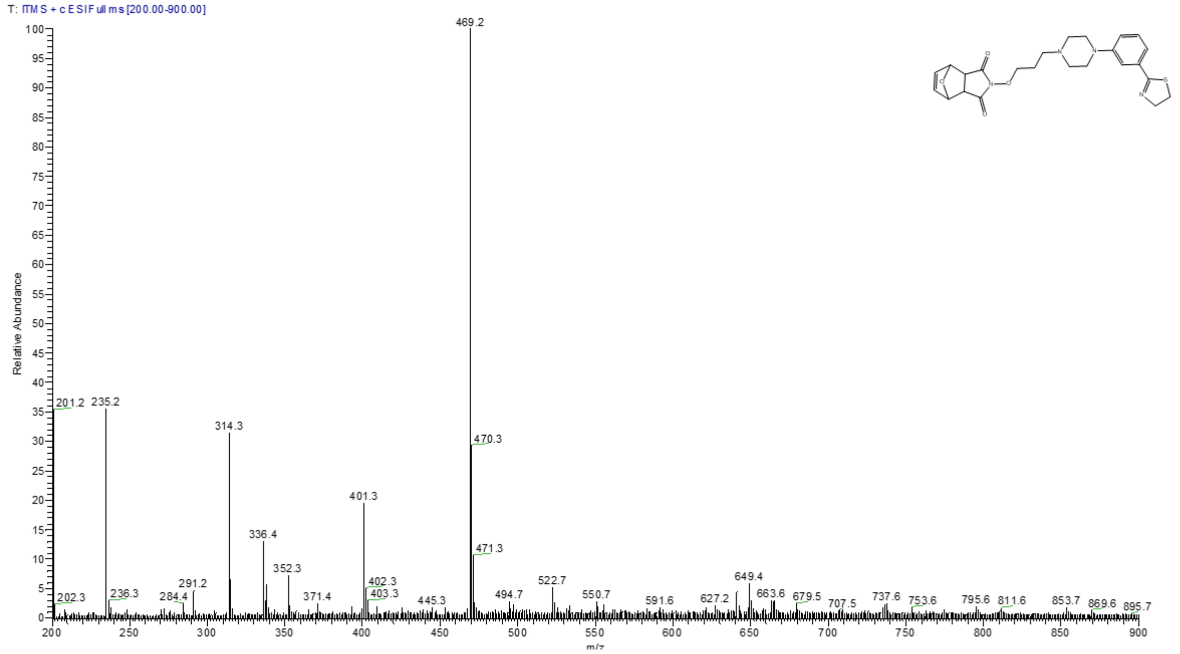

Figure S33: MS of 2-(3-(4-(3-(4,5-dihydrothiazol-2-yl)phenyl)piperazin-1-yl)propoxy)-3a,4,7,7a-tetrahydro-1H-4,7-epoxyisoindole-1,3(2H)-dione (**FG-11**).

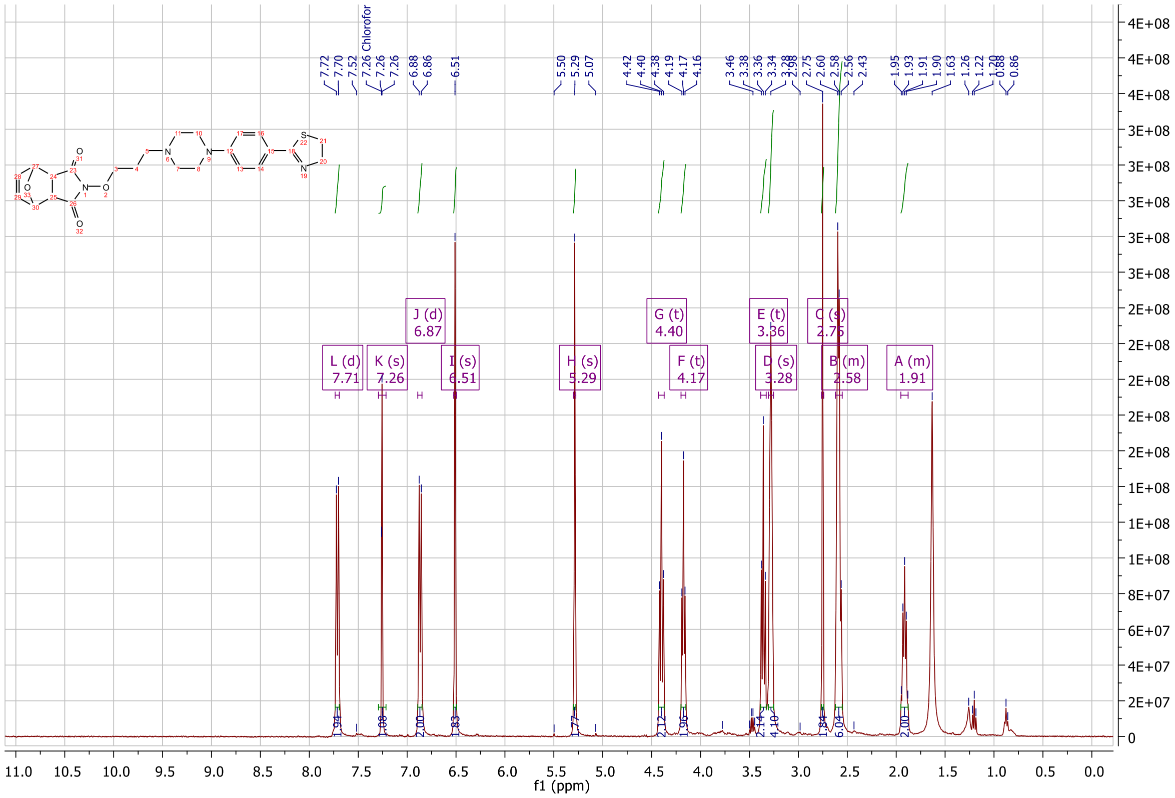

Figure S34: ^{1}H-NMR of 2-(3-(4-(4-(4,5-dihydrothiazol-2-yl)phenyl)piperazin-1-yl)propoxy)-3a,4,7,7a-tetrahydro-1H-4,7-epoxyisoindole-1,3(2H)-dione (**FG-12**).

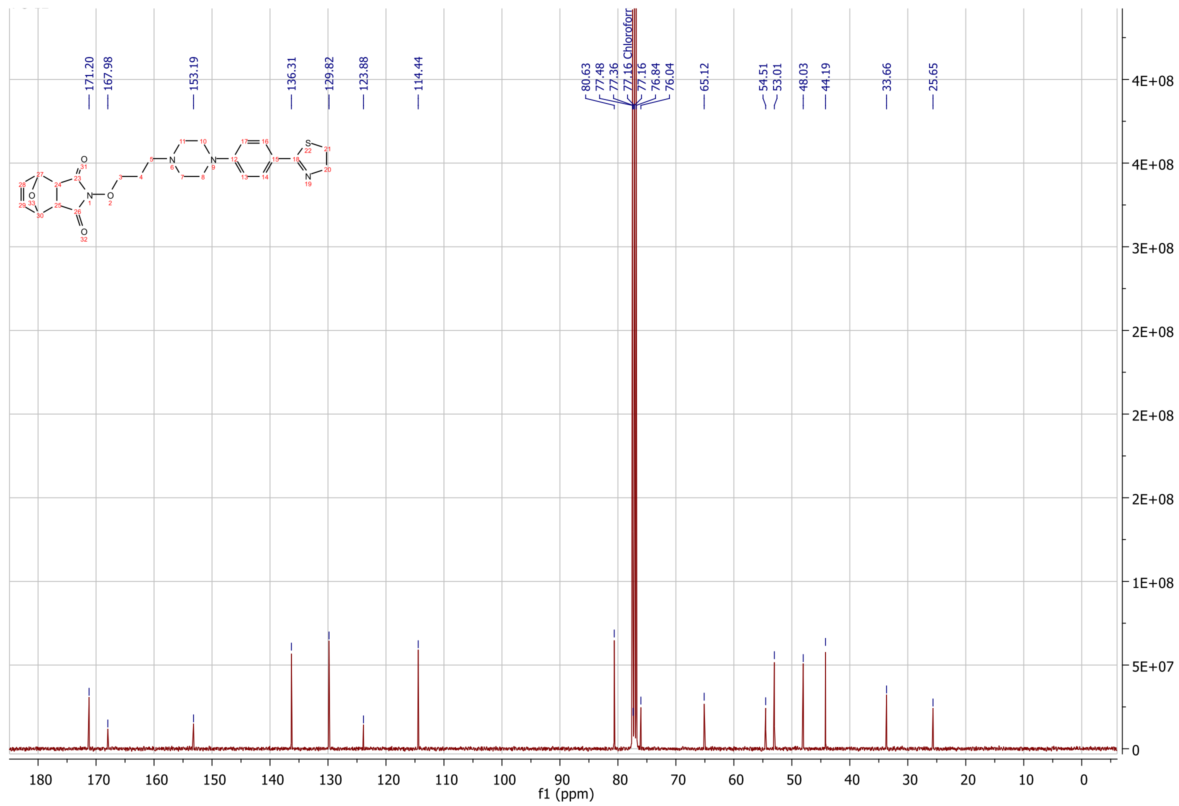

Figure S35: ^{13}C-NMR of 2-(3-(4-(4-(4,5-dihydrothiazol-2-yl)phenyl)piperazin-1-yl)propoxy)-3a,4,7,7a-tetrahydro-1H-4,7-epoxyisoindole-1,3(2H)-dione (**FG-12**).

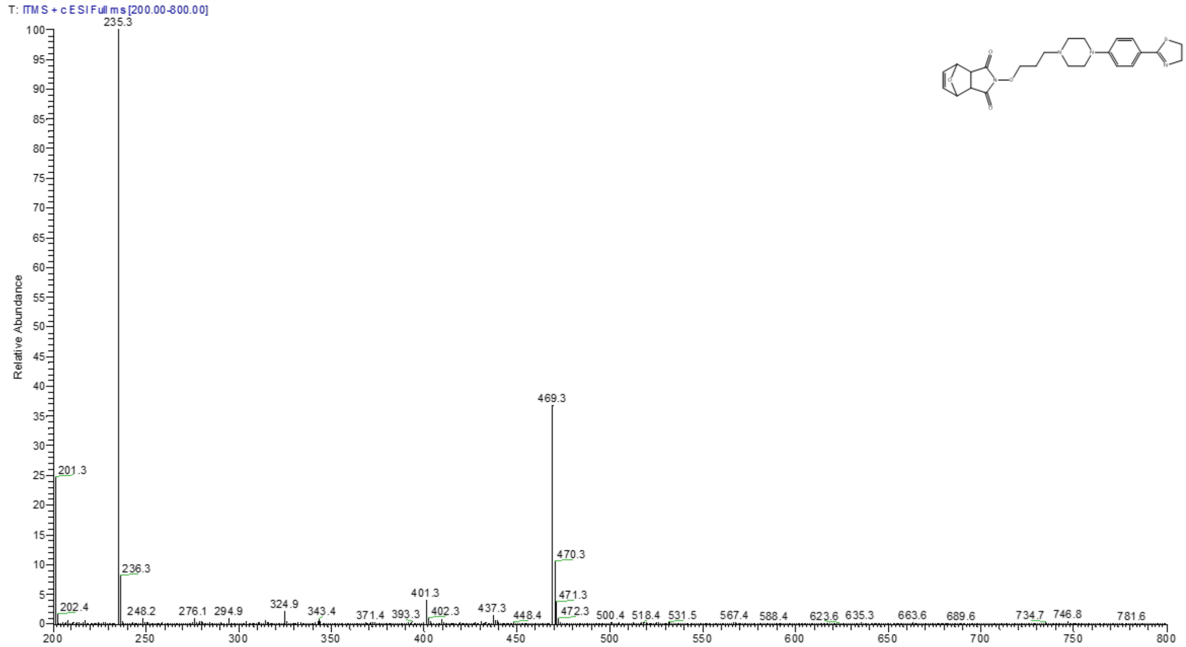

Figure S36: MS of 2-(3-(4-(4-(4,5-dihydrothiazol-2-yl)phenyl)piperazin-1-yl)propoxy)-3a,4,7,7a-tetrahydro-1H-4,7-epoxyisoindole-1,3(2H)-dione (**FG-12**).

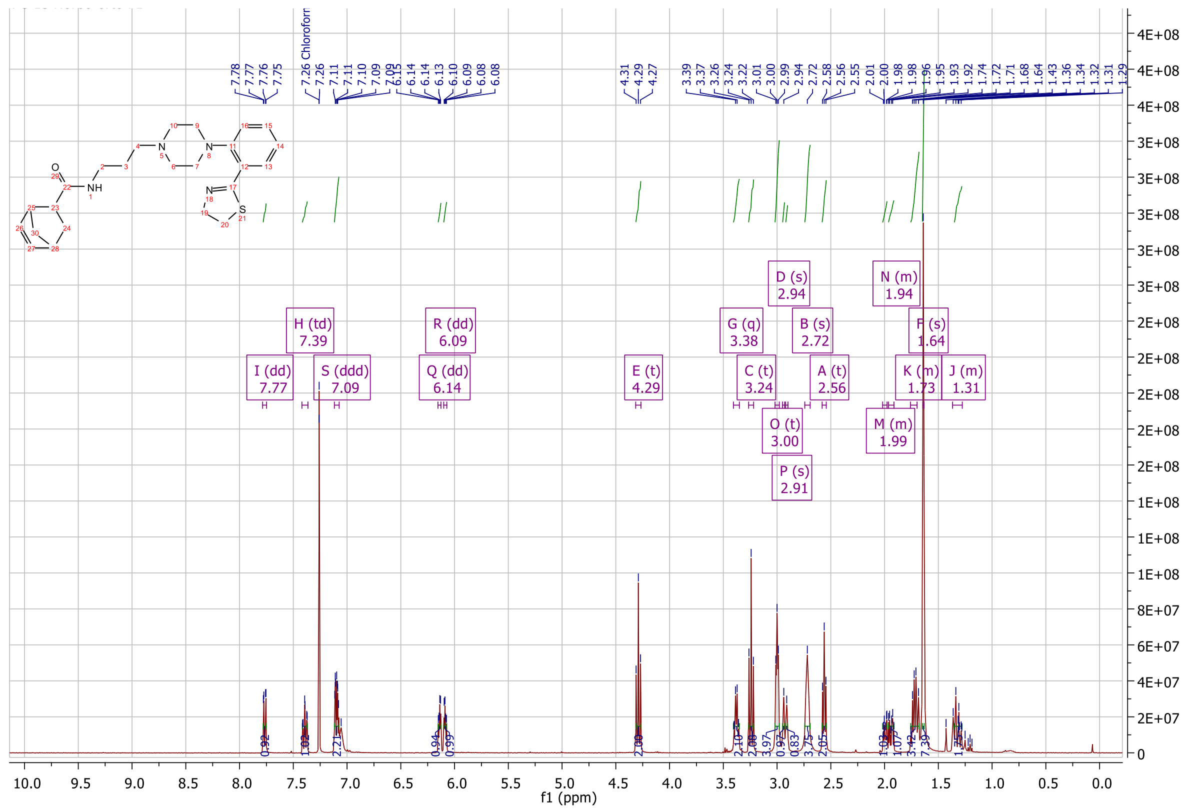

Figure S37: ^{1}H-NMR of Exo-N-(3-(4-(2-(4,5-dihydrothiazol-2-yl) phenyl) piperazin-1-yl) propyl) bicyclo [2.2.1] hept-5-ene-2-carboxamide (**FG-13**).

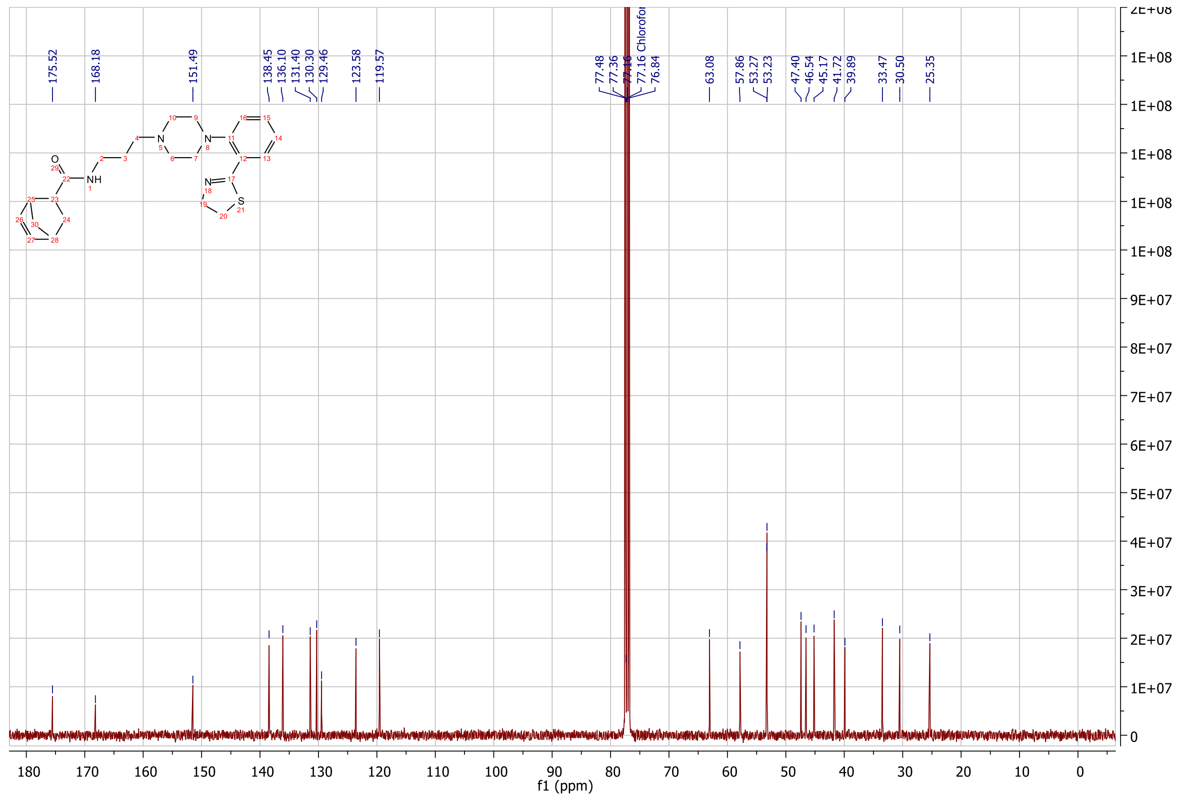

Figure S38: ^{13}C-NMR of Exo-N-(3-(4-(2-(4,5-dihydrothiazol-2-yl) phenyl) piperazin-1-yl) propyl) bicyclo [2.2.1] hept-5-ene-2-carboxamide (**FG-13**).

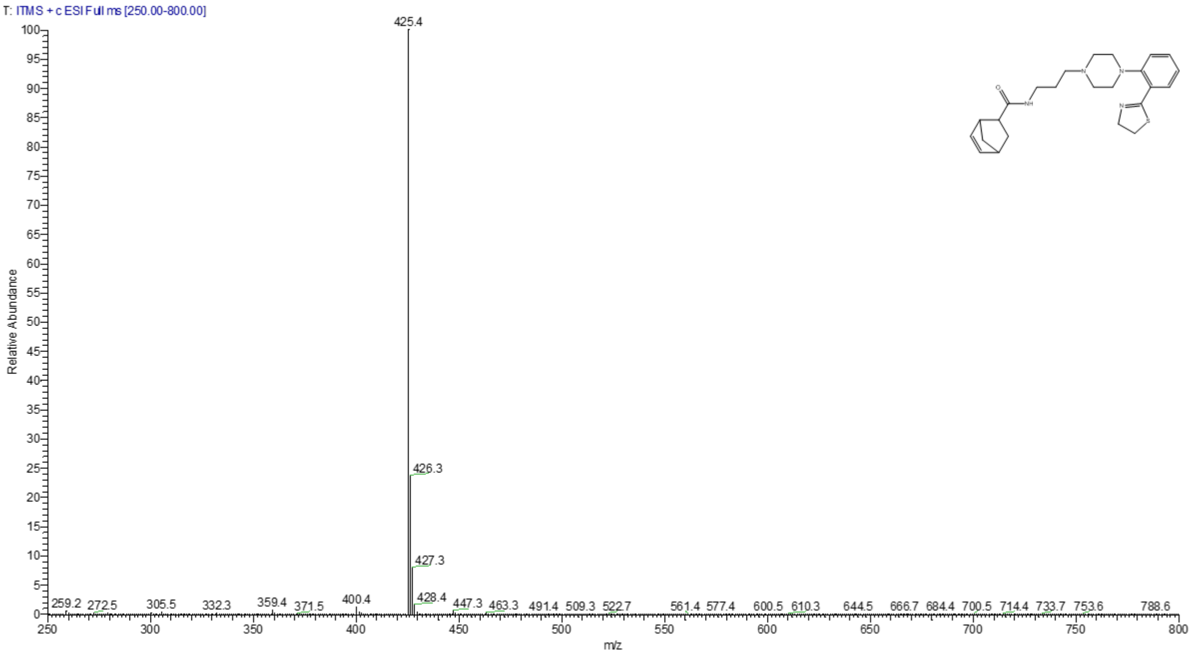

Figure S39: MS of Exo-N-(3-(4-(2-(4,5-dihydrothiazol-2-yl) phenyl) piperazin-1-yl) propyl) bicyclo [2.2.1] hept-5-ene-2-carboxamide (**FG-13**).

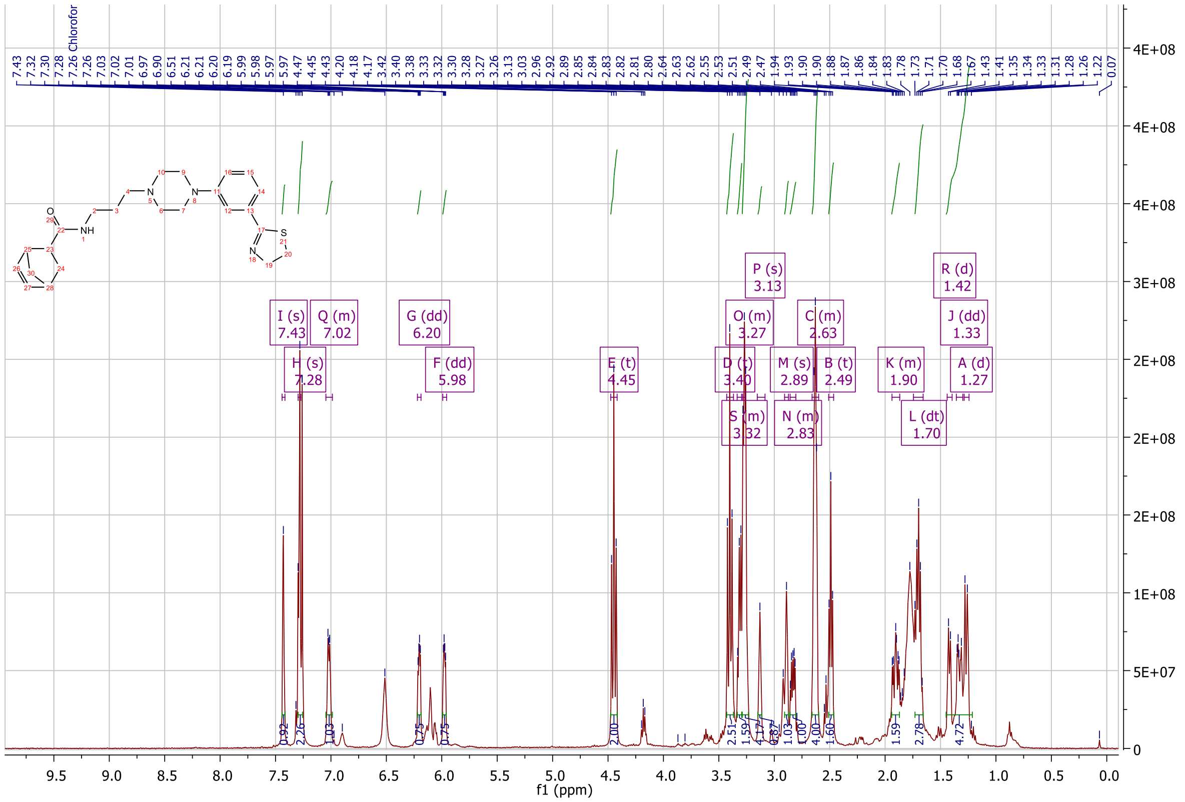

Figure S40: ^{1}H-NMR of Exo-N-(3-(4-(3-(4,5-dihydrothiazol-2-yl) phenyl) piperazin-1-yl) propyl) bicyclo [2.2.1] hept-5-ene-2-carboxamide (**FG-14**).

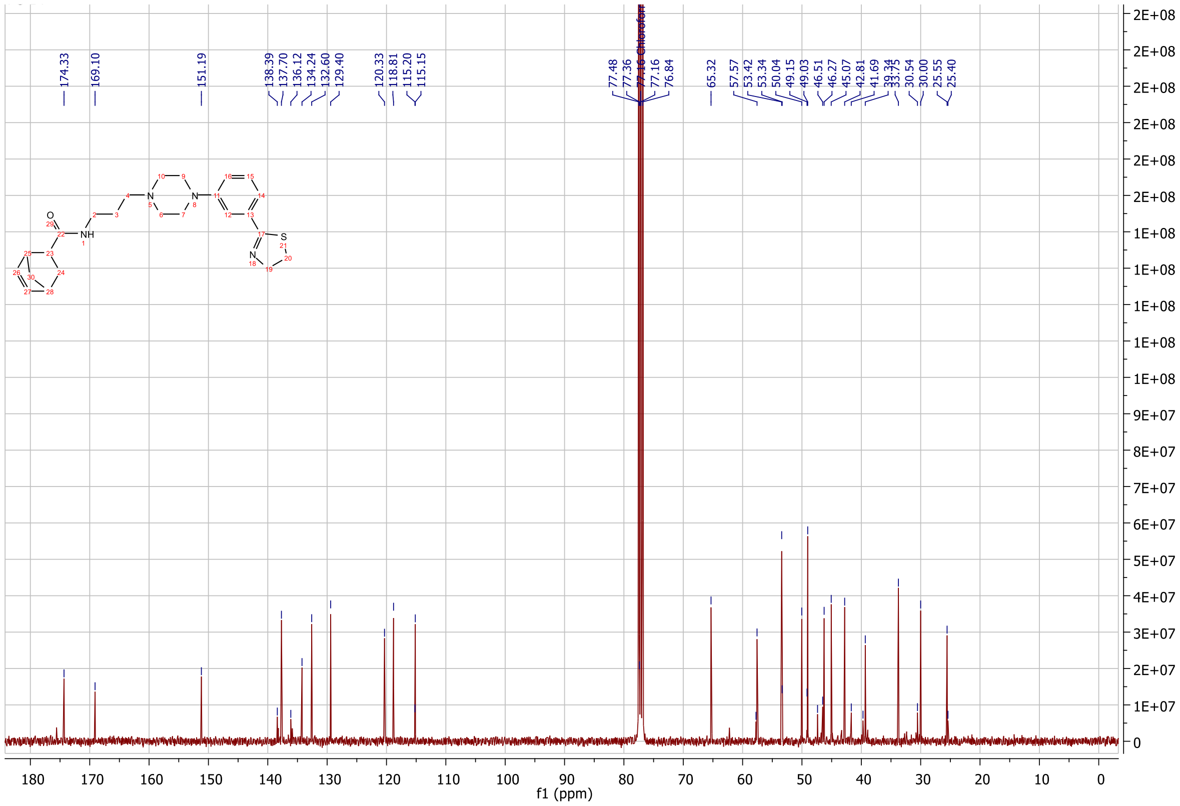

Figure S41: ^{13}C-NMR of Exo-N-(3-(4-(3-(4,5-dihydrothiazol-2-yl) phenyl) piperazin-1-yl) propyl) bicyclo [2.2.1] hept-5-ene-2-carboxamide (**FG-14**).

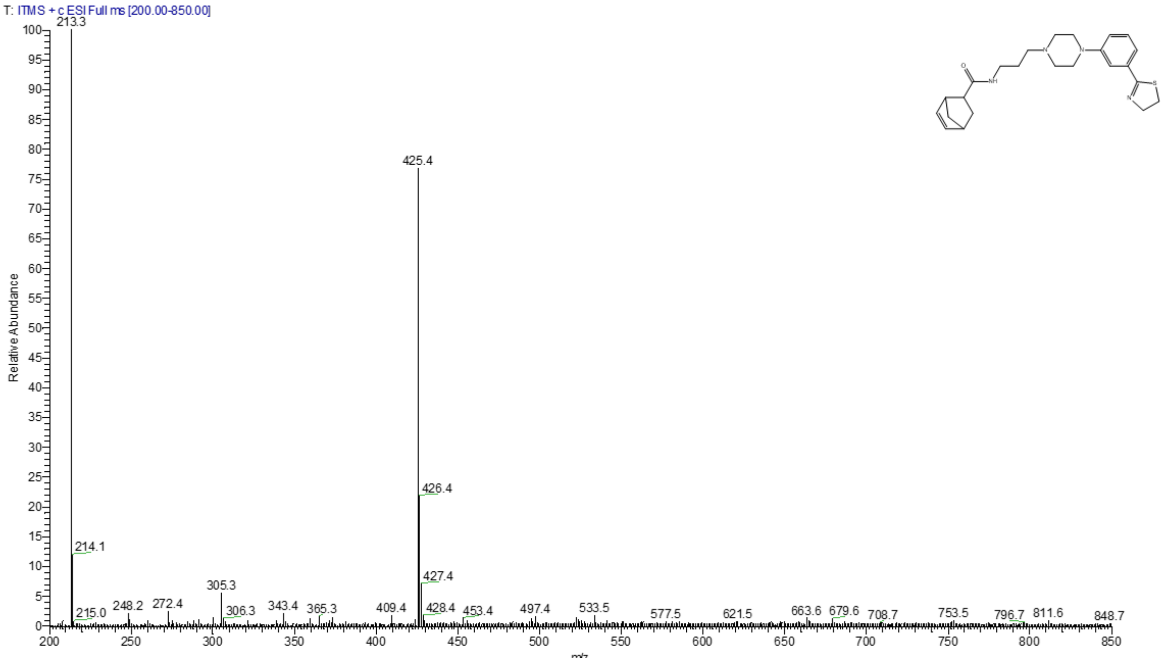

Figure S42: MS of Exo-N-(3-(4-(3-(4,5-dihydrothiazol-2-yl) phenyl) piperazin-1-yl) propyl) bicyclo [2.2.1] hept-5-ene-2-carboxamide (**FG-14**).

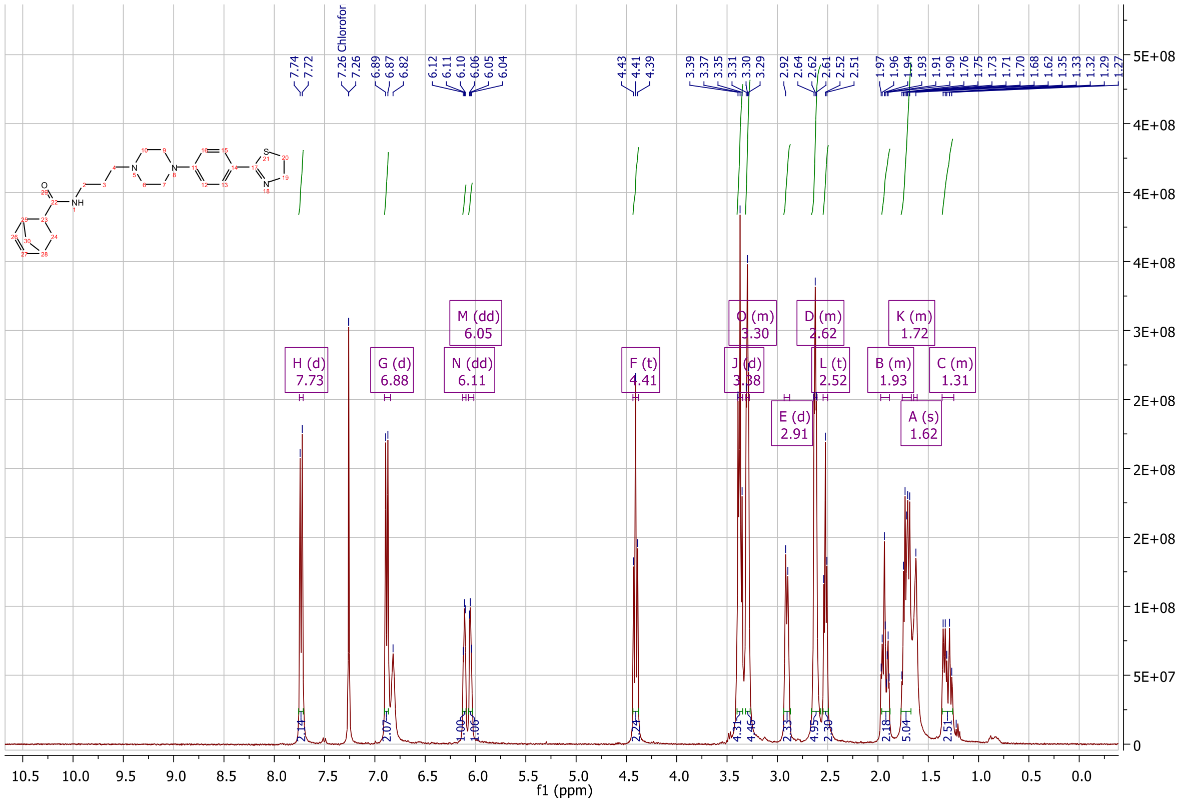

Figure S43: ^{1}H-NMR of Exo- N-(3-(4-(4-(4,5-dihydrothiazol-2-yl) phenyl) piperazin-1-yl) propyl) bicyclo [2.2.1] hept-5-ene-2-carboxamide (**FG-15**).

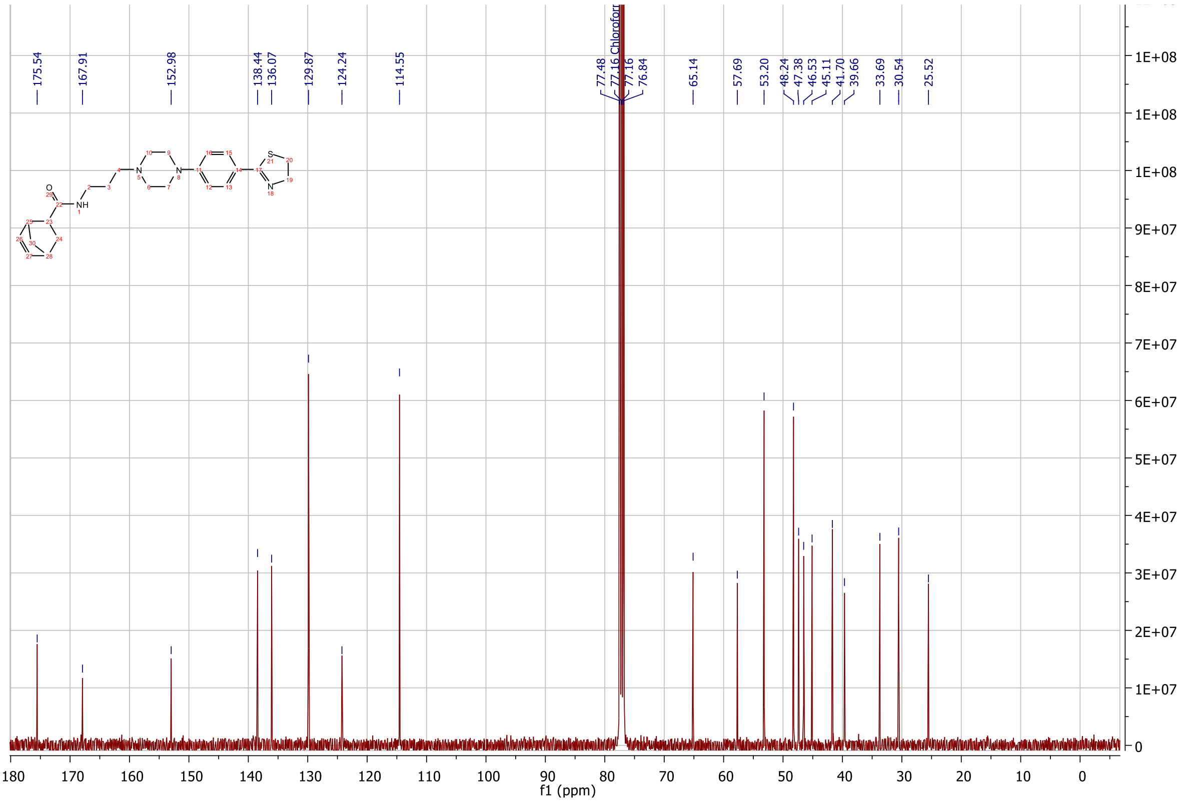

Figure S44: ^{13}C-NMR of Exo- N-(3-(4-(4-(4,5-dihydrothiazol-2-yl) phenyl) piperazin-1-yl) propyl) bicyclo [2.2.1] hept-5-ene-2-carboxamide (**FG-15**).

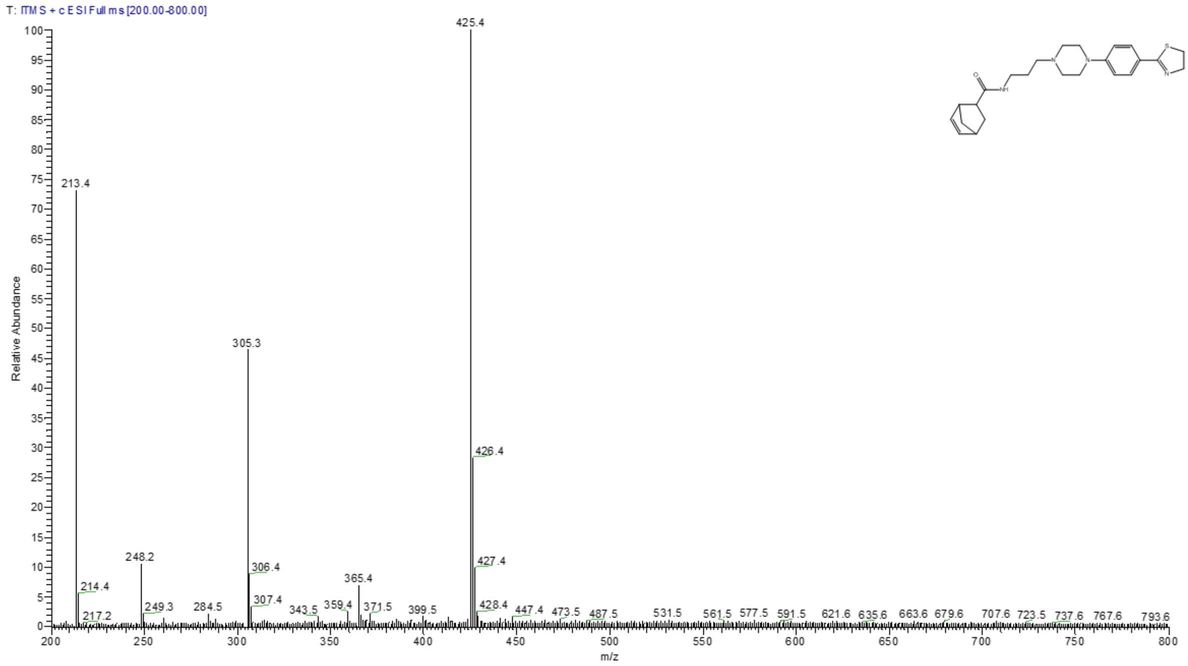

Figure S45: MS of Exo- N-(3-(4-(4-(4,5-dihydrothiazol-2-yl) phenyl) piperazin-1-yl) propyl) bicyclo [2.2.1] hept-5-ene-2-carboxamide (**FG-15**).

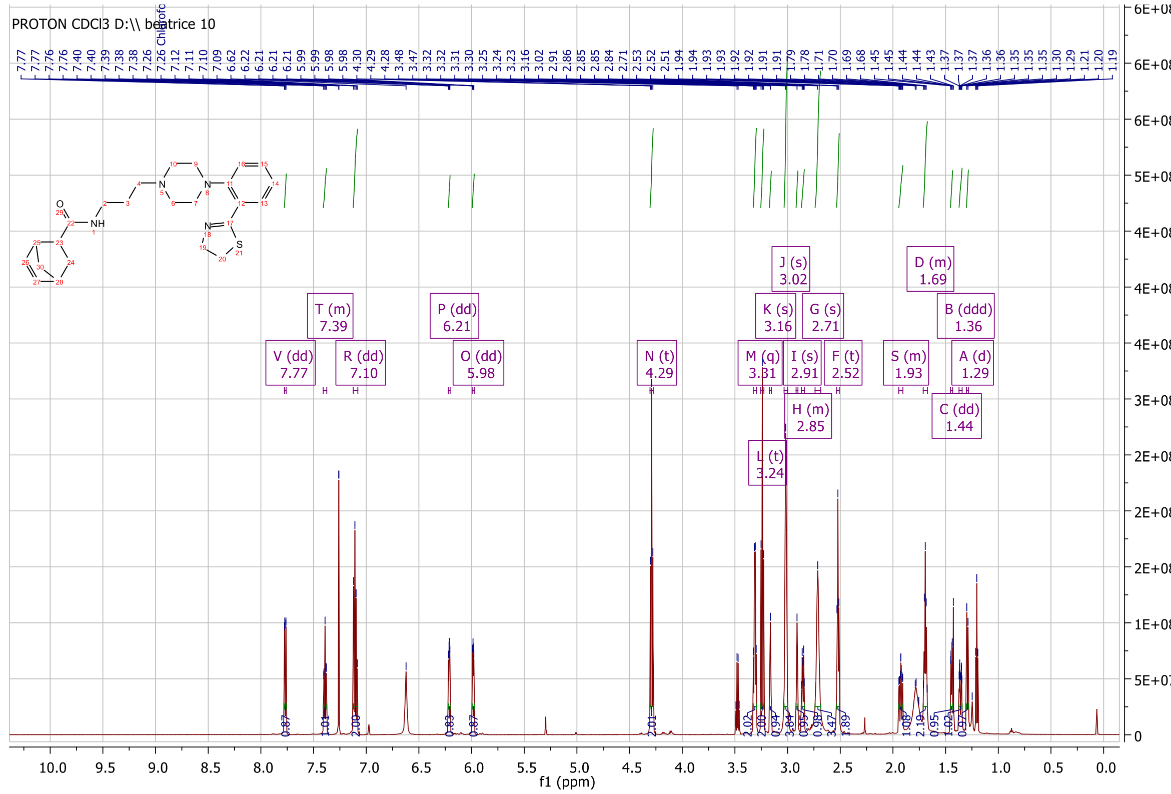

Figure S46: ^{1}H-NMR of Endo-N-(3-(4-(2-(4,5-dihydrothiazol-2-yl) phenyl) piperazin-1-yl) propyl) bicyclo[2.2.1] hept-5-ene-2-carboxamide (**FG-16**).

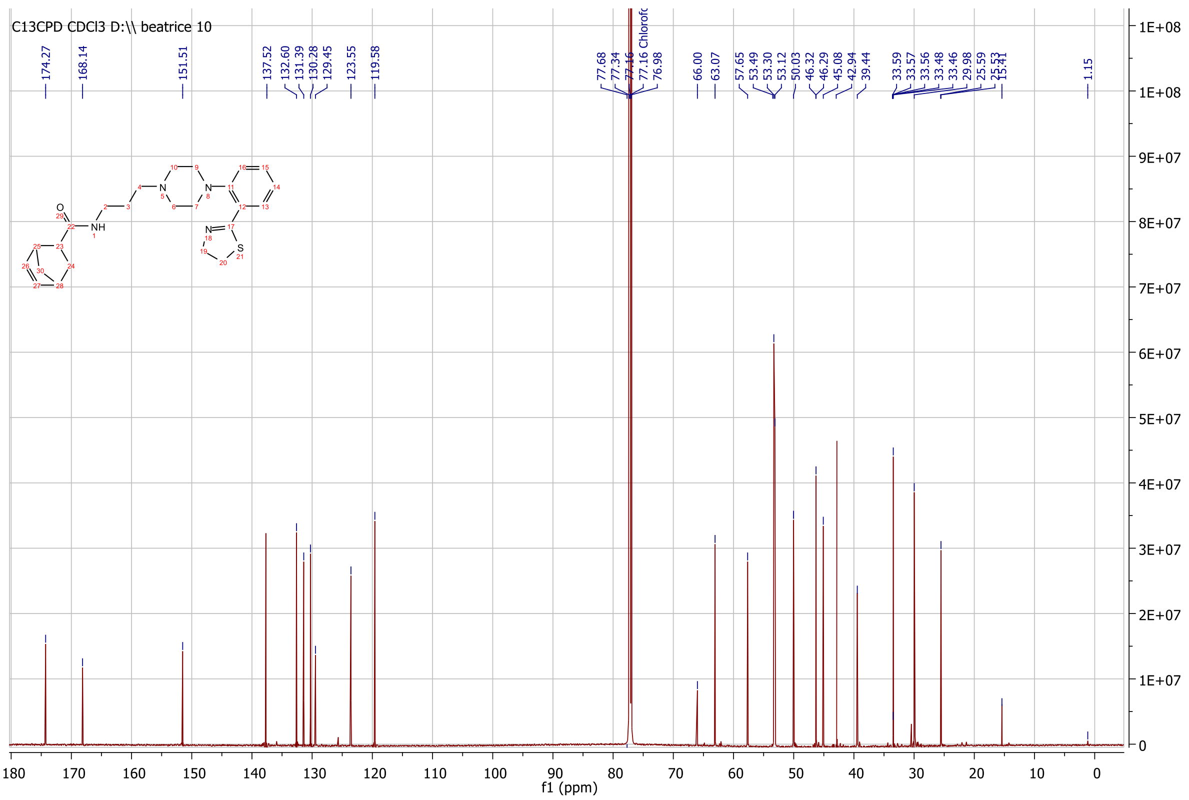

Figure S47: ^{13}C-NMR of Endo-N-(3-(4-(2-(4,5-dihydrothiazol-2-yl) phenyl) piperazin-1-yl) propyl) bicyclo[2.2.1] hept-5-ene-2-carboxamide (**FG-16**).

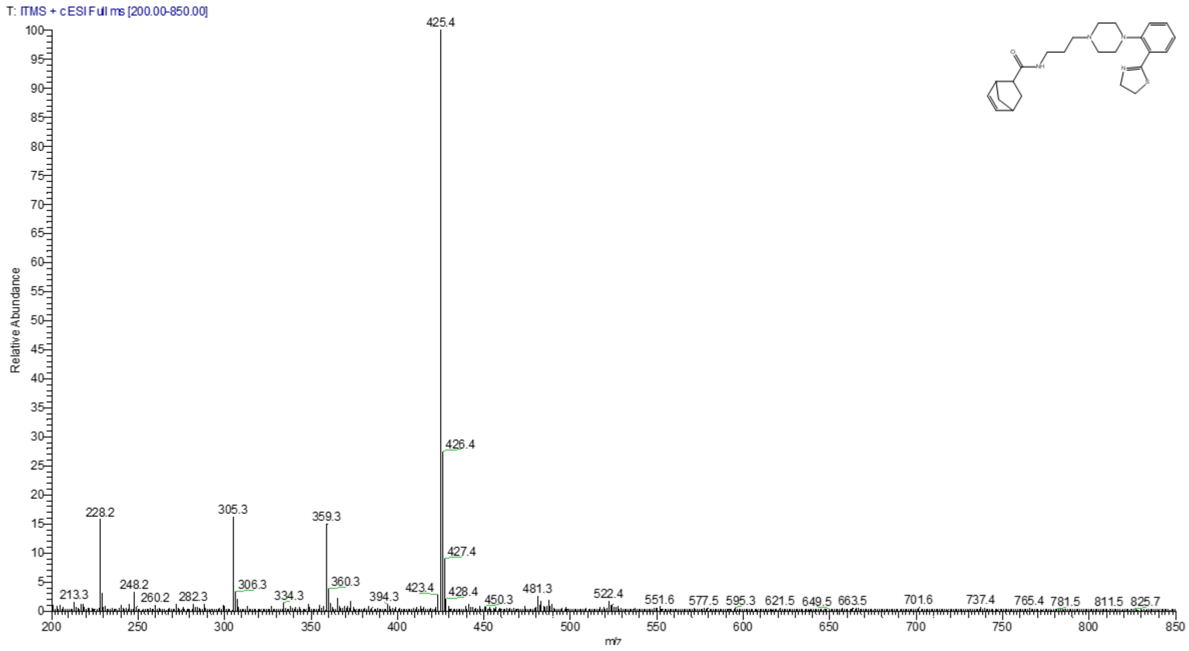

Figure S48: MS of Endo-N-(3-(4-(2-(4,5-dihydrothiazol-2-yl) phenyl) piperazin-1-yl) propyl) bicyclo[2.2.1] hept-5-ene-2-carboxamide (**FG-16**).

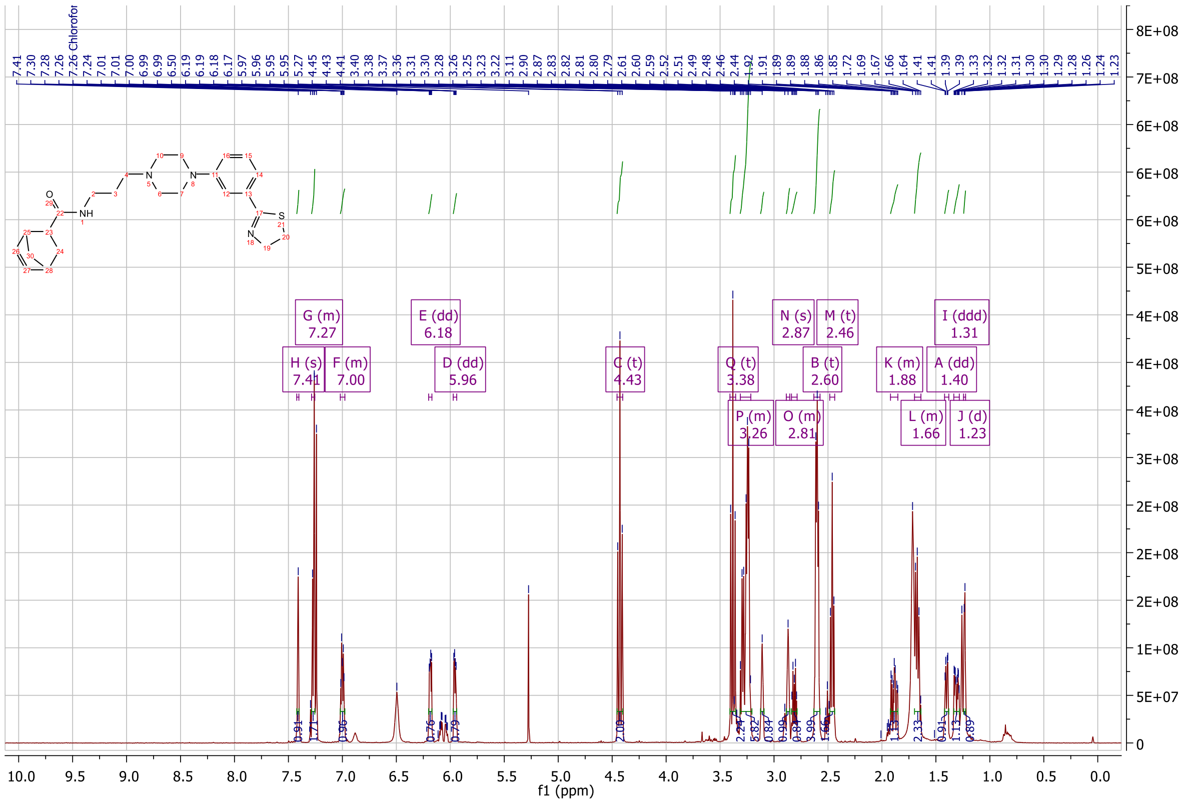

Figure S49: ¹H-NMR of Endo-N-(3-(4-(3-(4,5-dihydrothiazol-2-yl) phenyl) piperazin-1-yl) propyl) bicyclo[2.2.1]hept-5-ene-2-carboxamide (**FG-17**).

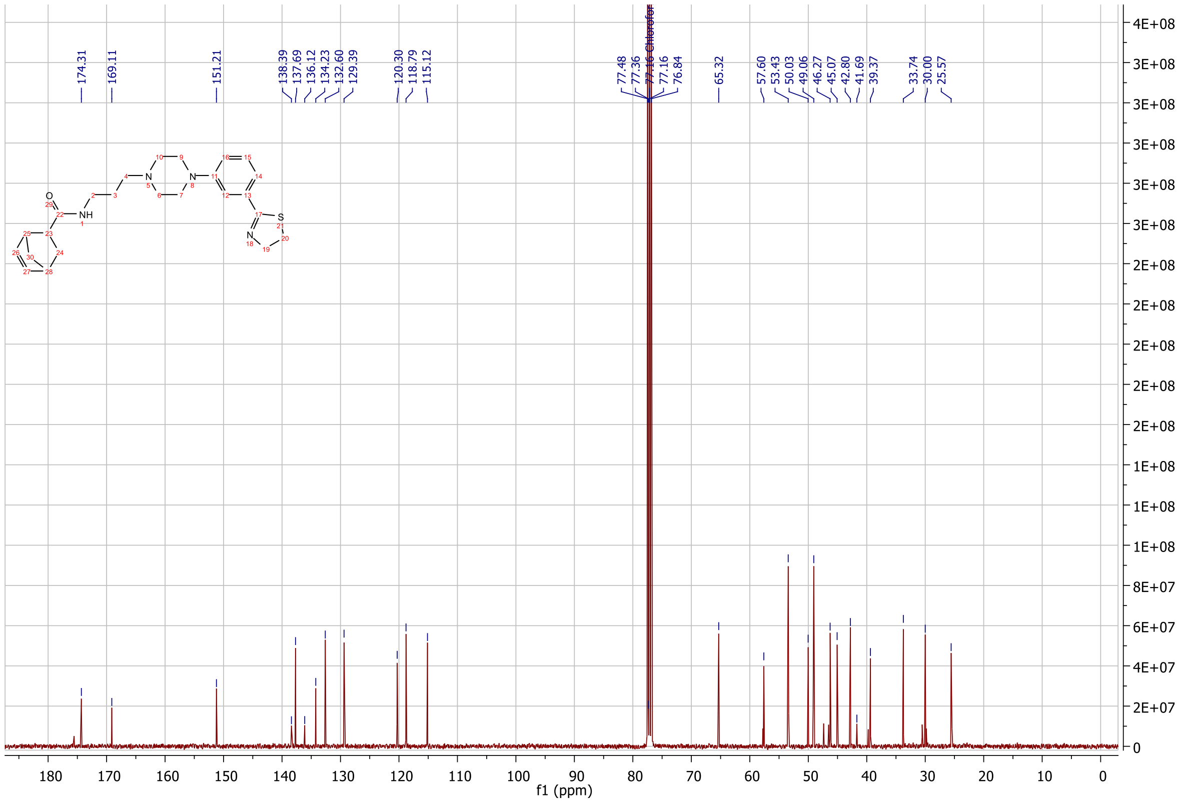

Figure S50: ¹³C-NMR of Endo-N-(3-(4-(3-(4,5-dihydrothiazol-2-yl) phenyl) piperazin-1-yl) propyl) bicyclo[2.2.1]hept-5-ene-2-carboxamide (**FG-17**).

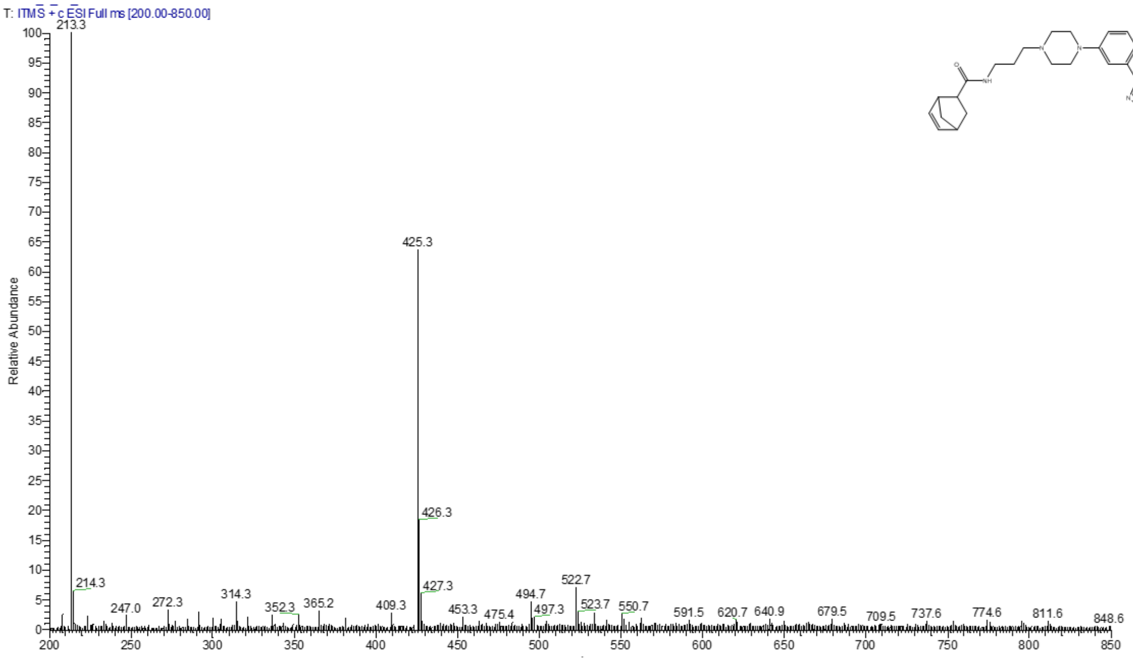

Figure S51: MS of Endo-N-(3-(4-(3-(4,5-dihydrothiazol-2-yl) phenyl) piperazin-1-yl) propyl) bicyclo[2.2.1]hept-5-ene-2-carboxamide (**FG-17**).

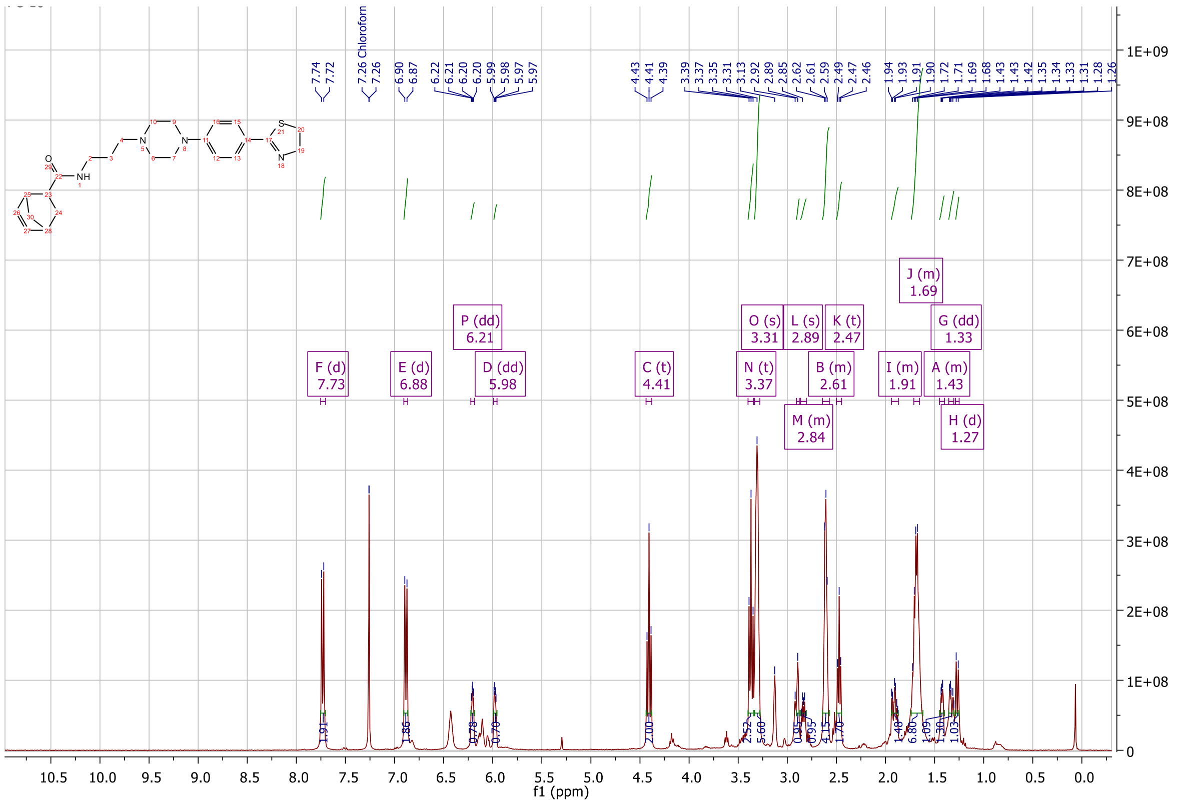

Figure S52: ¹H-NMR of Endo- N-(3-(4-(4-(4,5-dihydrothiazol-2-yl) phenyl) piperazin-1-yl) propyl) bicyclo[2.2.1]hept-5-ene-2-carboxamide (**FG-18**).

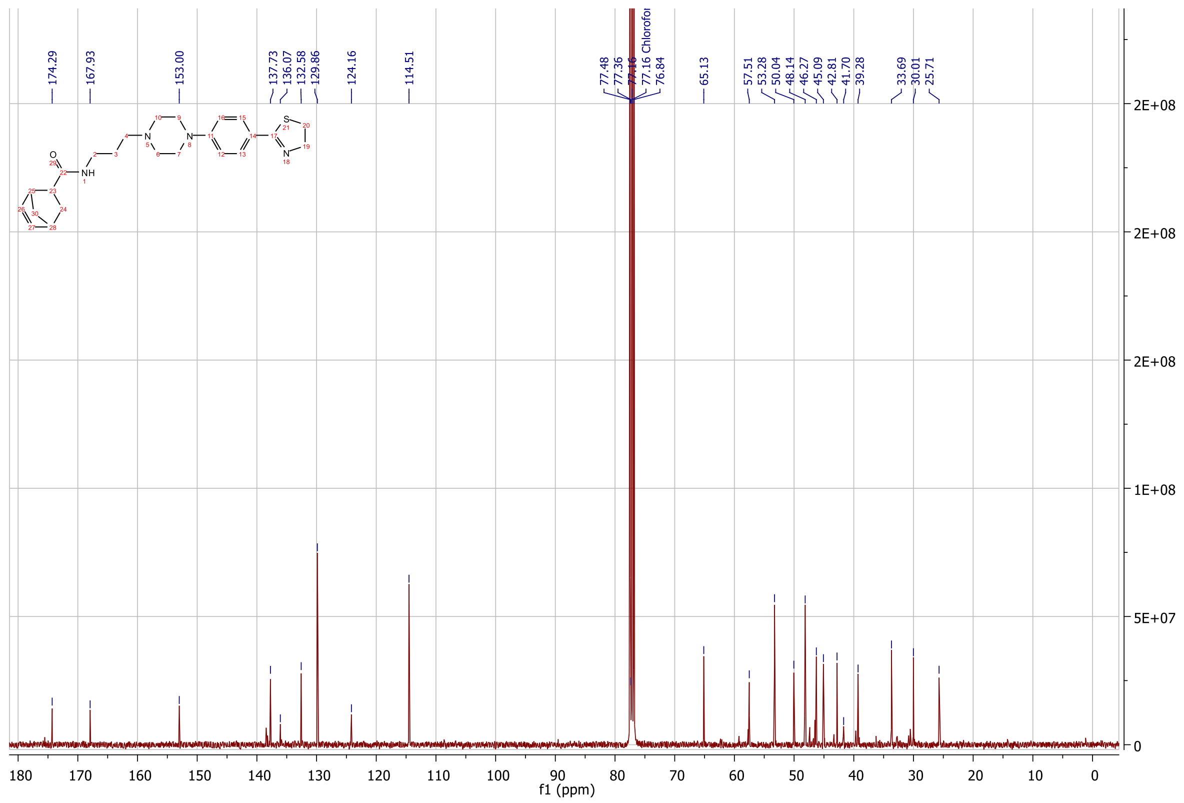

Figure S53: ^{13}C-NMR of Endo- N-(3-(4-(4-(4,5-dihydrothiazol-2-yl) phenyl) piperazin-1-yl) propyl) bicyclo[2.2.1]hept-5-ene-2-carboxamide (**FG-18**).

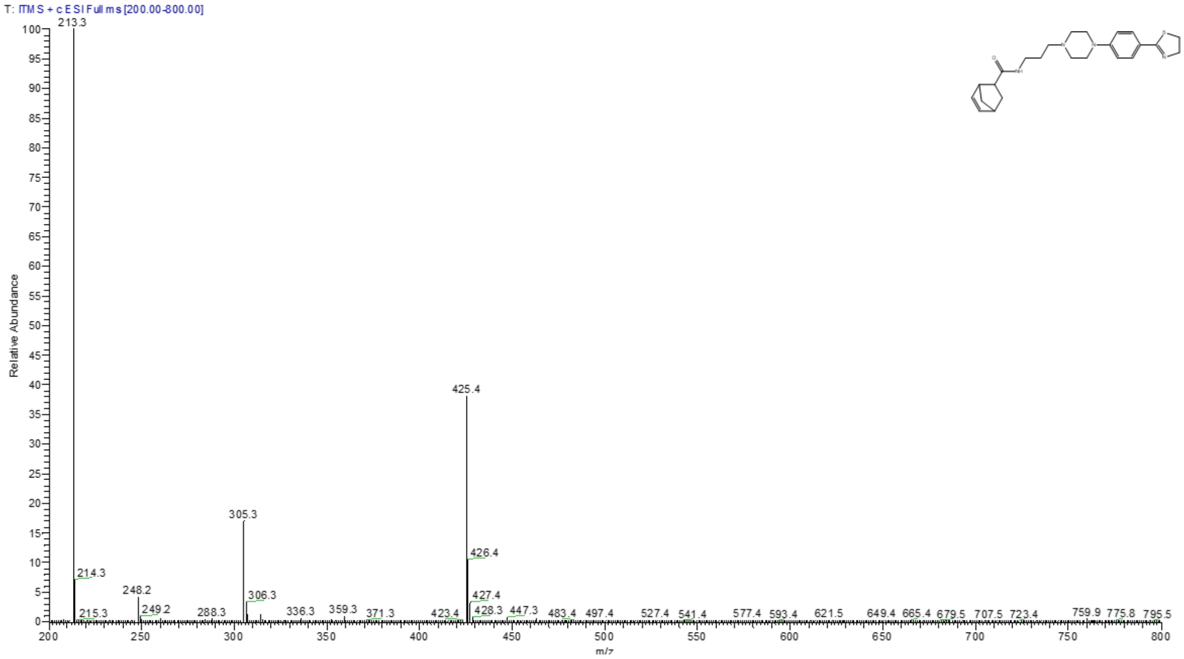

Figure S54: MS of Endo- N-(3-(4-(4-(4,5-dihydrothiazol-2-yl) phenyl) piperazin-1-yl) propyl) bicyclo[2.2.1]hept-5-ene-2-carboxamide (**FG-18**).
